# Supplementary material for: Extensive and deep sequencing of the Venter/HuRef genome for developing and benchmarking genome analysis tools
Source: Sci Data. 2018 Dec 18;5:180261. doi: 10.1038/sdata.2018.261 (PMC6298255; doi:10.1038/sdata.2018.261)
Supplement: Supplementary Information [file sdata2018261-s2.pdf]

SUPPLEMENTARY INFORMATION

FastQC reports

Short-insert (200 bp) ……………..……………………………………2

Short-insert (350 bp) ……………..……………………………..… 32

Mate-pair
2kb …………………………………………………………………. 42
5kb ...……………………………...…………………………………… 52
12kb ………………………..……………………………………..… 62

Linked-reads
R1……………………………………………………….………….…72
R2……………………………………………………….…………..…82
(Separate FastQC reports for read_1 and read_2; first 16 bp of read_1 are the “droplet” barcodes in linked-read sequencing.)

FastQC Report

Sat 31 Mar 2018
stdin

Summary

- Basic Statistics
- Per base sequence quality
- Per tile sequence quality
- Per sequence quality scores
- Per base sequence content
- Per sequence GC content
- Per base N content
- Sequence Length Distribution
- Sequence Duplication Levels
- Overrepresented sequences
- Adapter Content

Basic Statistics

Measure	Value
Filename	stdin
File type	Conventional base calls
Encoding	Sanger / Illumina 1.9
Total Sequences	3214623356
Sequences flagged as poor quality	0
Sequence length	100
%GC	40

Per base sequence quality

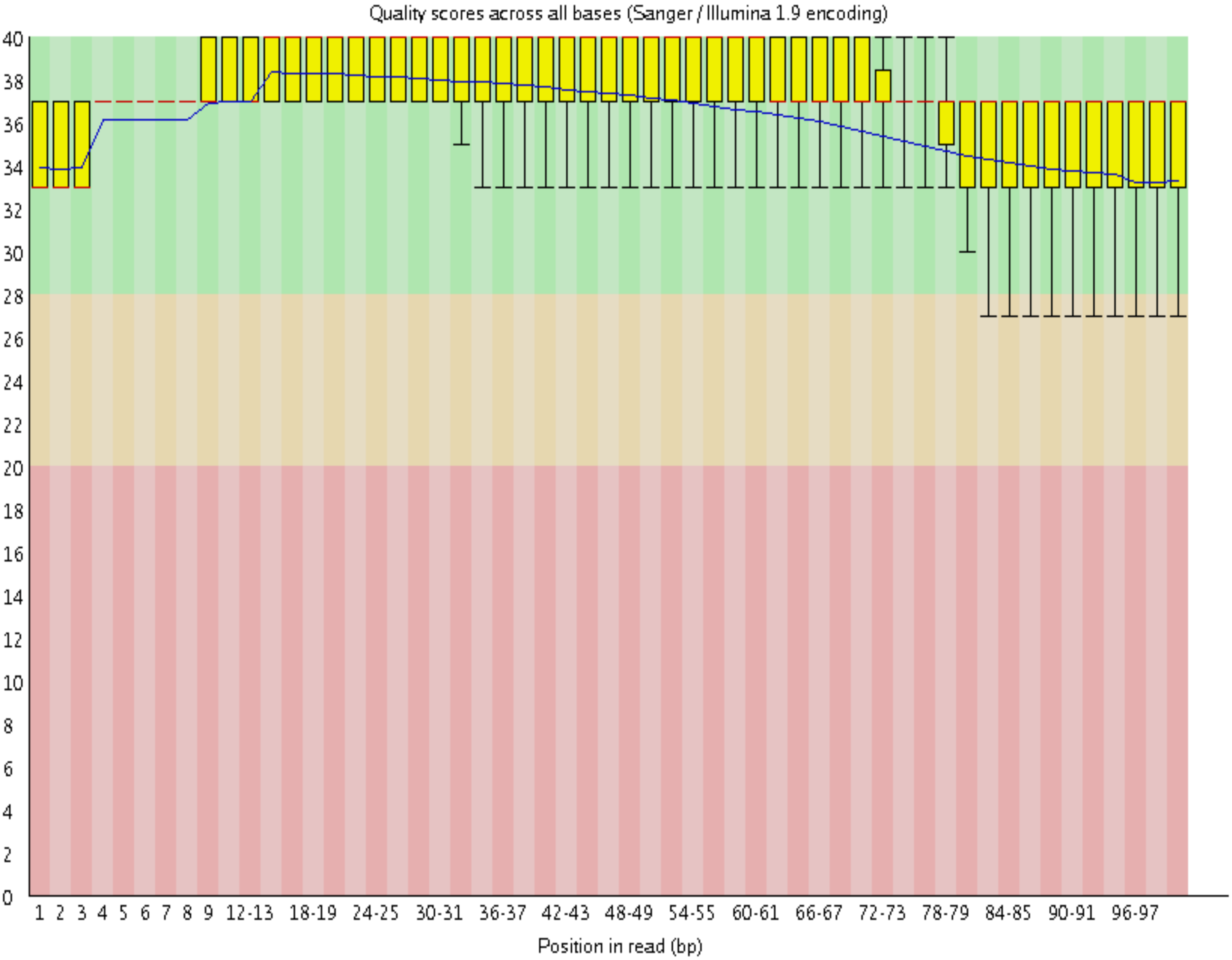

Per tile sequence quality

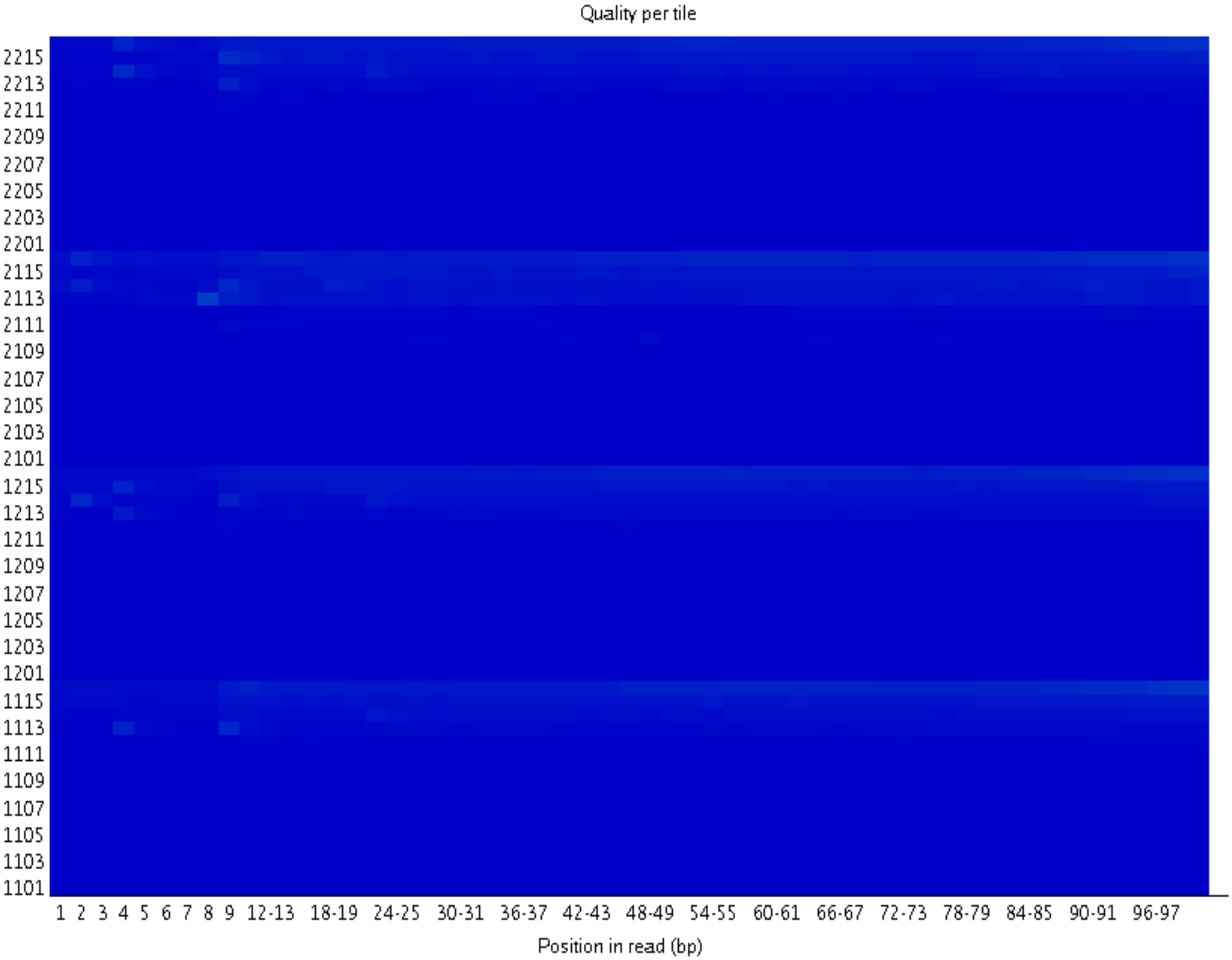

Per sequence quality scores

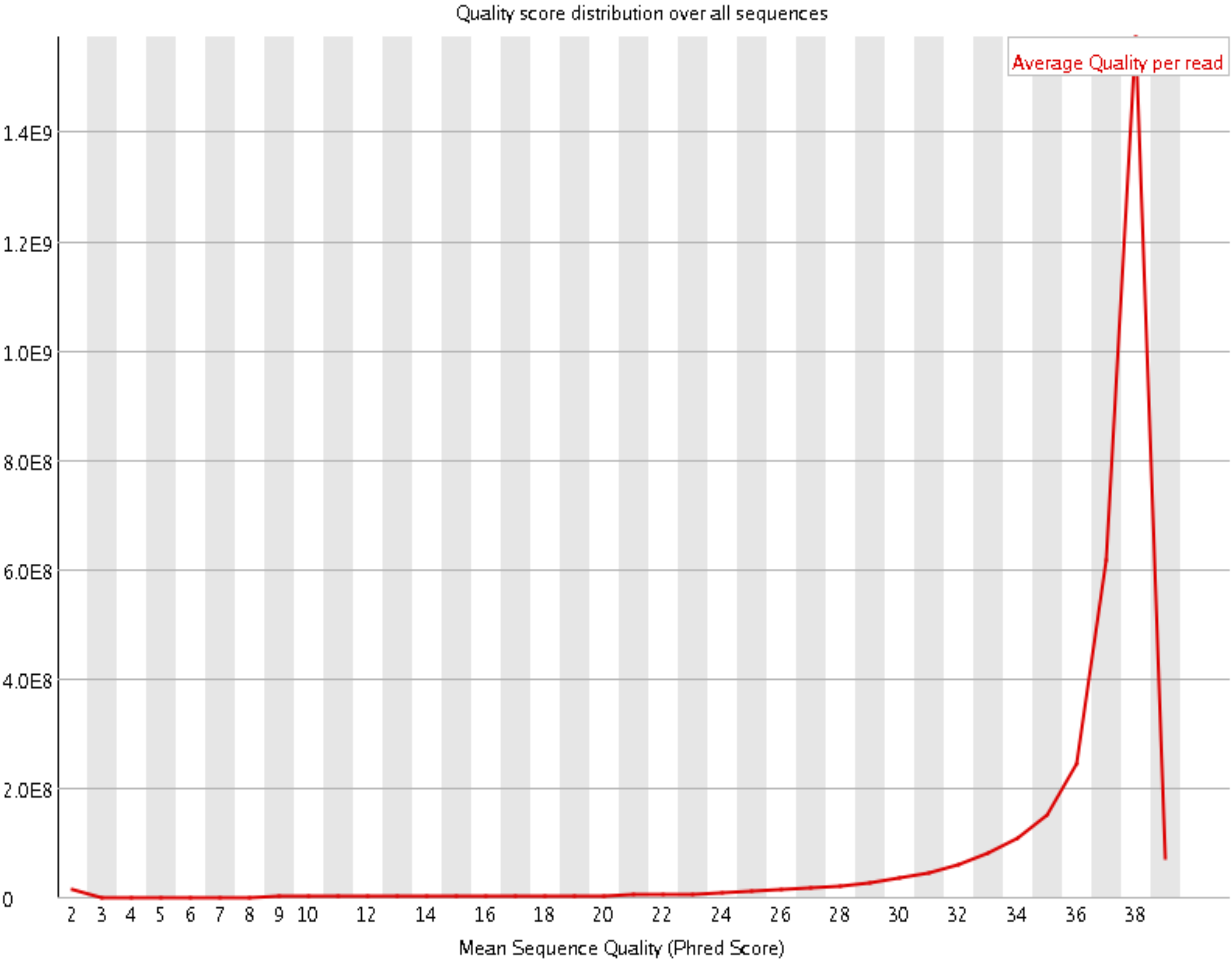

Per base sequence content

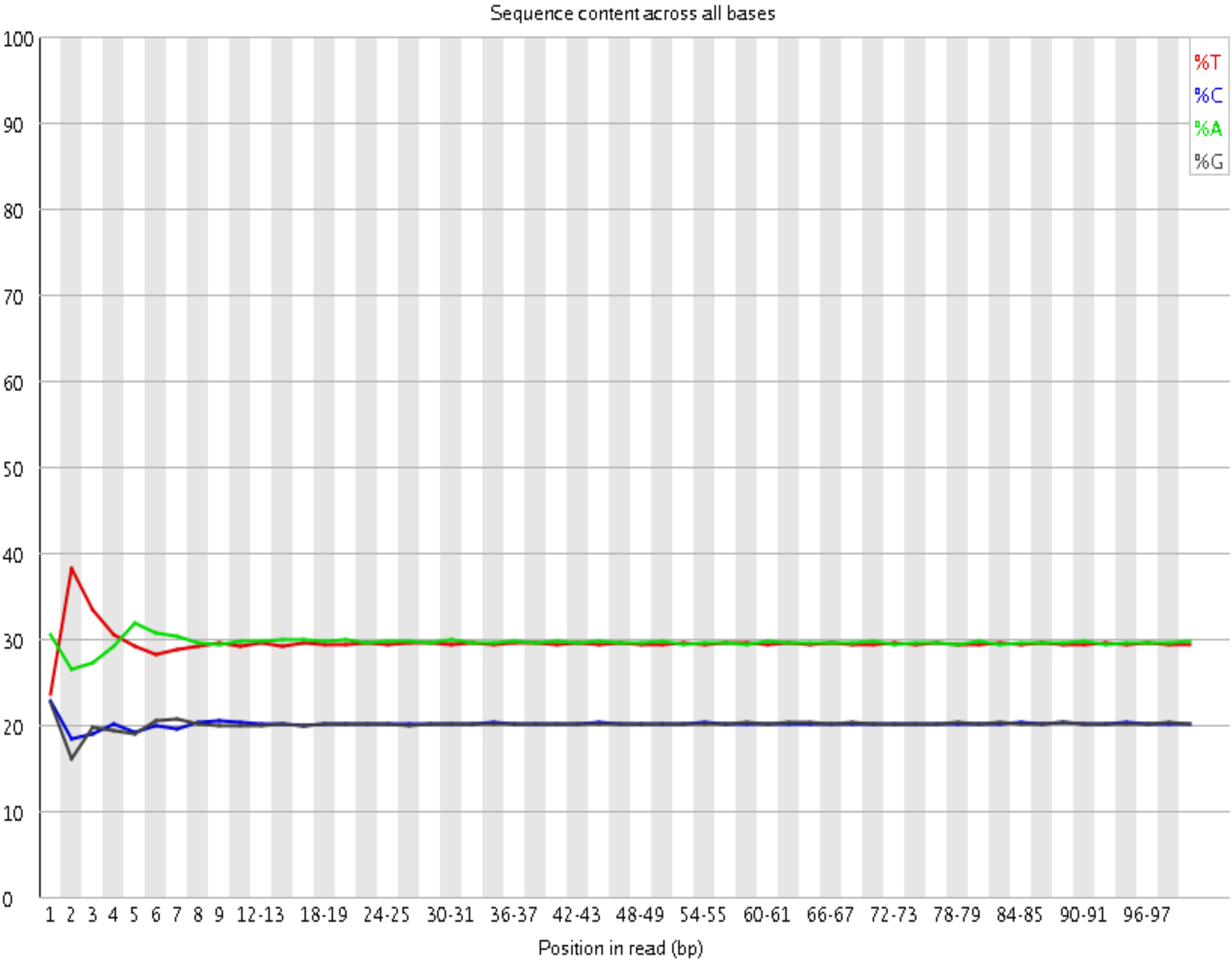

Per sequence GC content

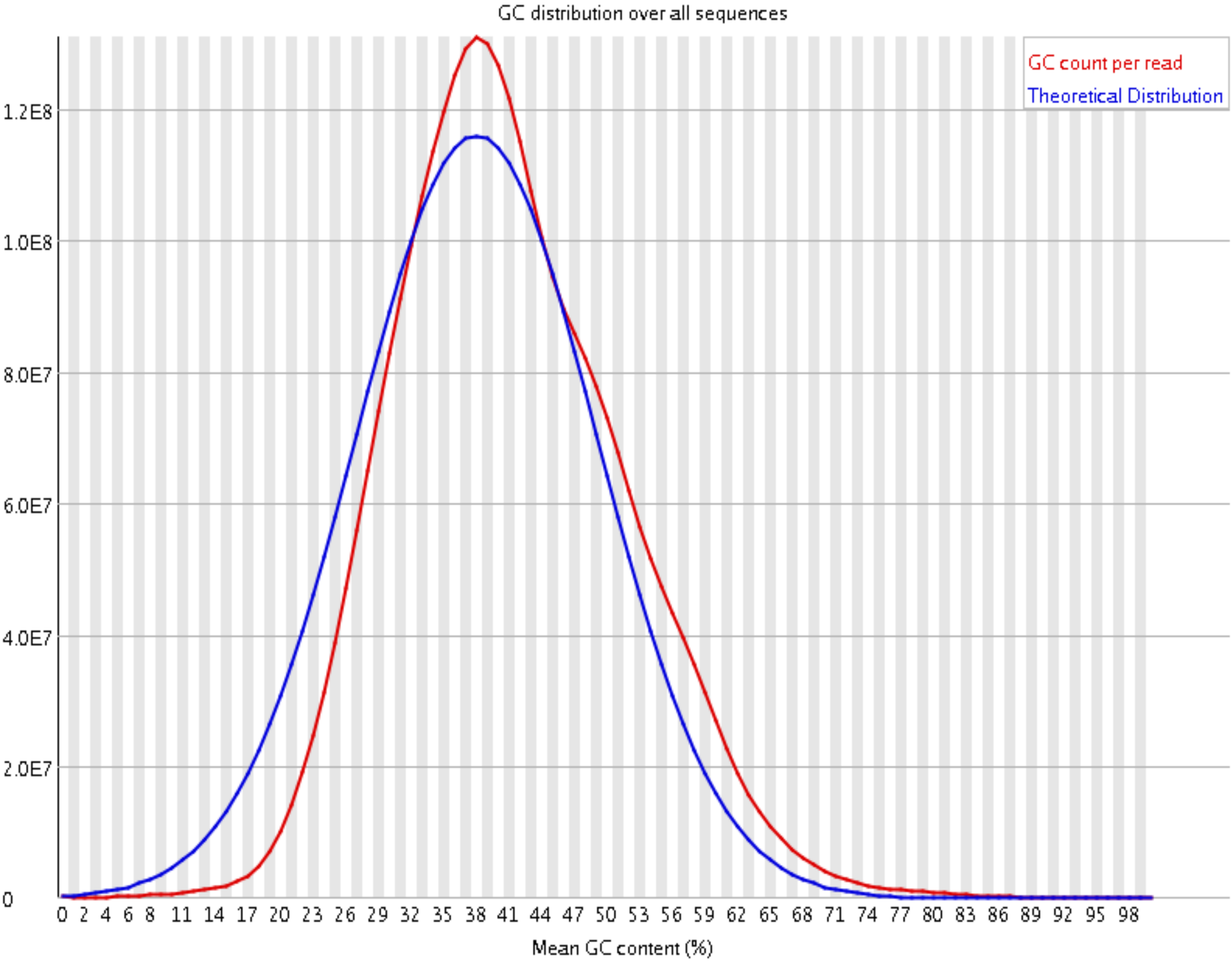

Per base N content

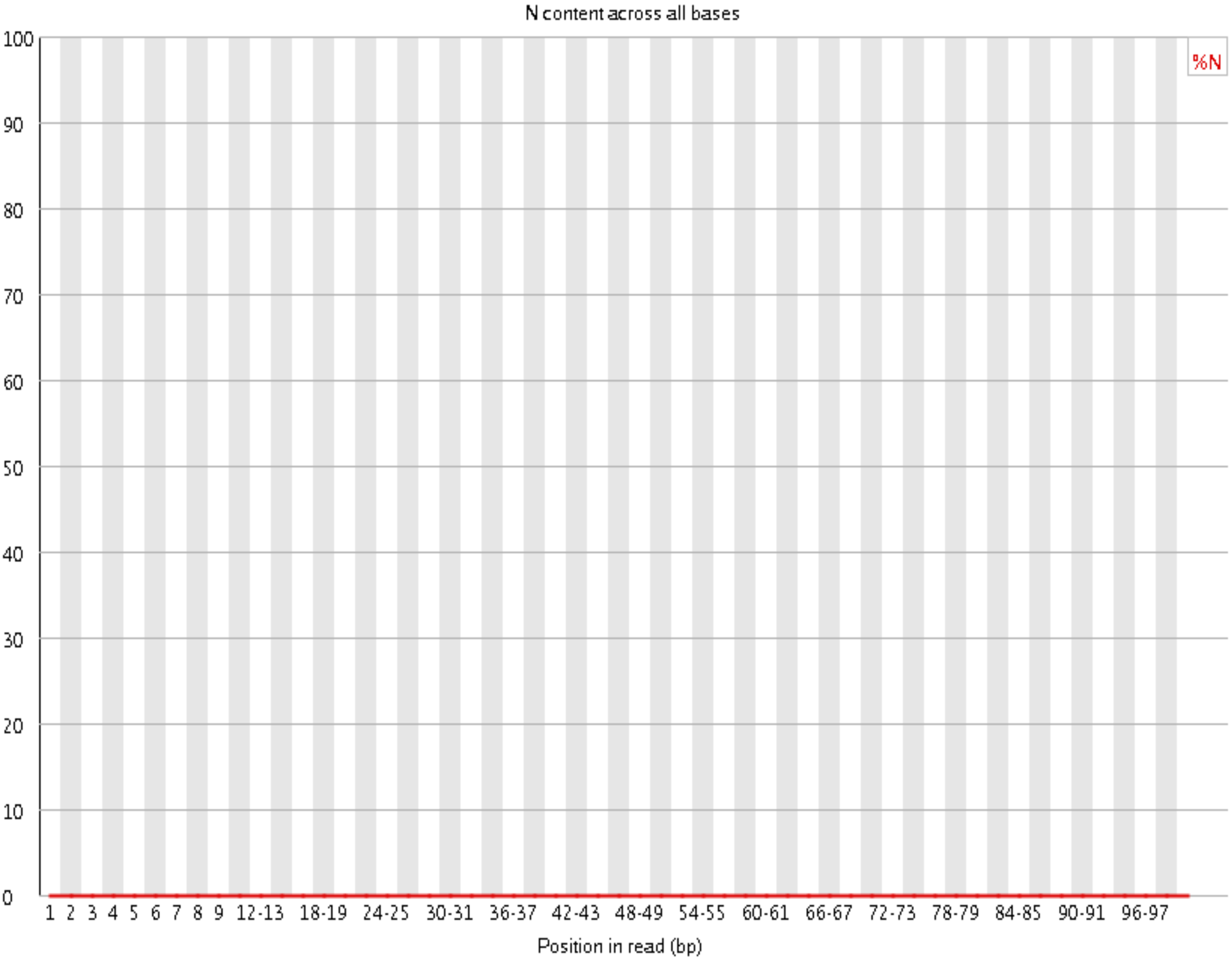

Sequence Length Distribution

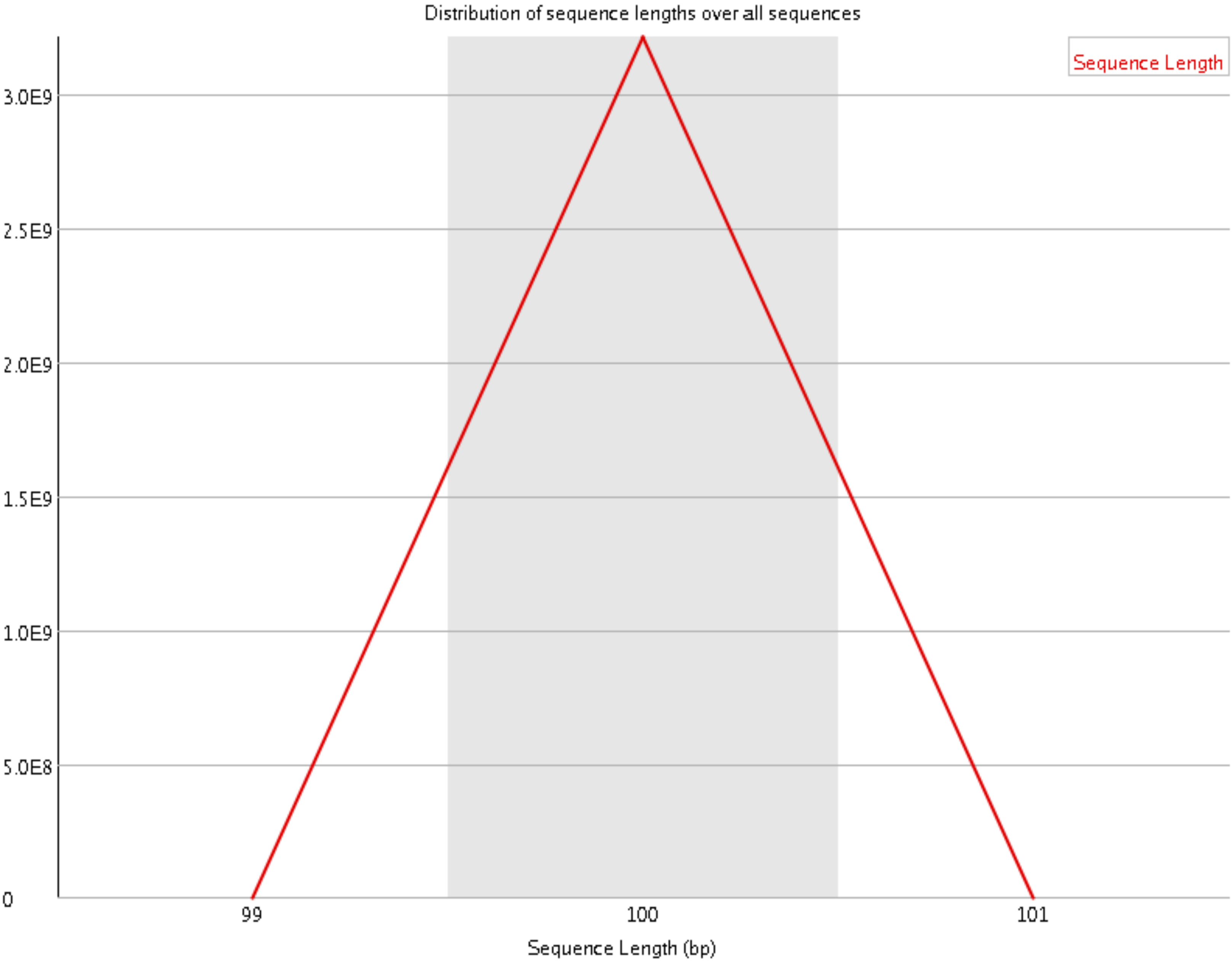

Sequence Duplication Levels

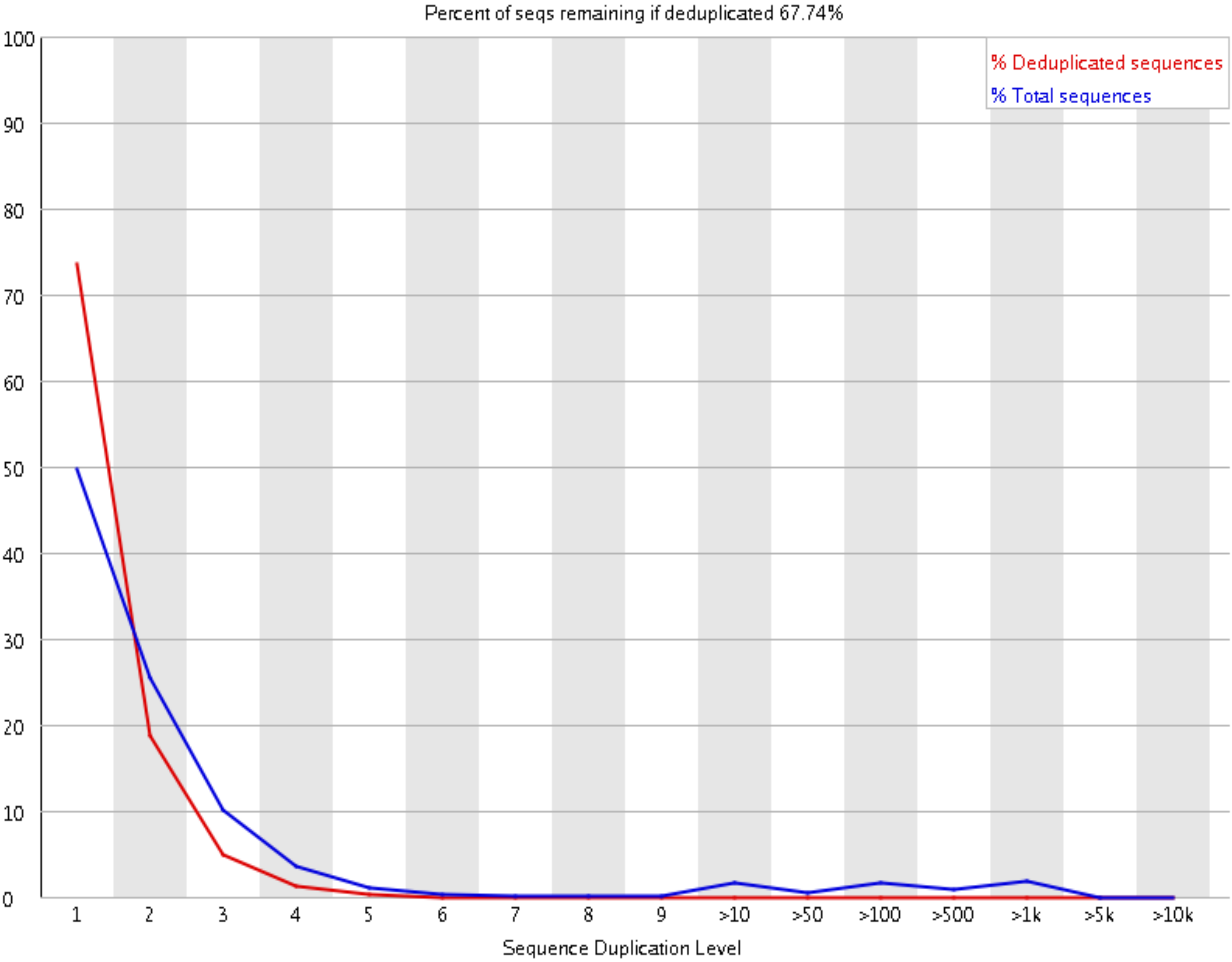

Overrepresented sequences

No overrepresented sequences

Adapter Content

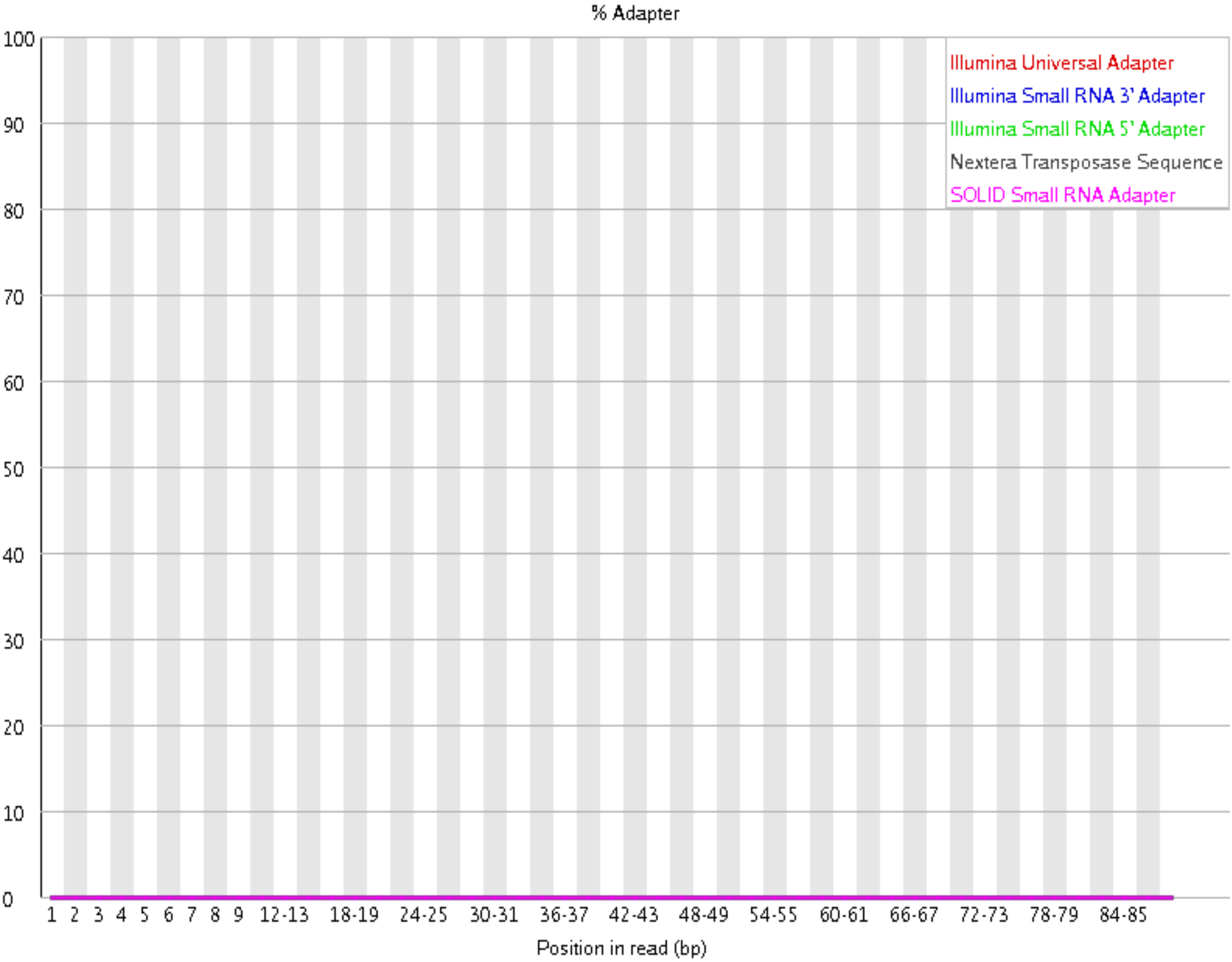

Produced by FastQC (version 0.11.7)

FastQC Report

Sat 31 Mar 2018
stdin

Summary

- Basic Statistics
- Per base sequence quality
- Per tile sequence quality
- Per sequence quality scores
- Per base sequence content
- Per sequence GC content
- Per base N content
- Sequence Length Distribution
- Sequence Duplication Levels
- Overrepresented sequences
- Adapter Content

Basic Statistics

Measure	Value
Filename	stdin
File type	Conventional base calls
Encoding	Sanger / Illumina 1.9
Total Sequences	1280576580
Sequences flagged as poor quality	0
Sequence length	100
%GC	40

Per base sequence quality

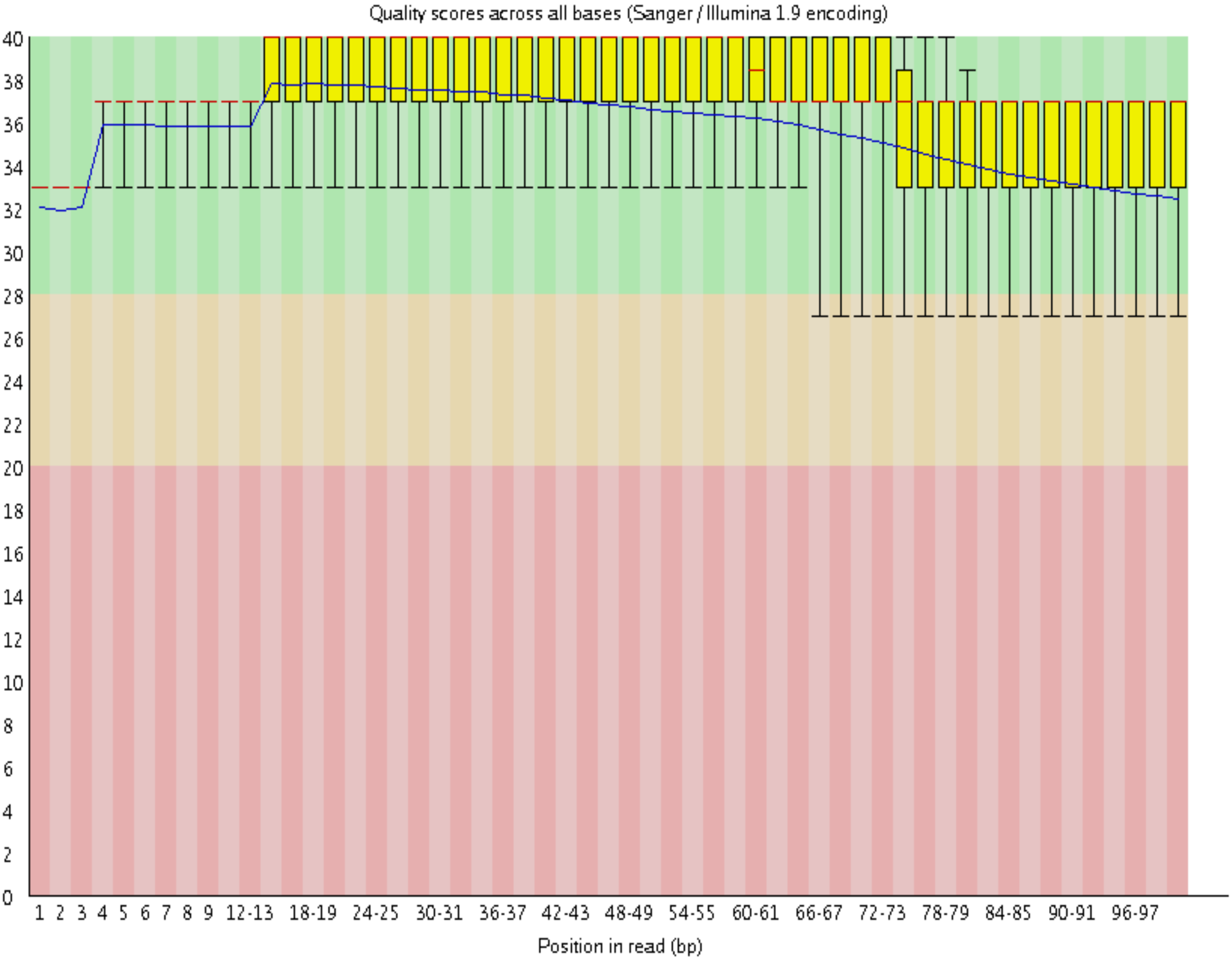

Per tile sequence quality

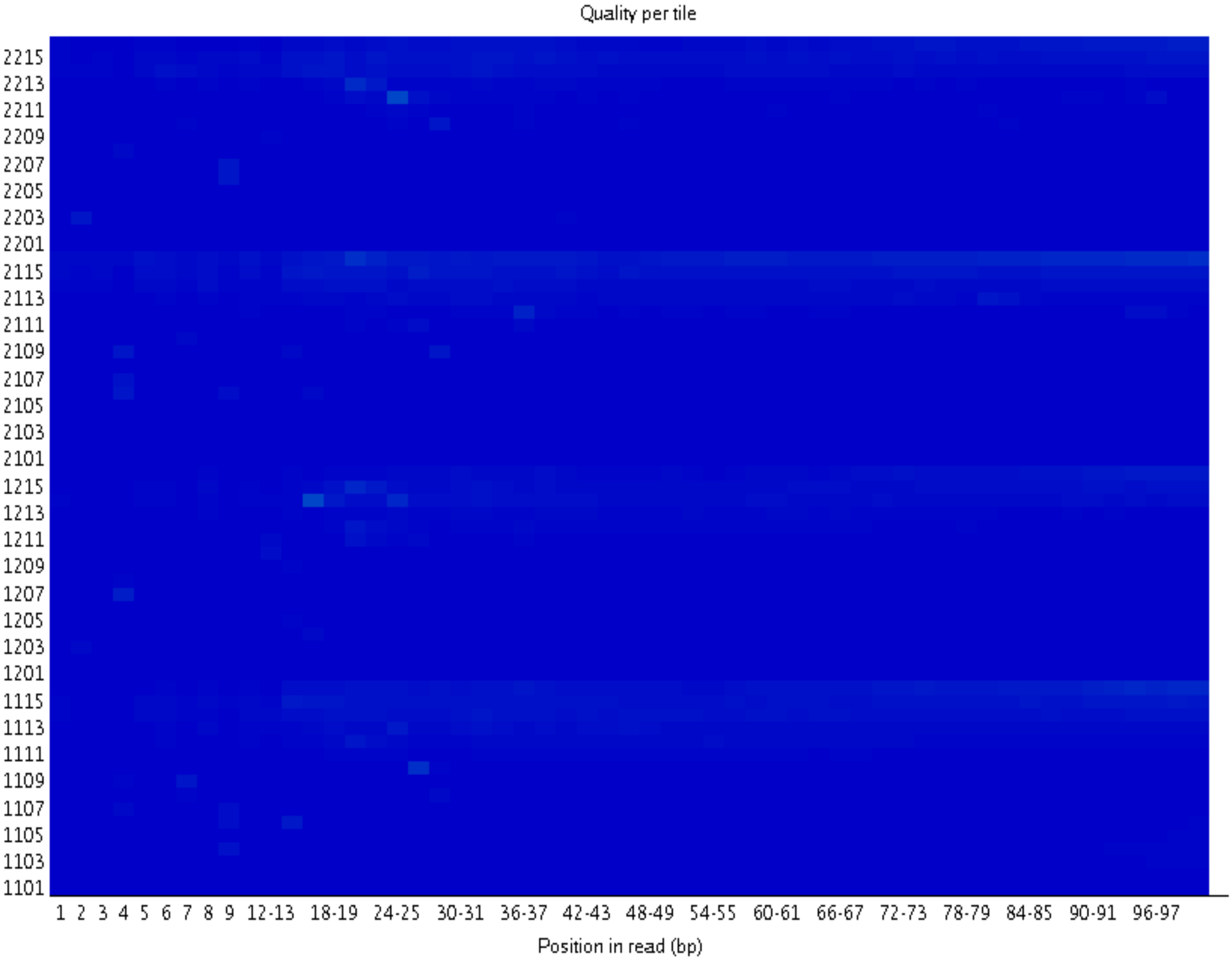

Per sequence quality scores

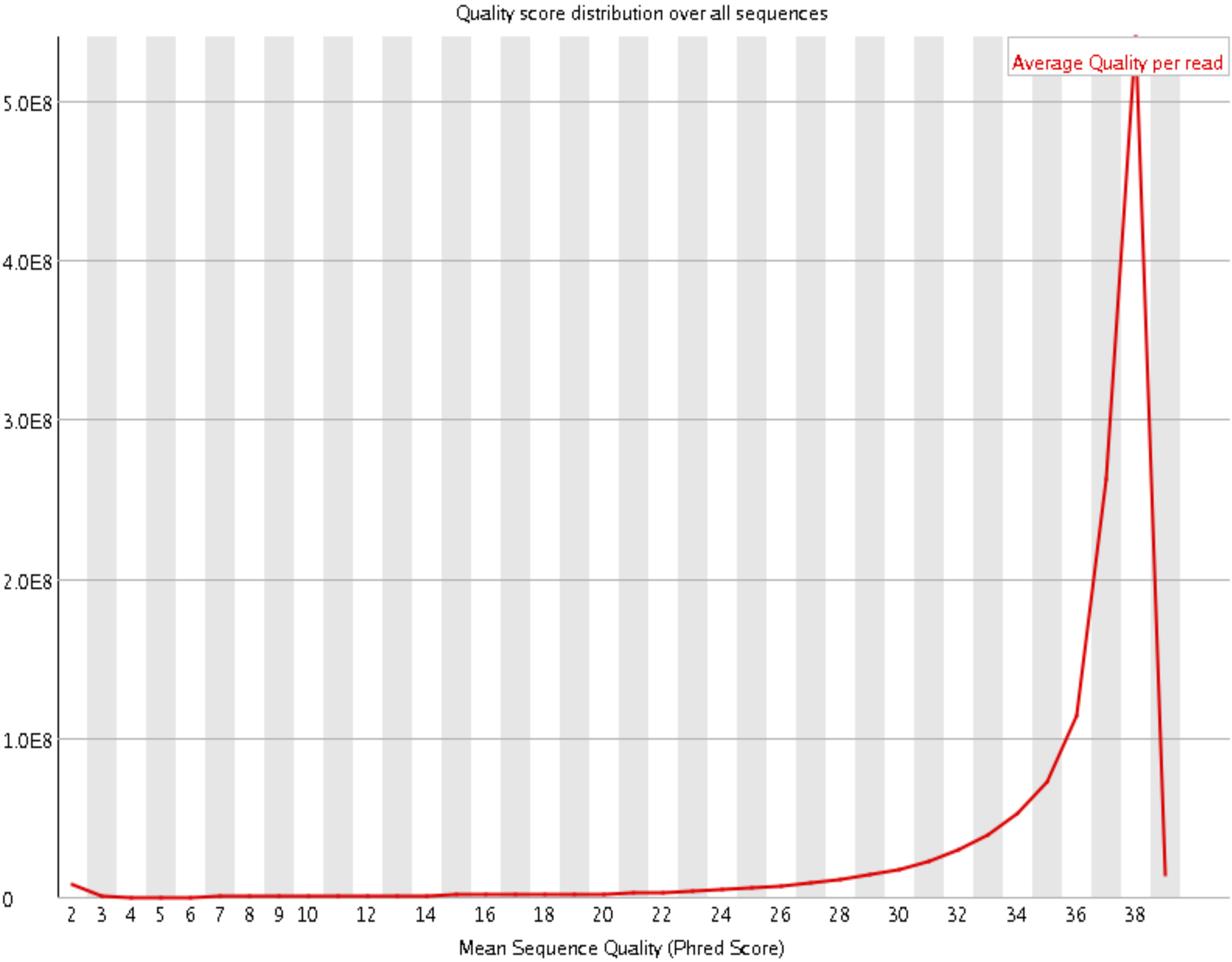

Per base sequence content

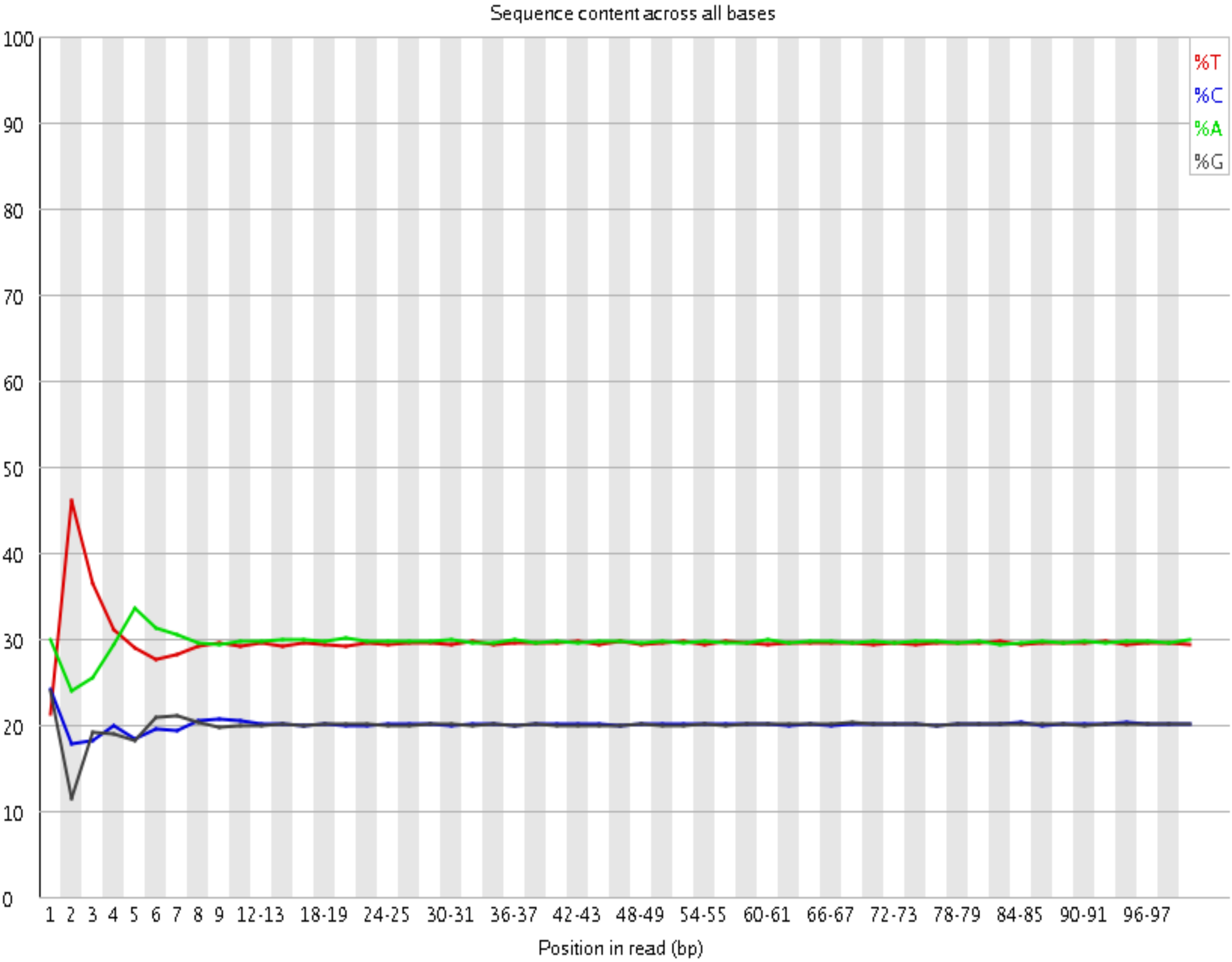

Per sequence GC content

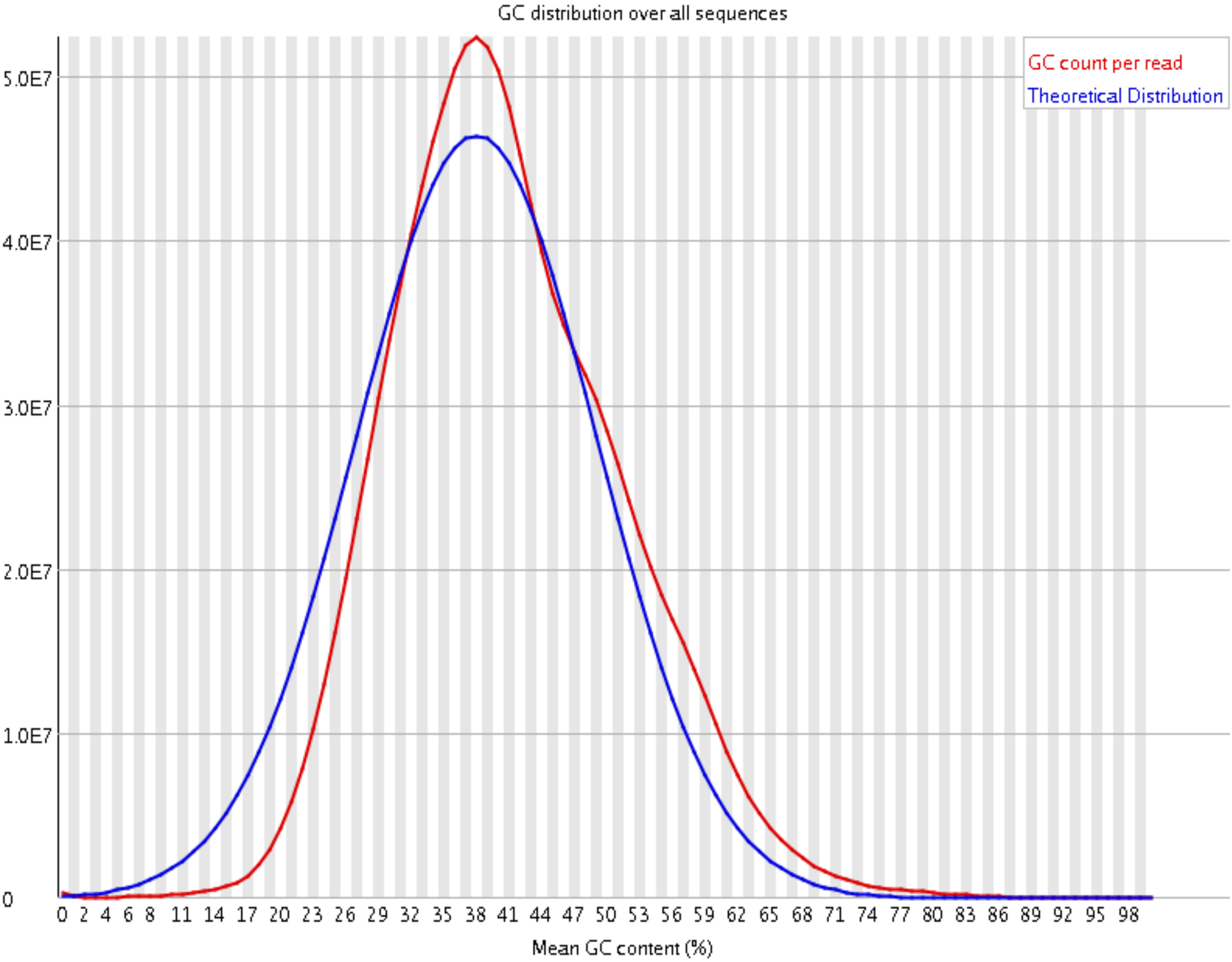

Per base N content

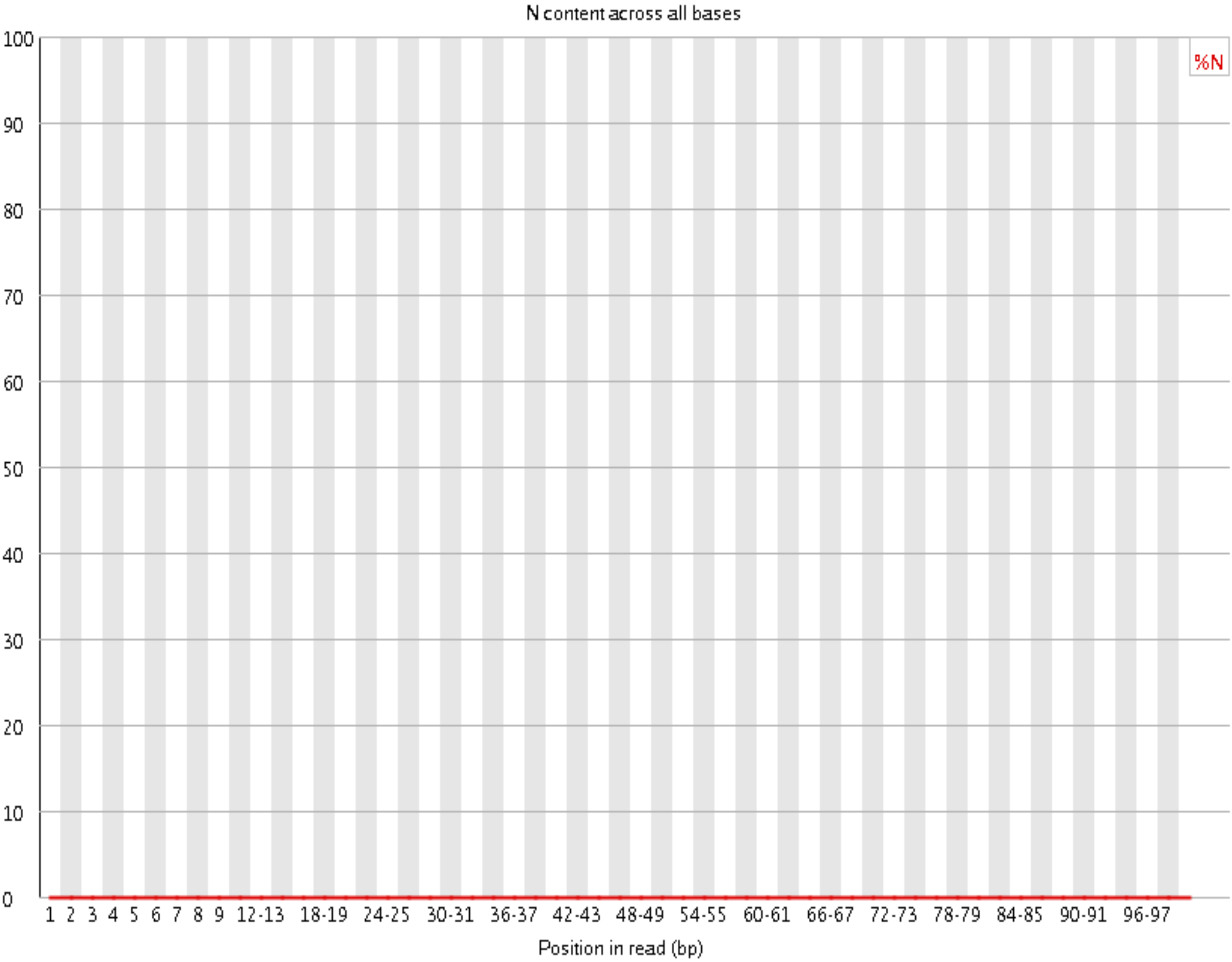

Sequence Length Distribution

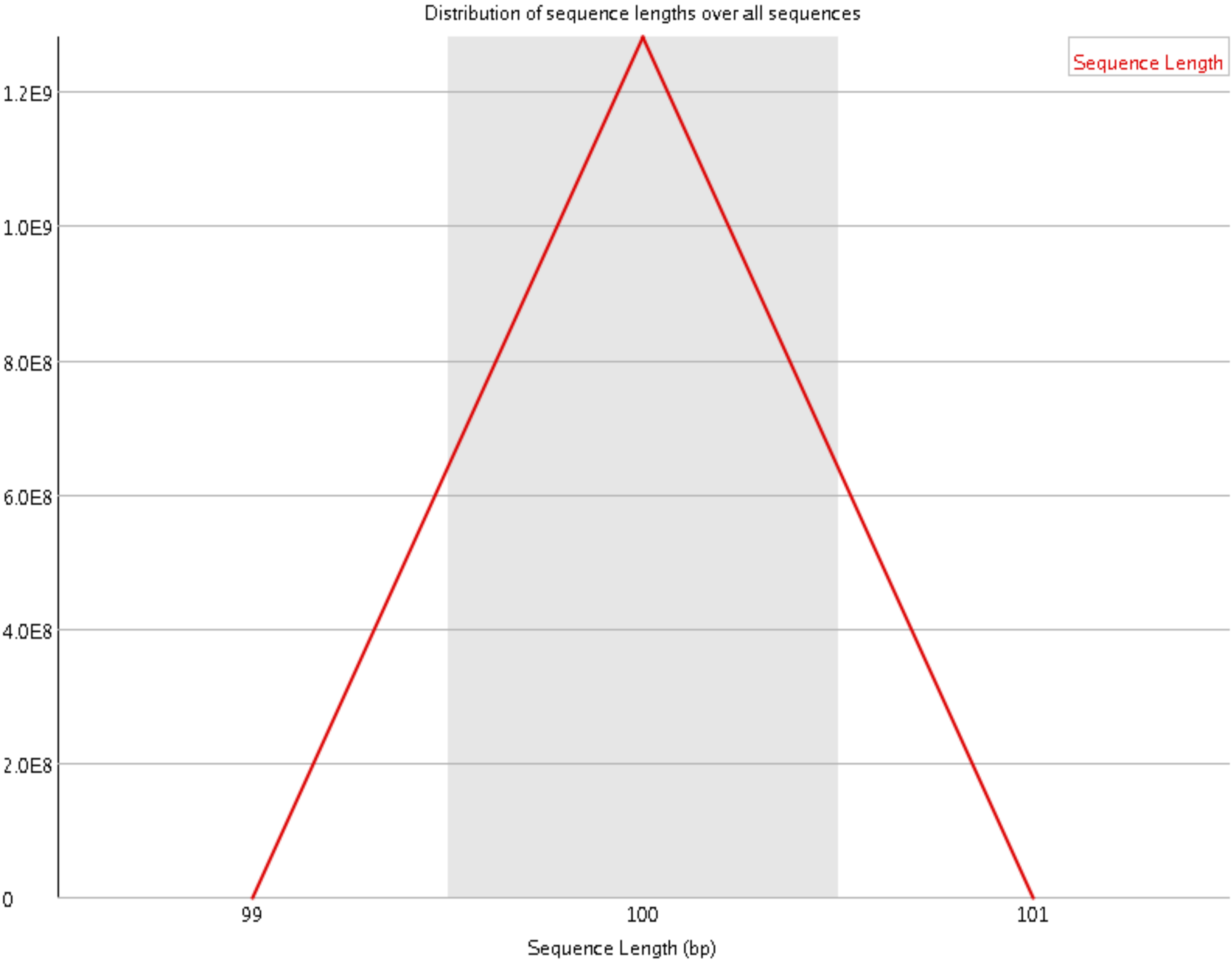

Sequence Duplication Levels

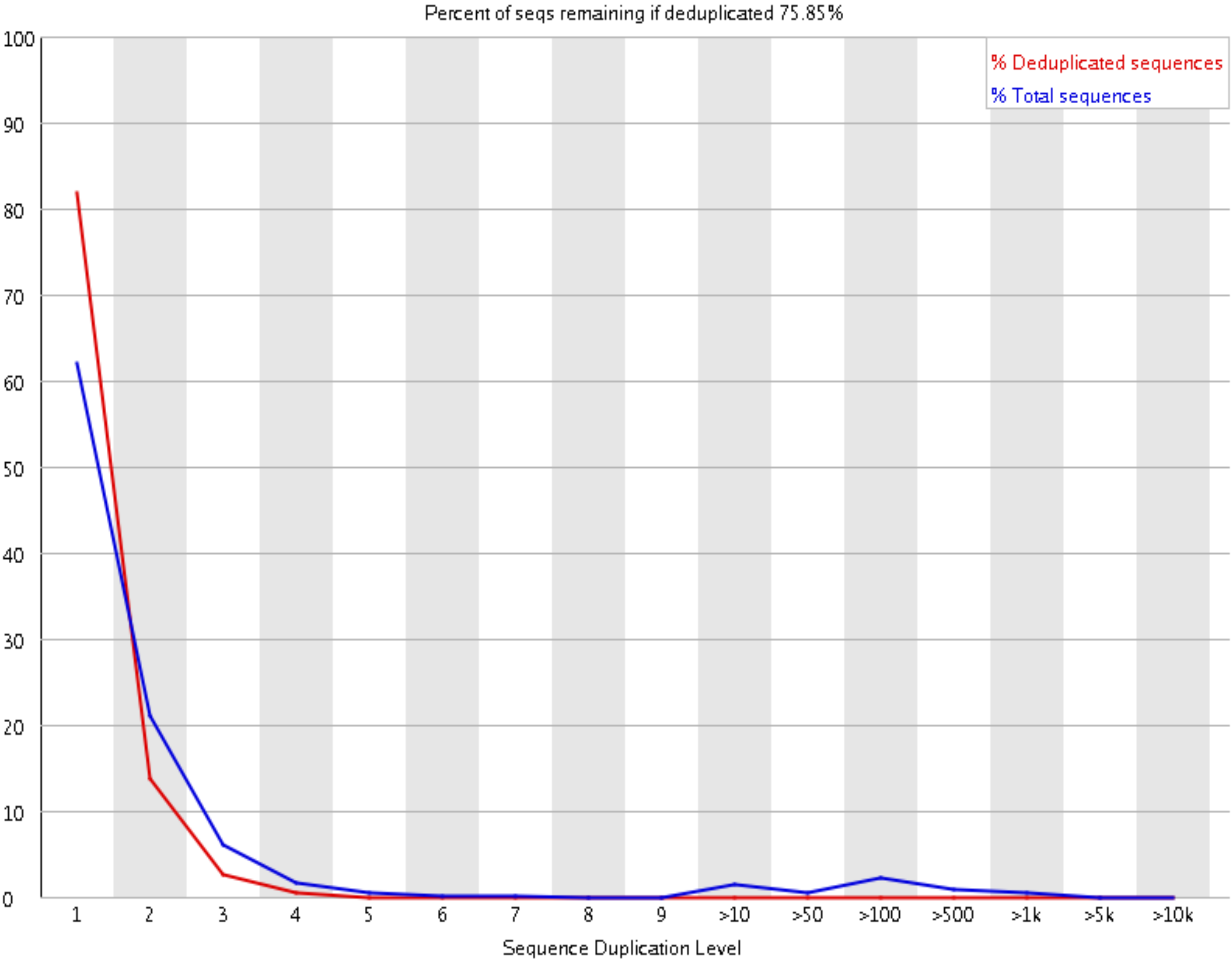

Overrepresented sequences

No overrepresented sequences

Adapter Content

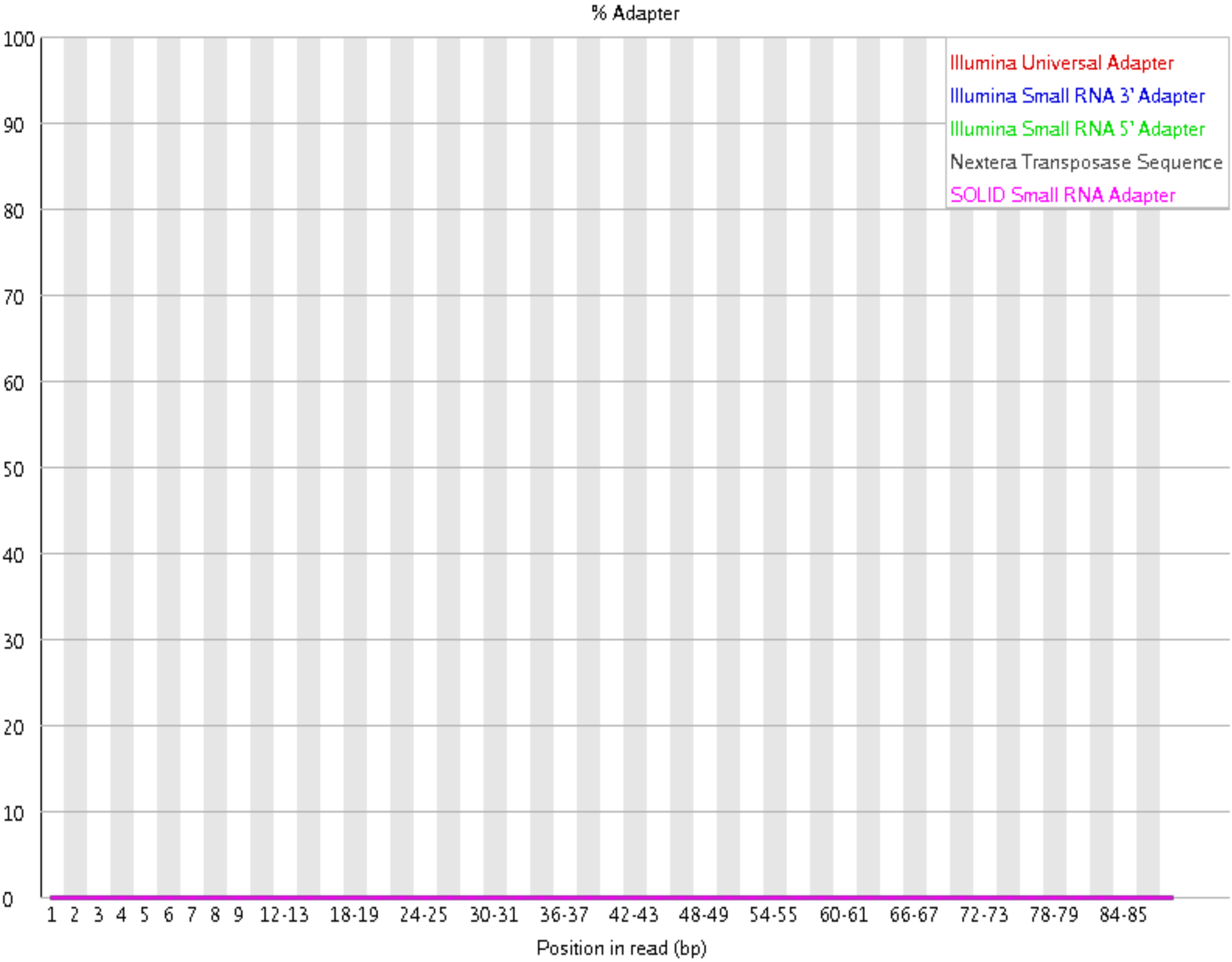

Produced by FastQC (version 0.11.7)

FastQC Report

Summary

- Basic Statistics
- Per base sequence quality
- Per tile sequence quality
- Per sequence quality scores
- Per base sequence content
- Per sequence GC content
- Per base N content
- Sequence Length Distribution
- Sequence Duplication Levels
- Overrepresented sequences
- Adapter Content

Basic Statistics

Measure	Value
Filename	stdin
File type	Conventional base calls
Encoding	Sanger / Illumina 1.9
Total Sequences	553277870
Sequences flagged as poor quality	0
Sequence length	35-159
%GC	41

Per base sequence quality

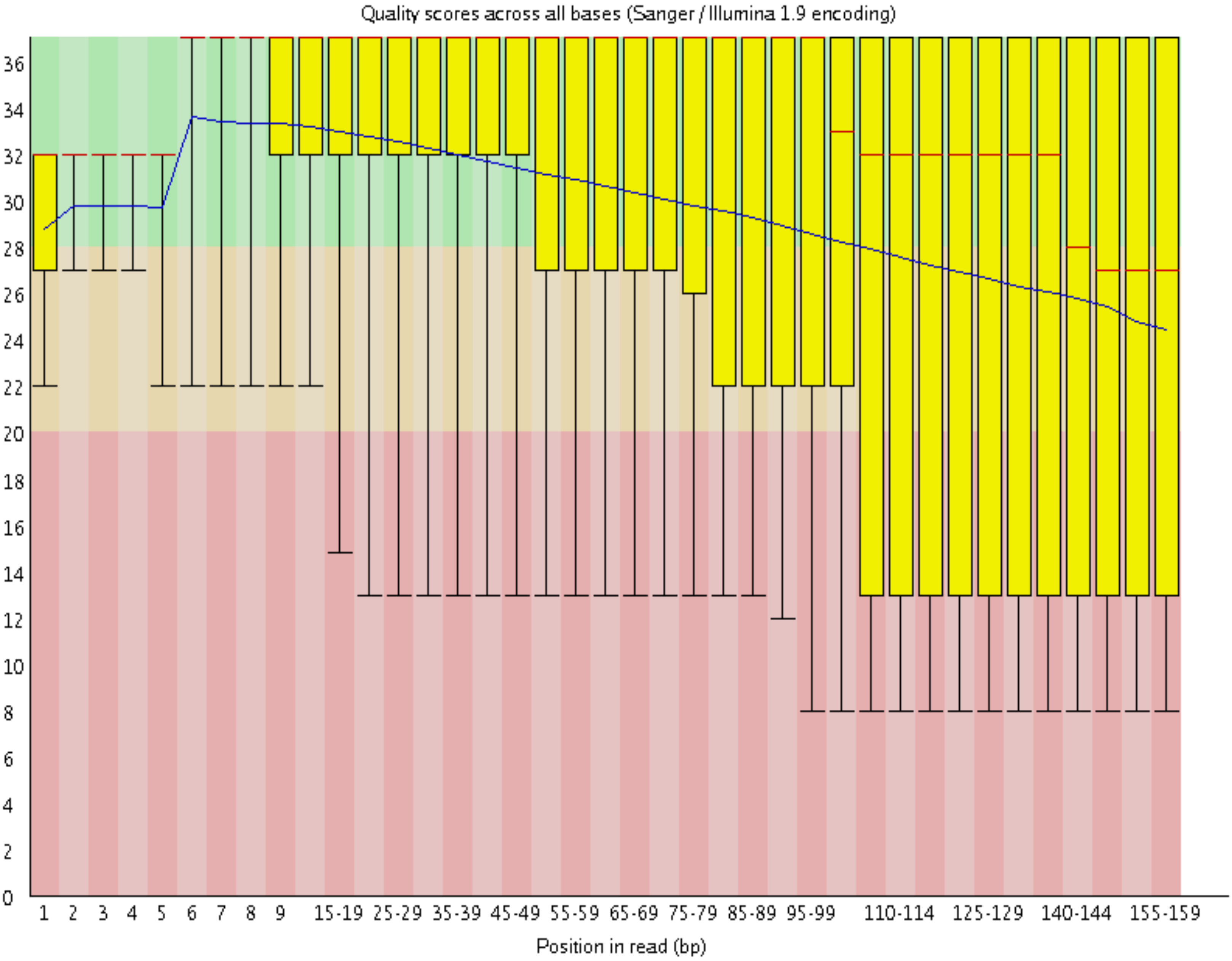

Per tile sequence quality

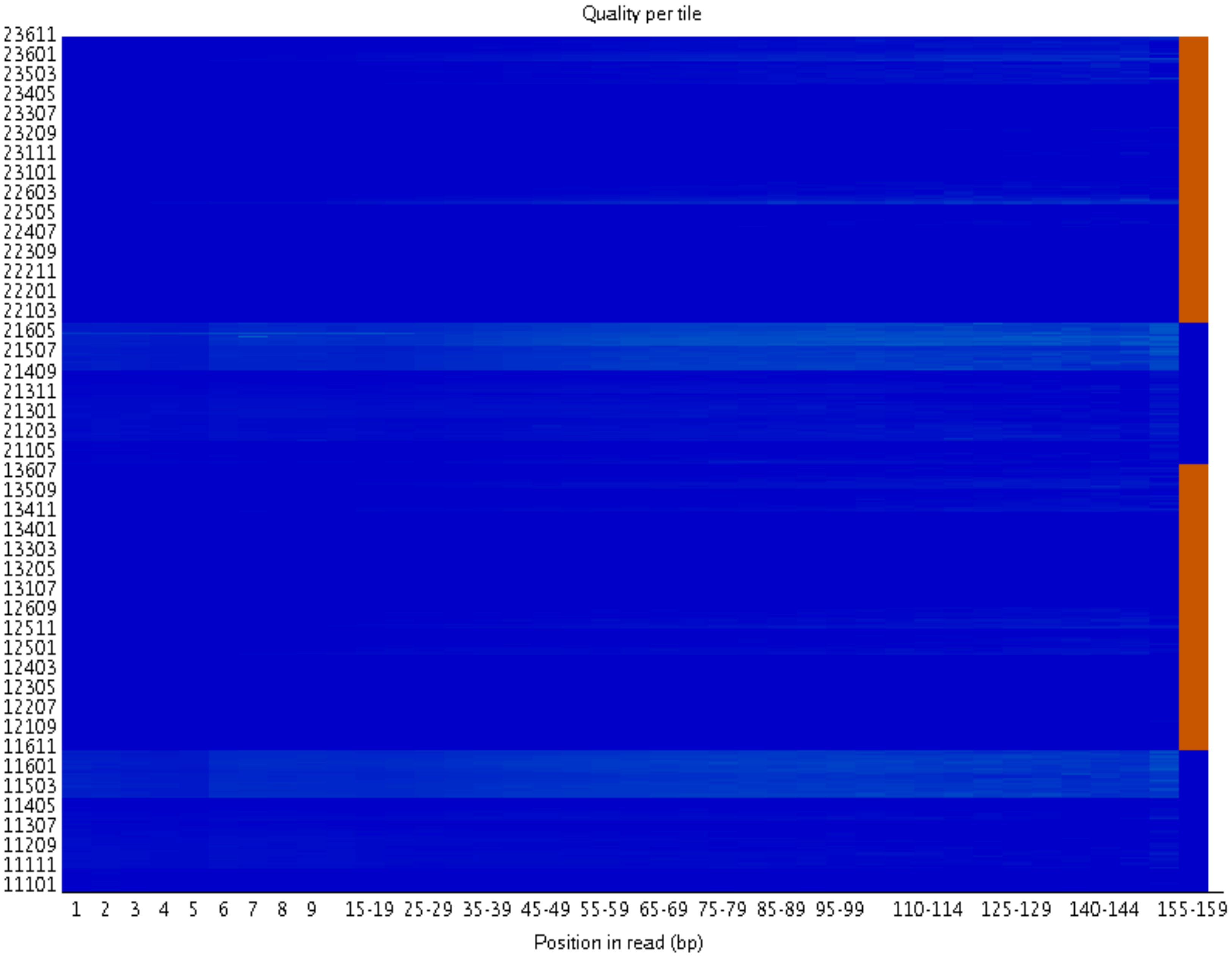

Per sequence quality scores

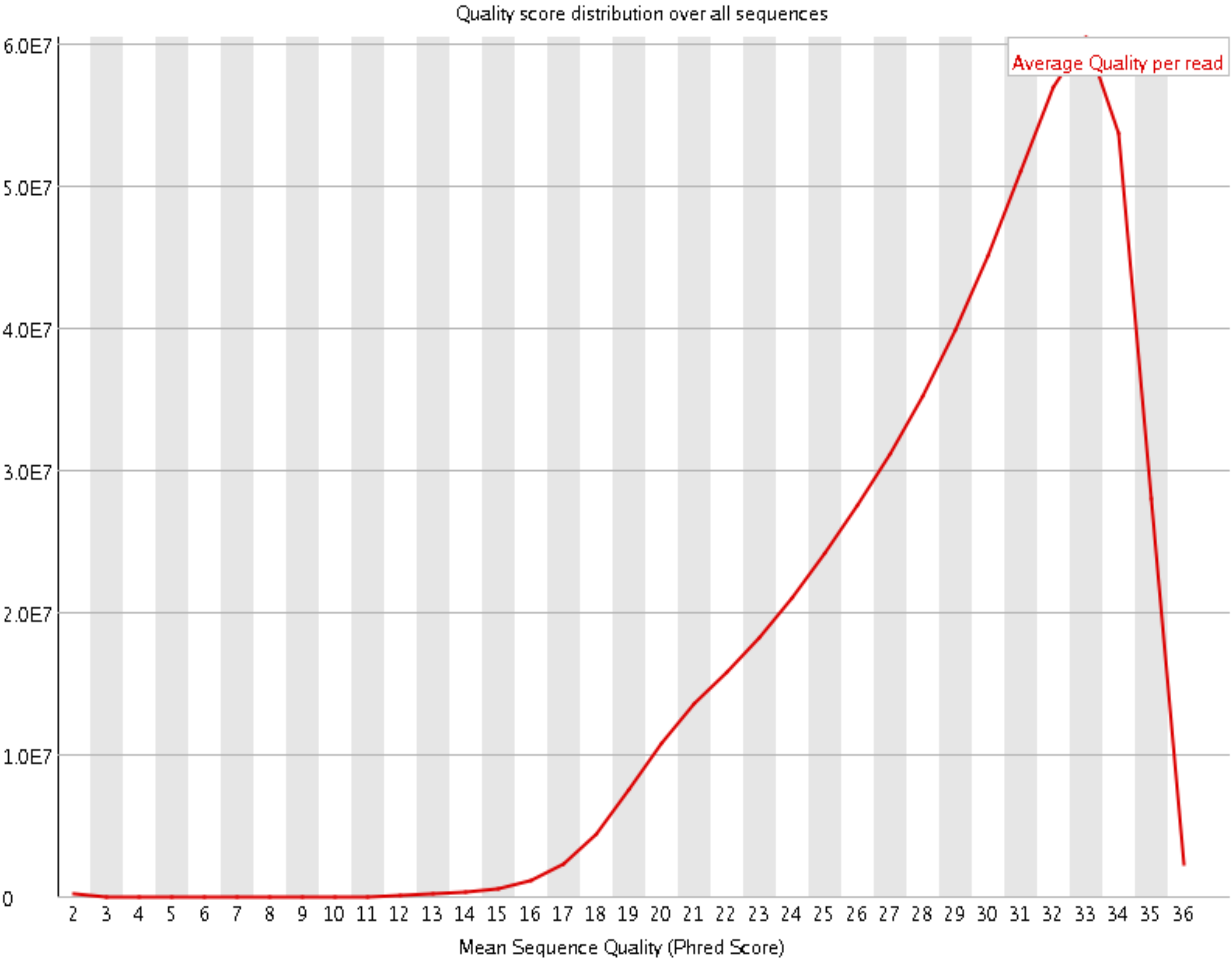

Per base sequence content

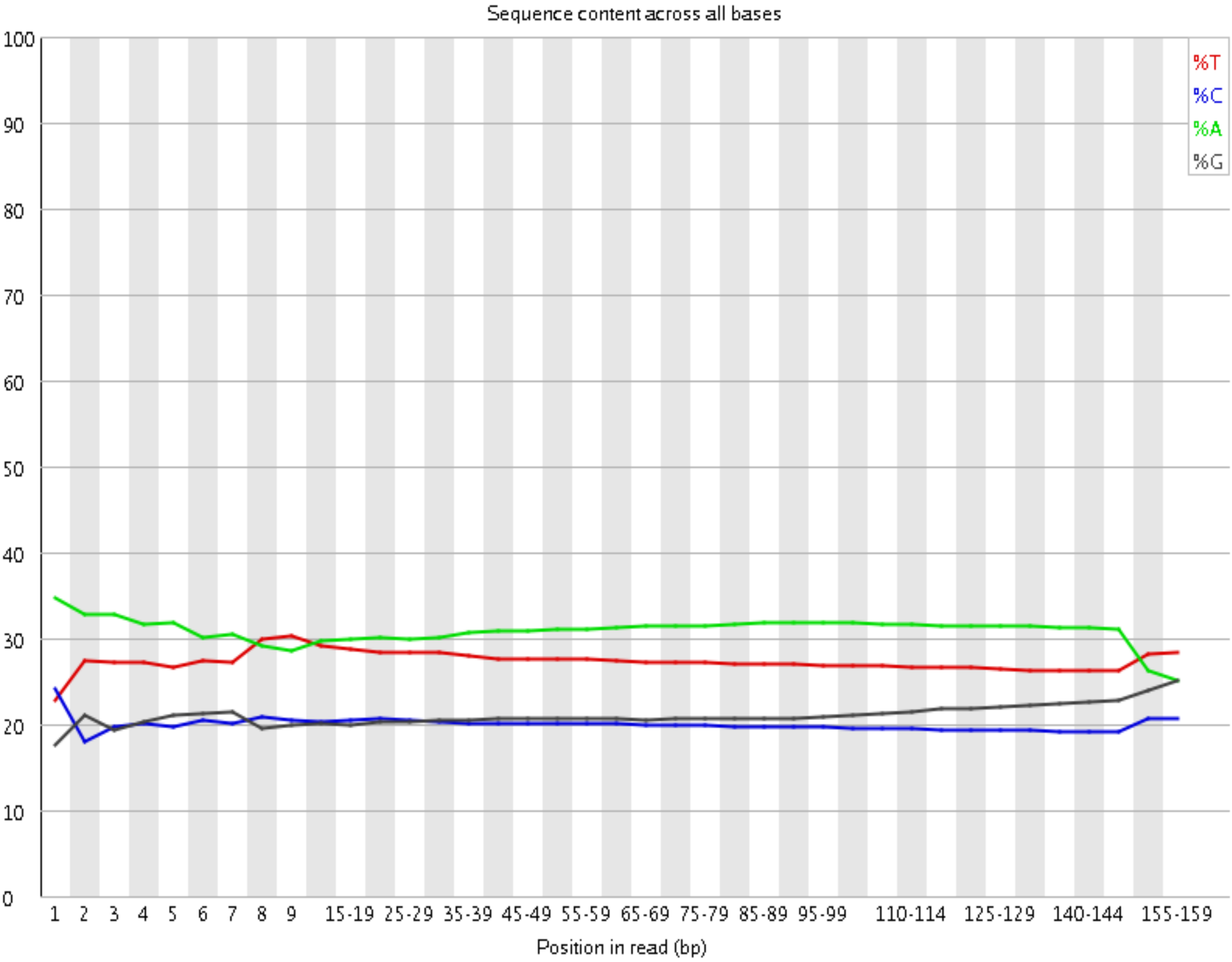

Per sequence GC content

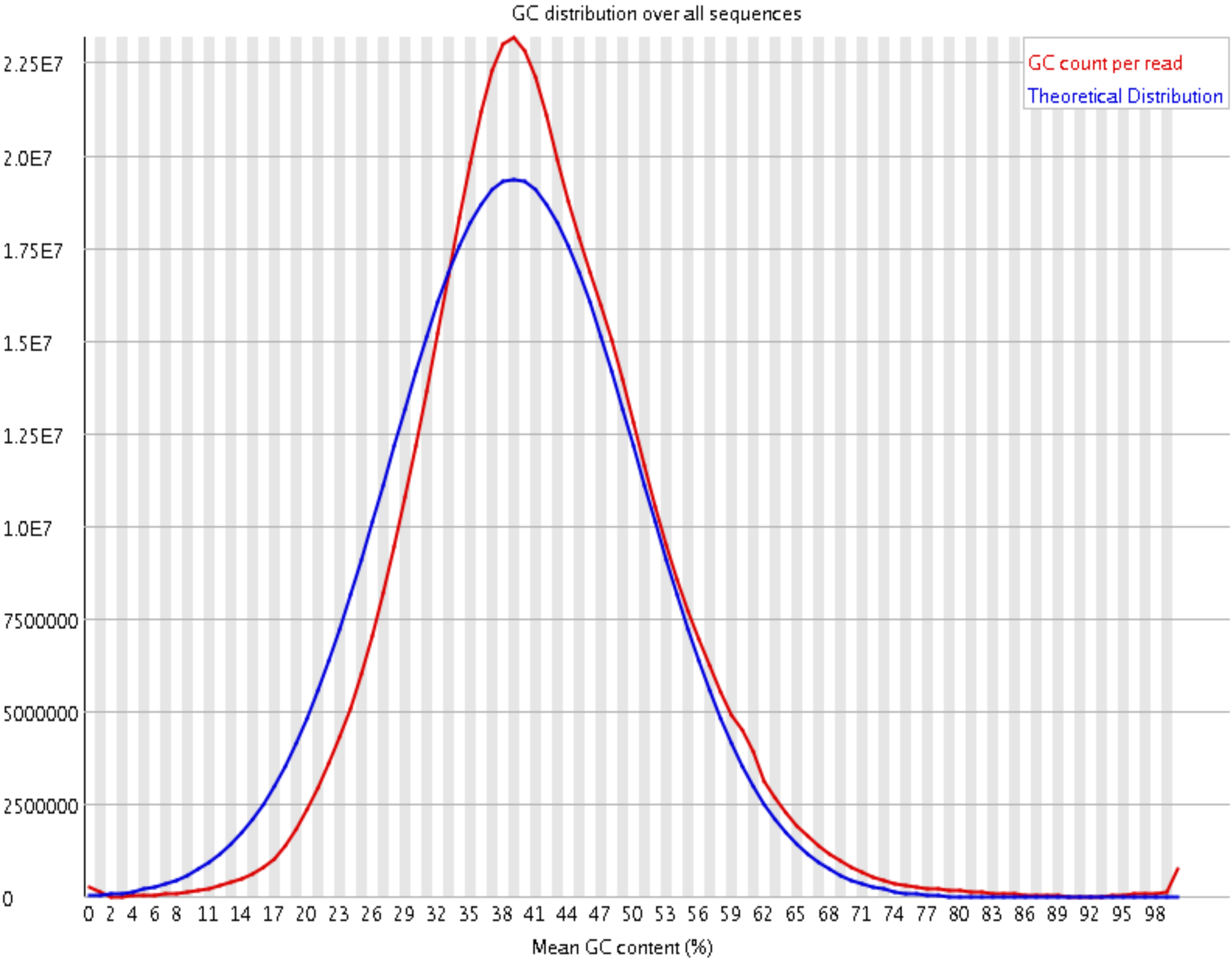

Per base N content

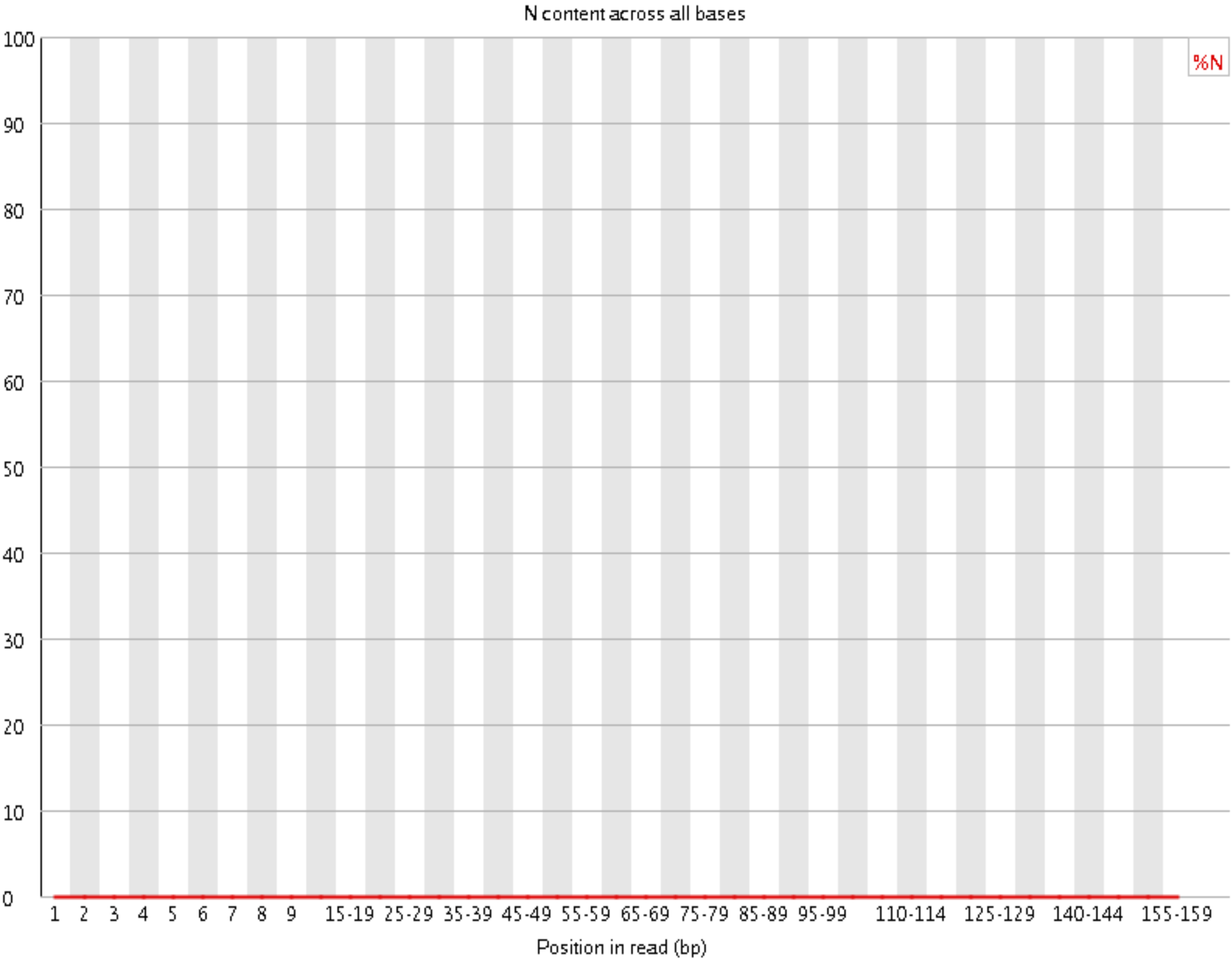

Sequence Length Distribution

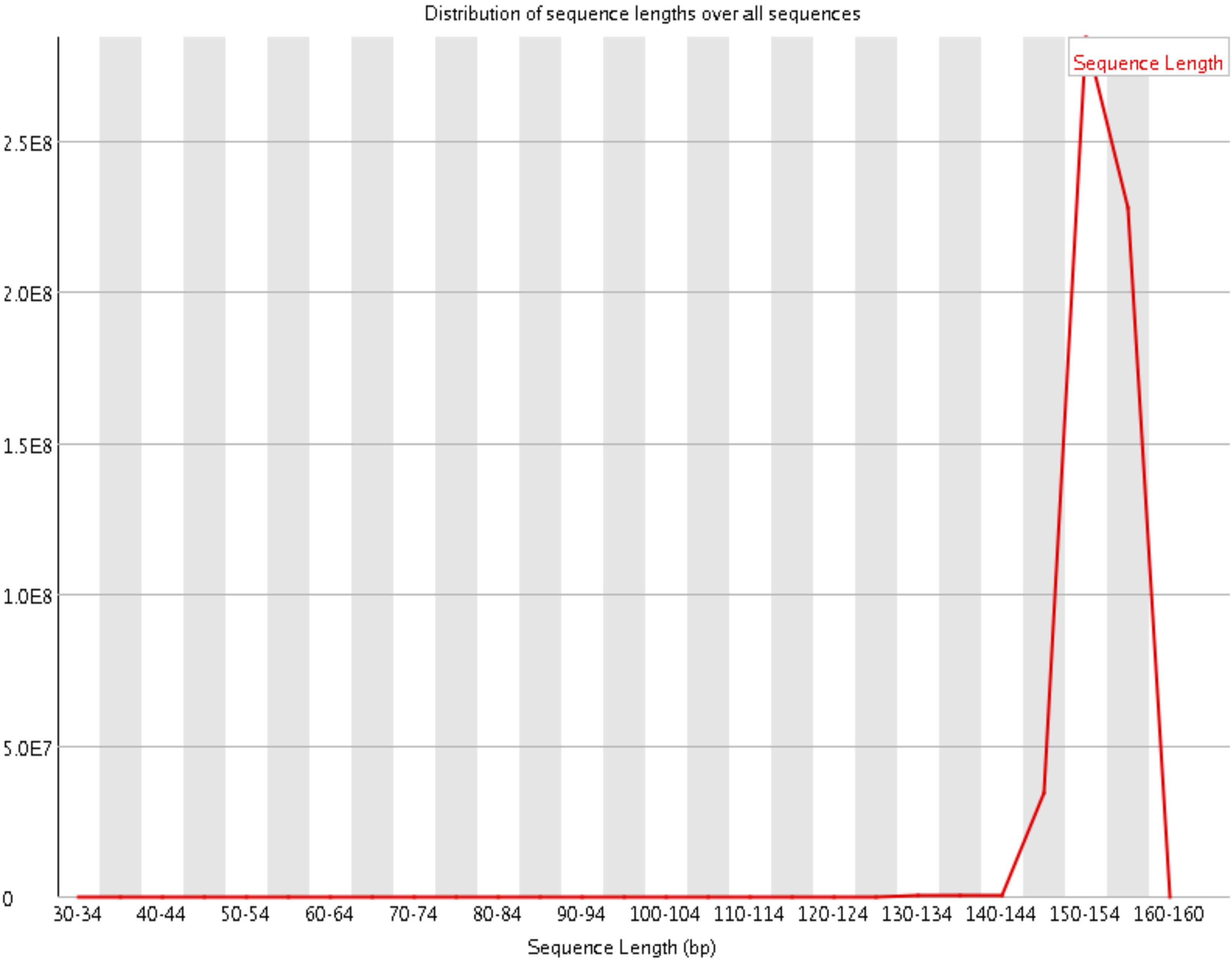

Sequence Duplication Levels

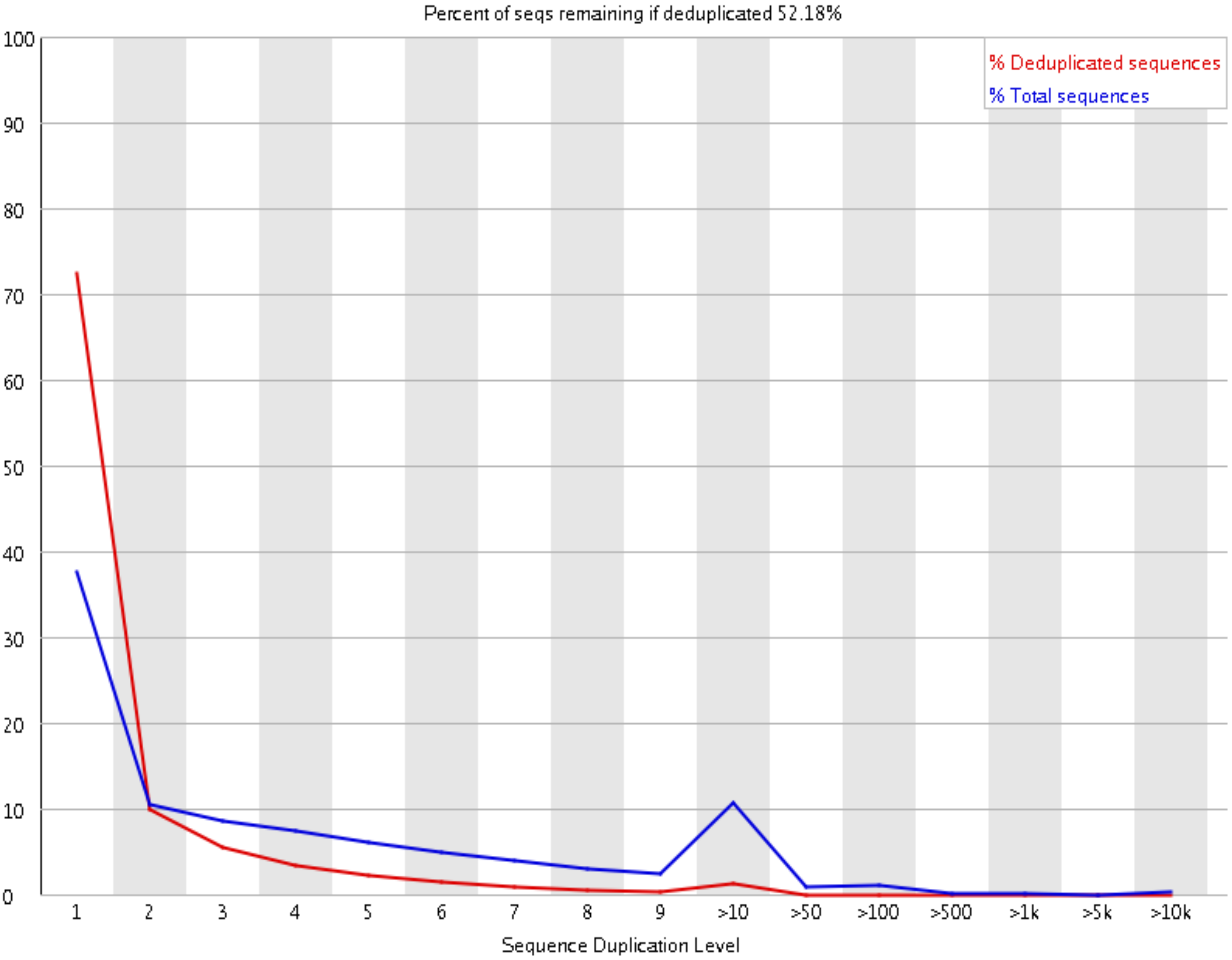

Overrepresented sequences

Sequence	Count	Percentage	Possible Source
GGGGGGGGGGGGGGGGGGGGGGGGGGGGGGGGGGGGGGGGGGGGGGGGGG	1464007	0.2646061010898556	No Hit

Adapter Content

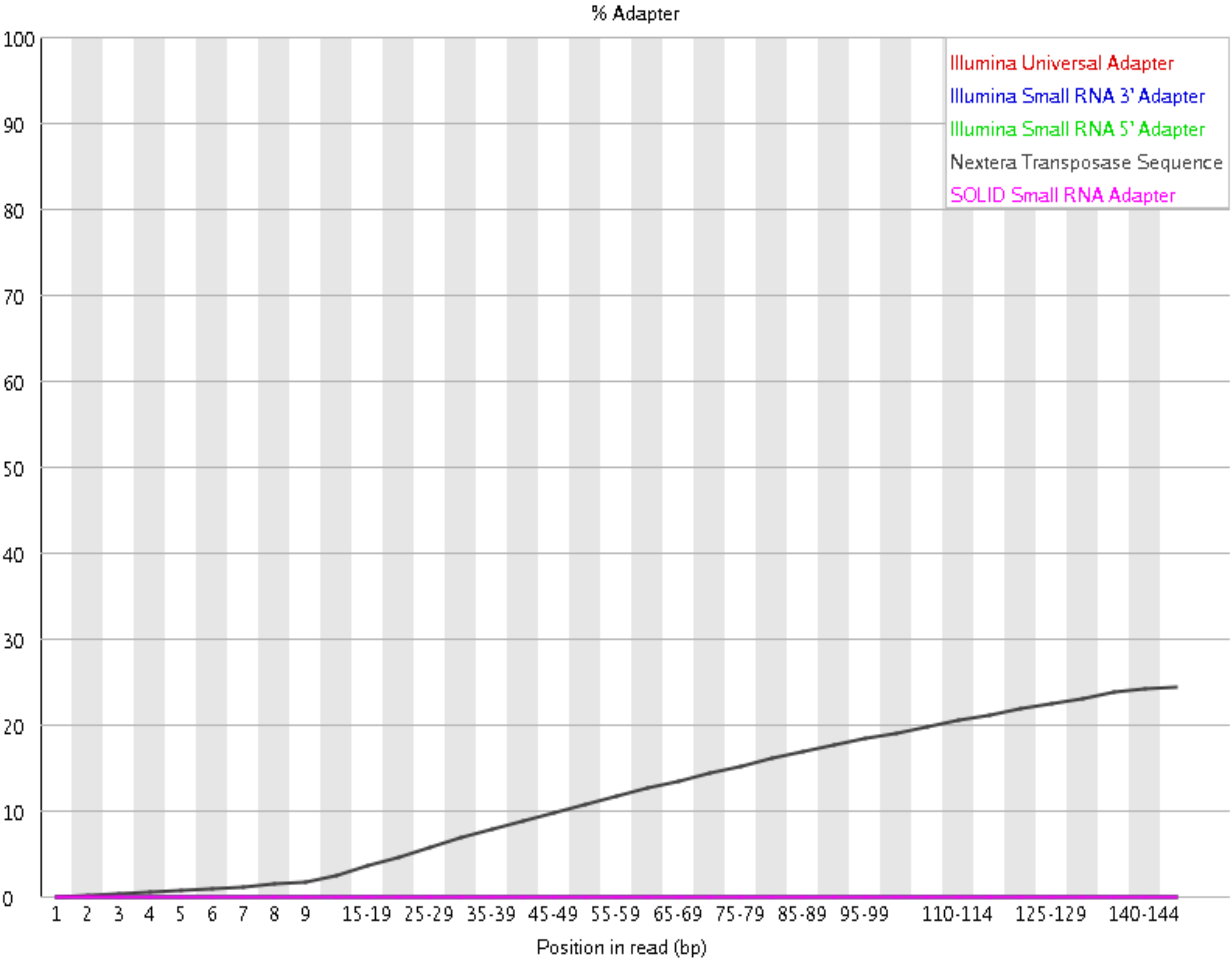

Produced by FastQC (version 0.11.7)

FastQC Report

Sat 31 Mar 2018
stdin

Summary

- Basic Statistics
- Per base sequence quality
- Per tile sequence quality
- Per sequence quality scores
- Per base sequence content
- Per sequence GC content
- Per base N content
- Sequence Length Distribution
- Sequence Duplication Levels
- Overrepresented sequences
- Adapter Content

Basic Statistics

Measure	Value
Filename	stdin
File type	Conventional base calls
Encoding	Sanger / Illumina 1.9
Total Sequences	1280576580
Sequences flagged as poor quality	0
Sequence length	100
%GC	40

Per base sequence quality

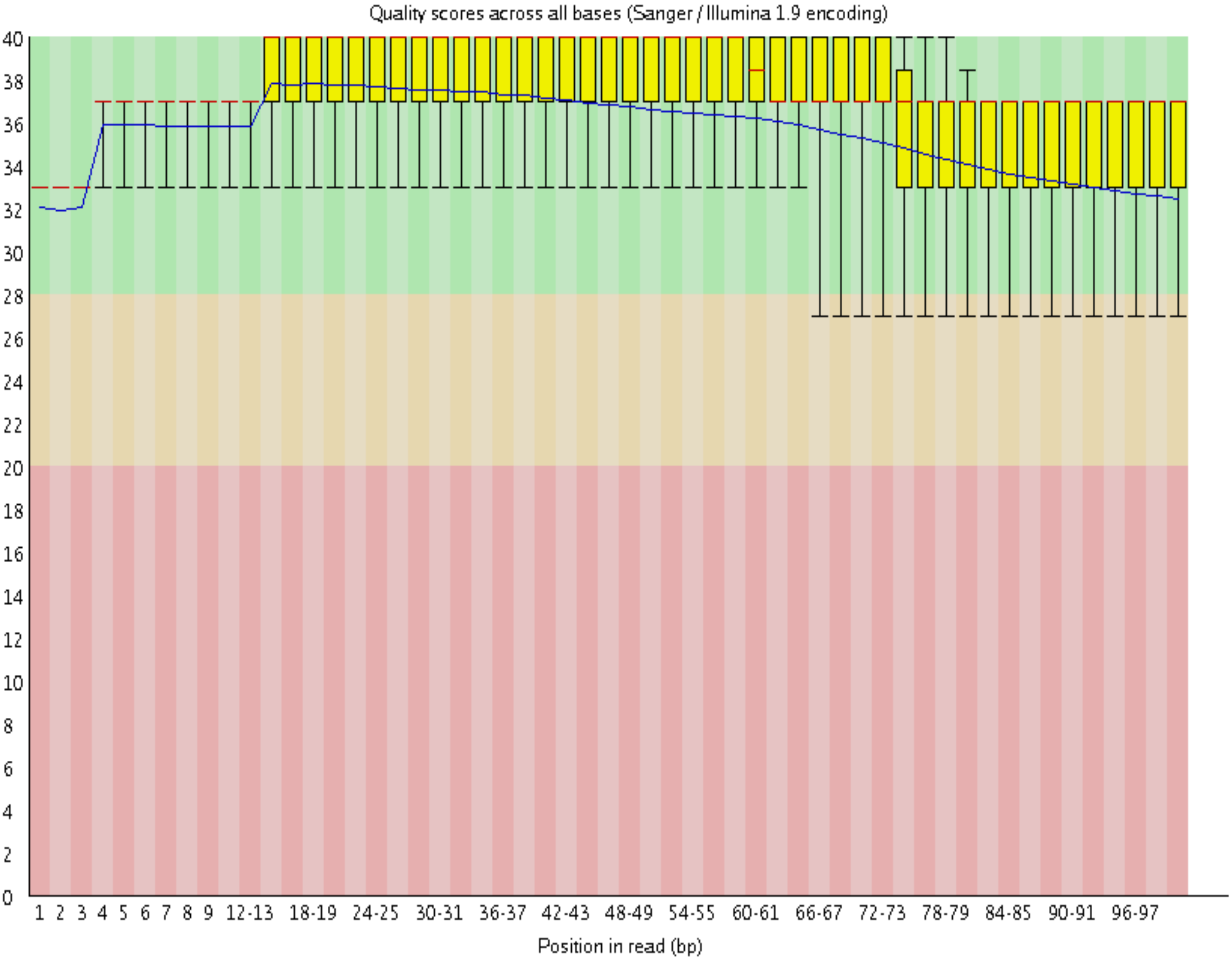

Per tile sequence quality

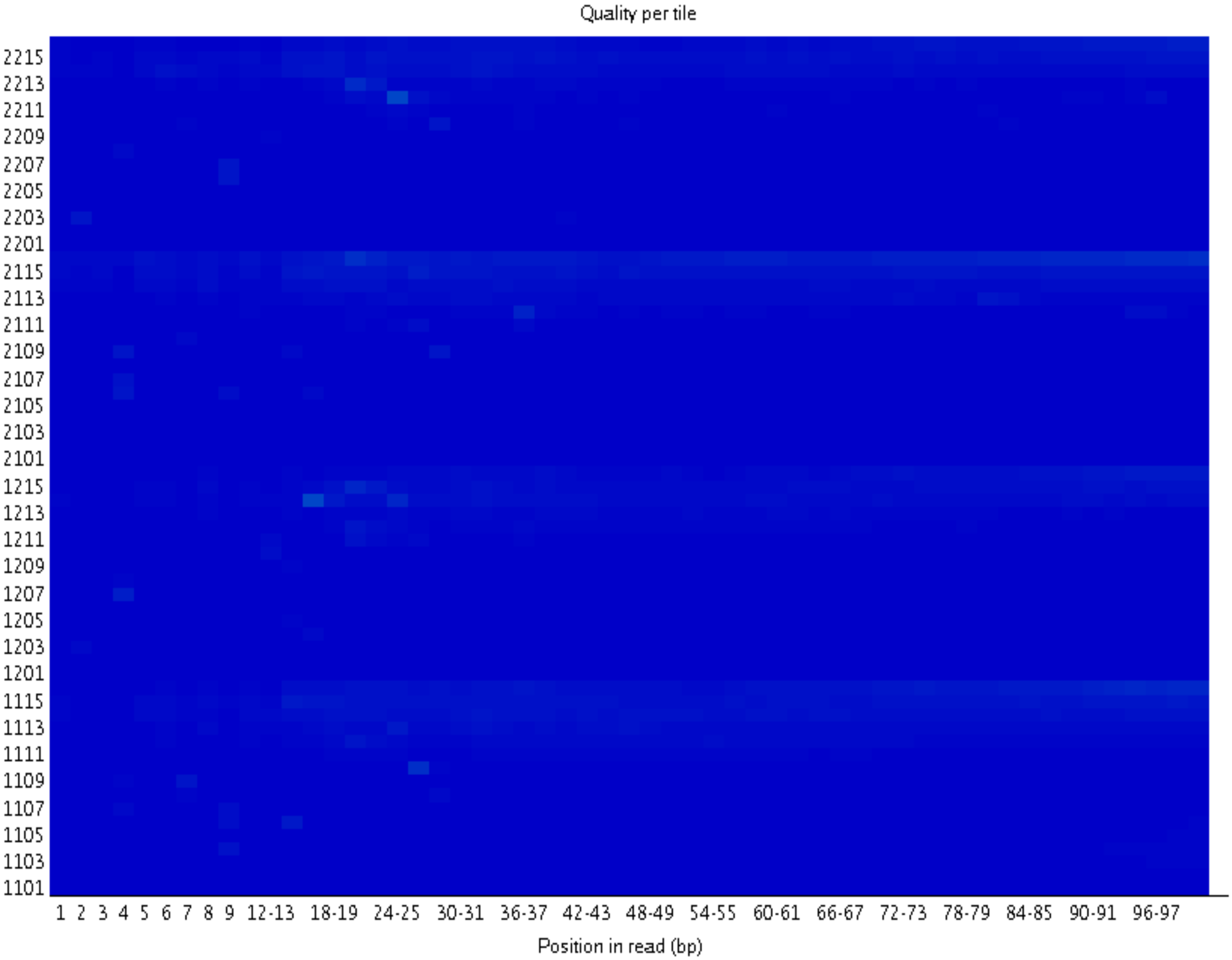

Per sequence quality scores

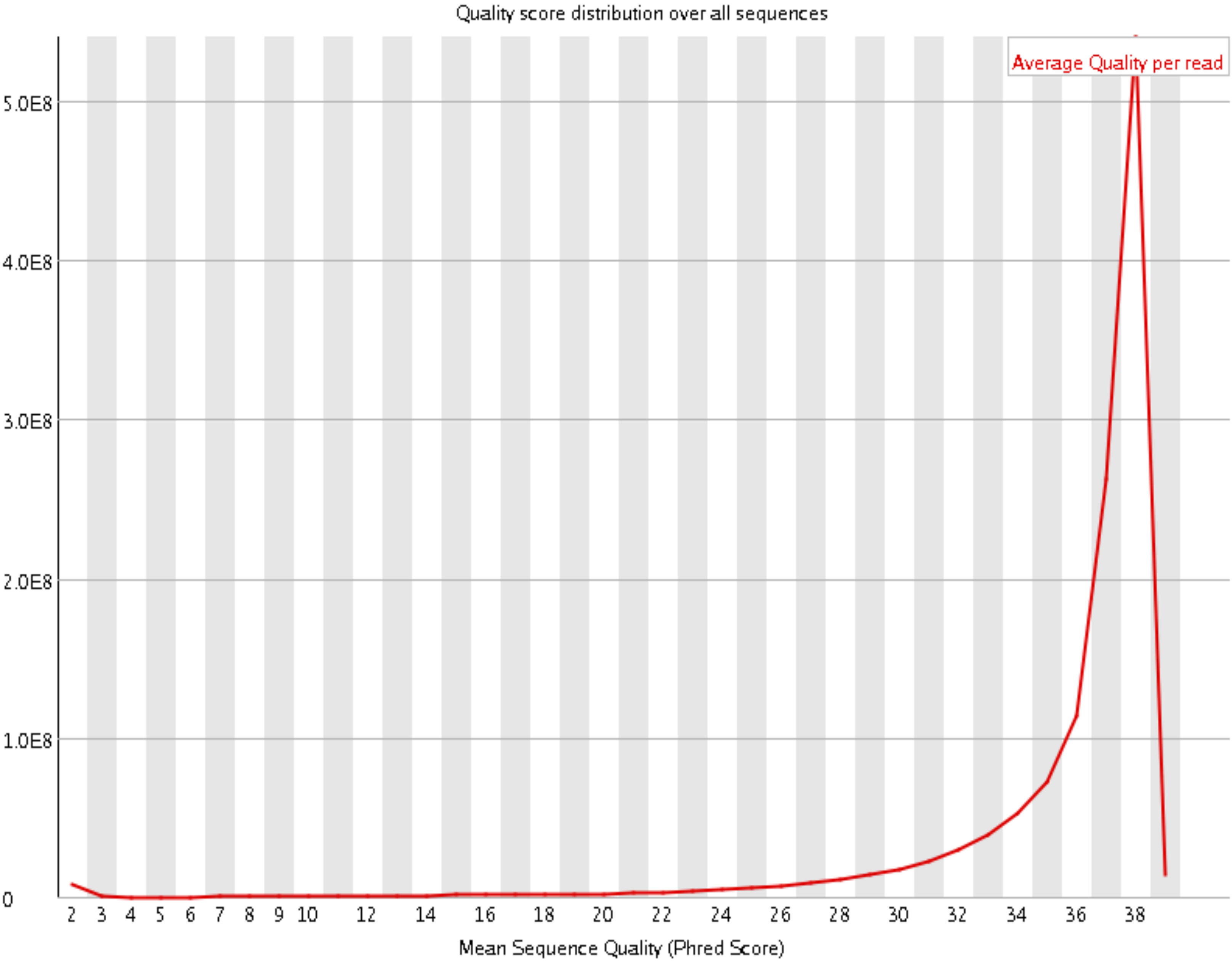

Per base sequence content

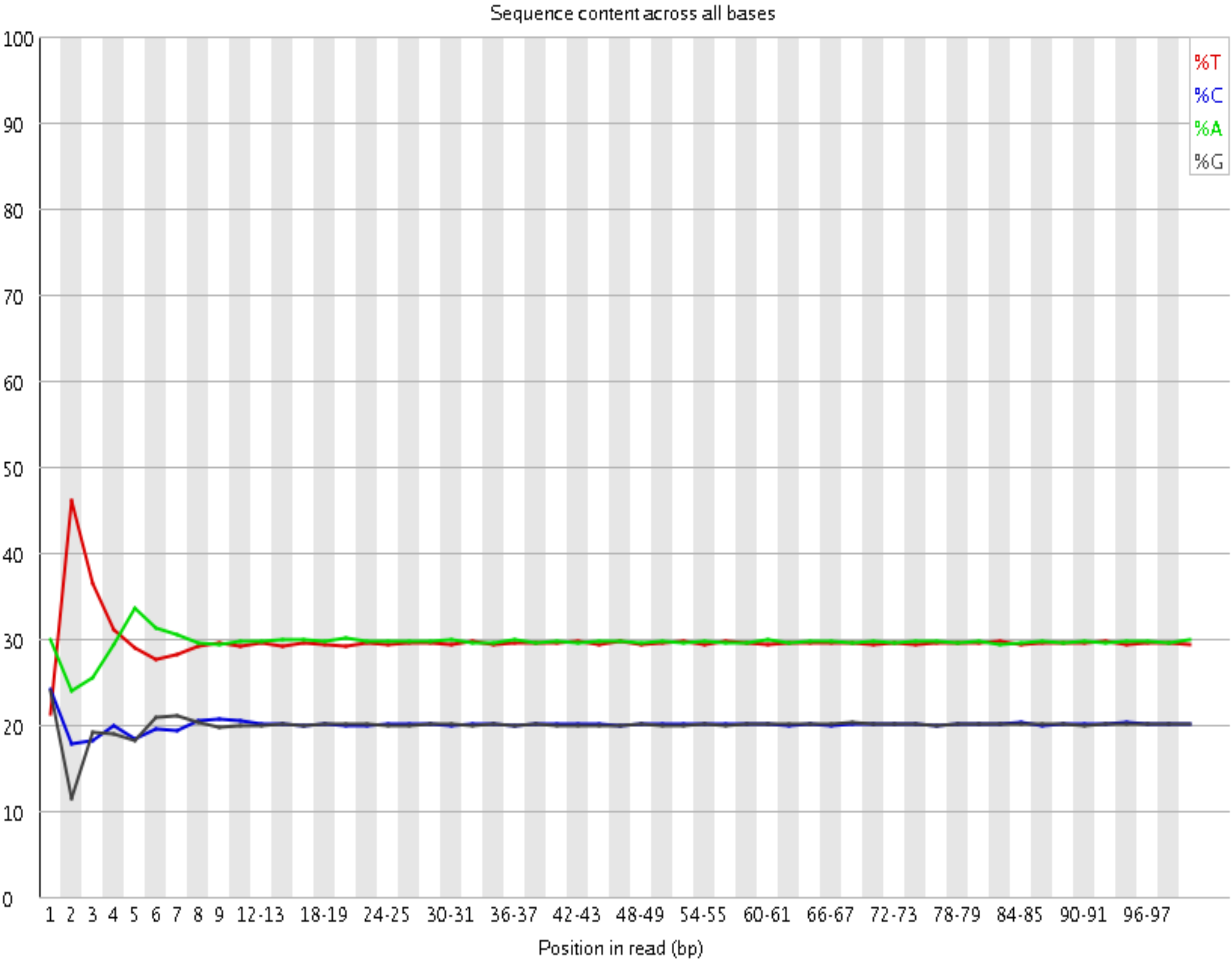

Per sequence GC content

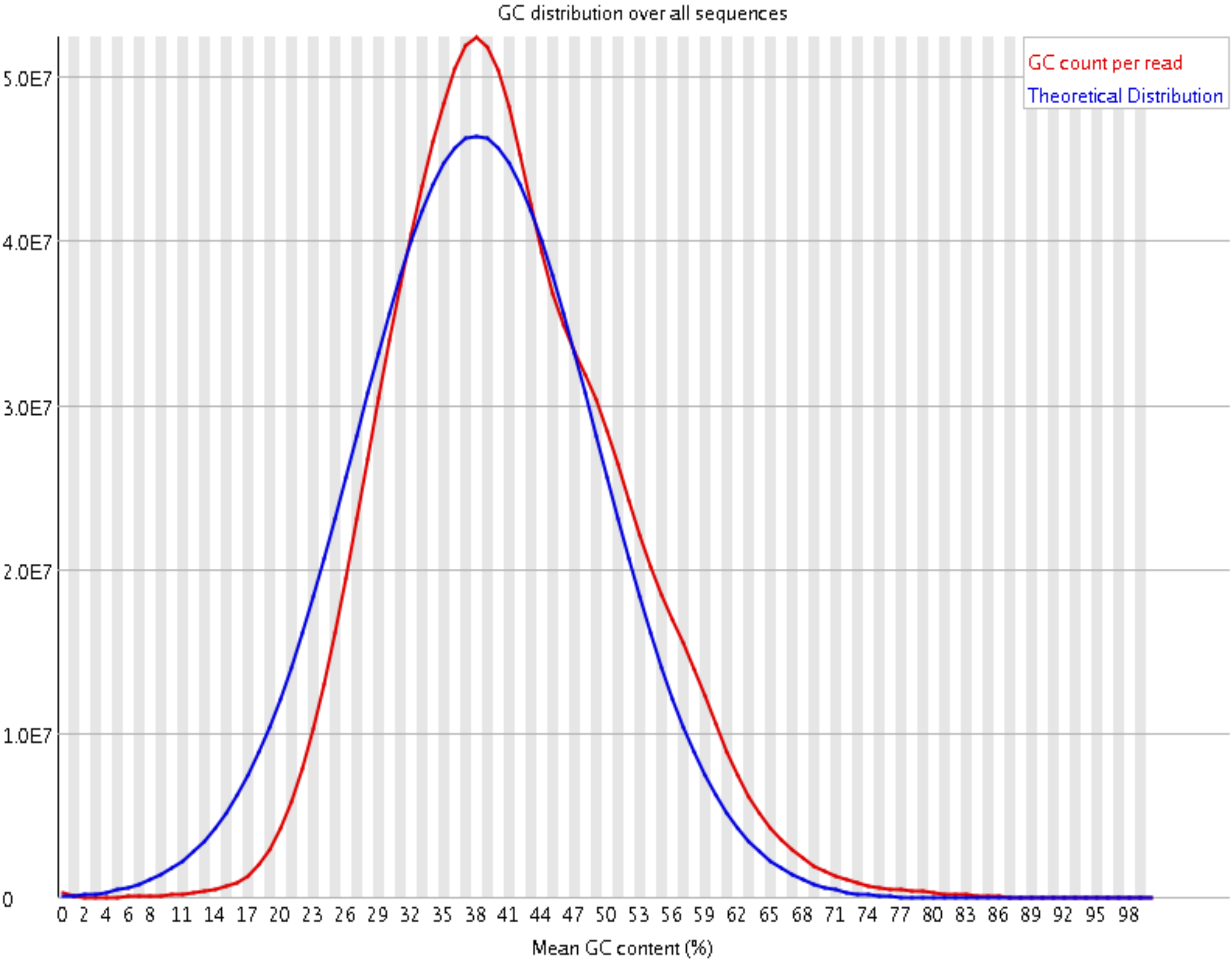

Per base N content

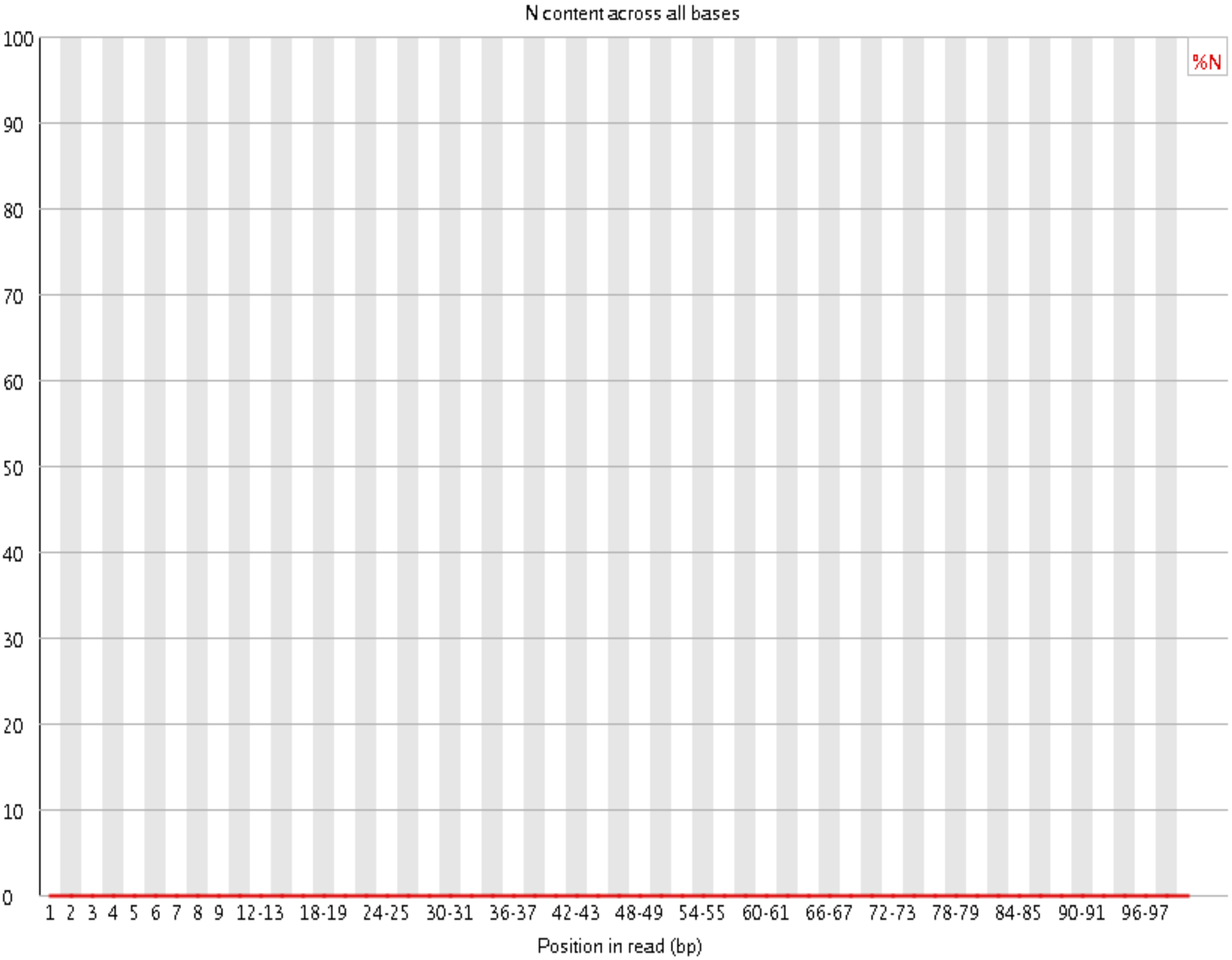

Sequence Length Distribution

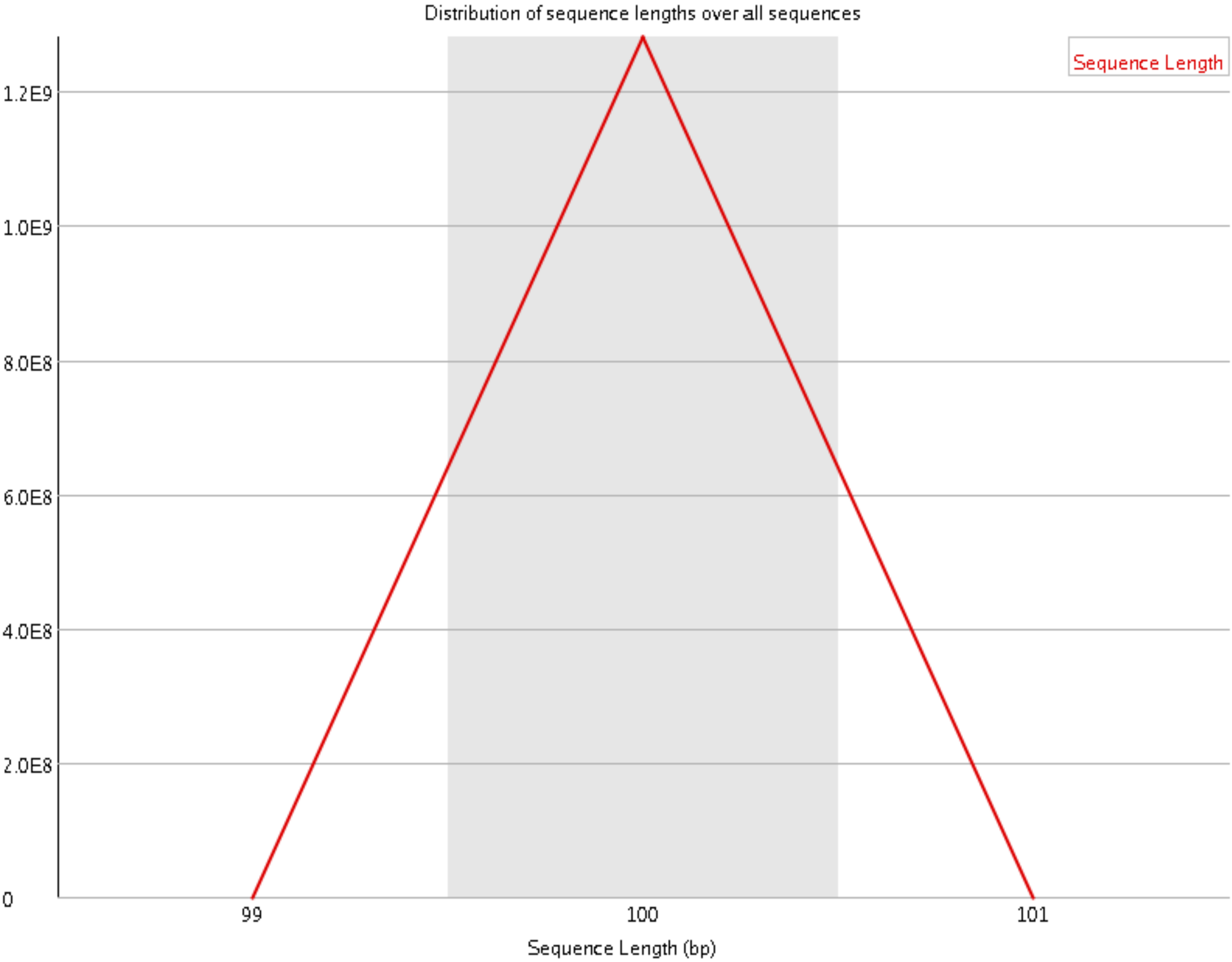

Sequence Duplication Levels

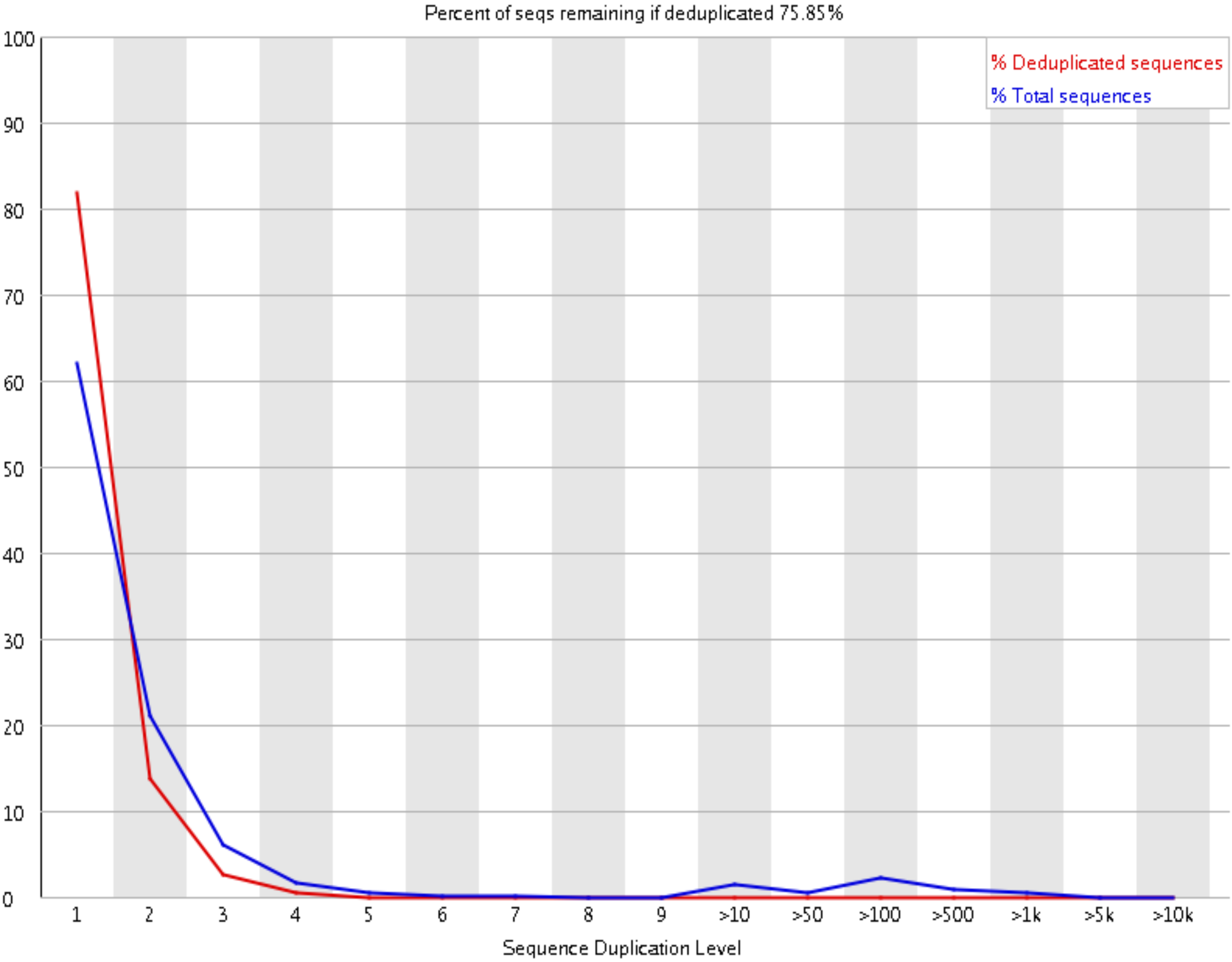

Overrepresented sequences

No overrepresented sequences

Adapter Content

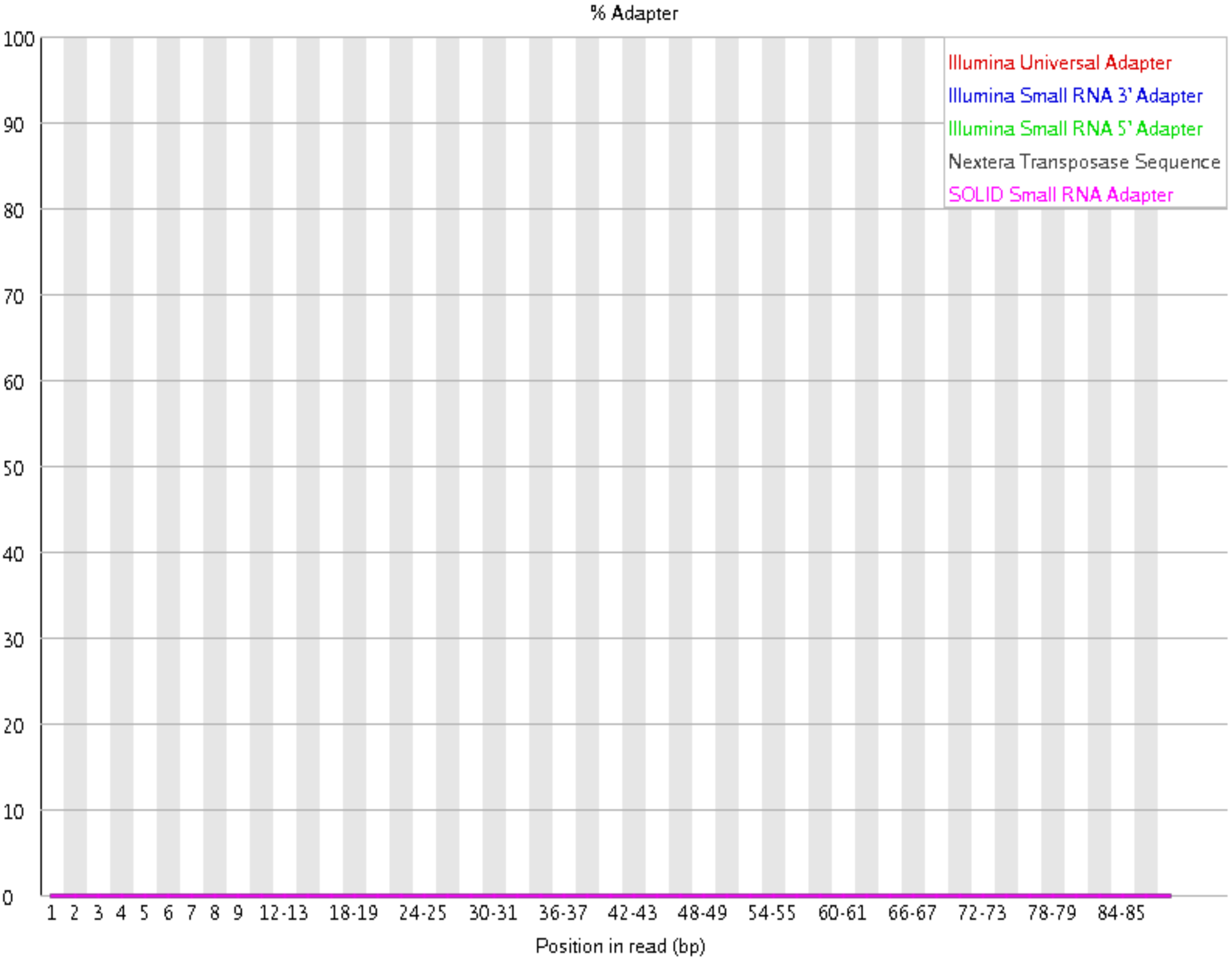

Produced by FastQC (version 0.11.7)

FastQC Report

Sat 31 Mar 2018
stdin

Summary

- Basic Statistics
- Per base sequence quality
- Per tile sequence quality
- Per sequence quality scores
- Per base sequence content
- Per sequence GC content
- Per base N content
- Sequence Length Distribution
- Sequence Duplication Levels
- Overrepresented sequences
- Adapter Content

Basic Statistics

Measure	Value
Filename	stdin
File type	Conventional base calls
Encoding	Sanger / Illumina 1.9
Total Sequences	553277870
Sequences flagged as poor quality	0
Sequence length	35-159
%GC	41

Per base sequence quality

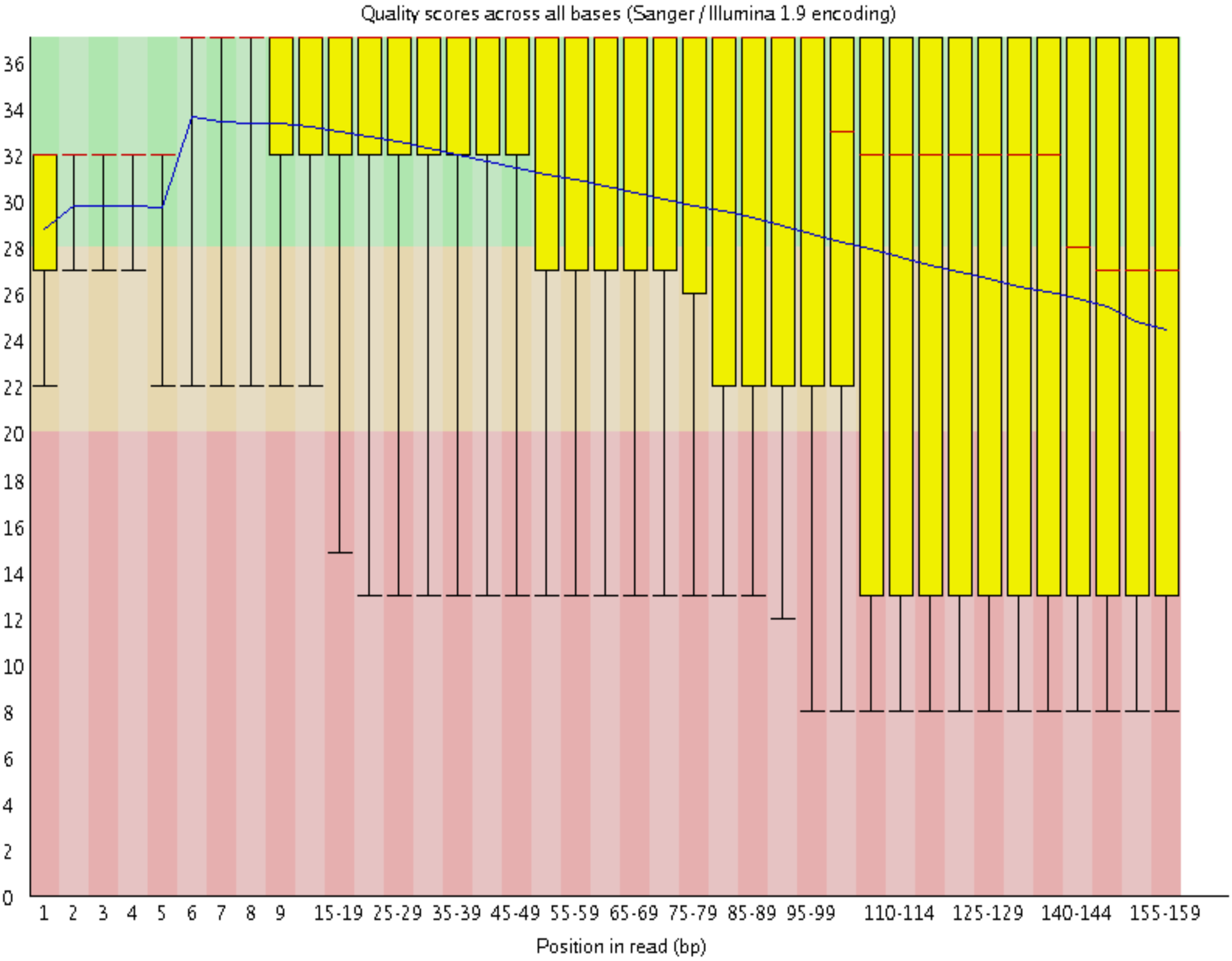

Per tile sequence quality

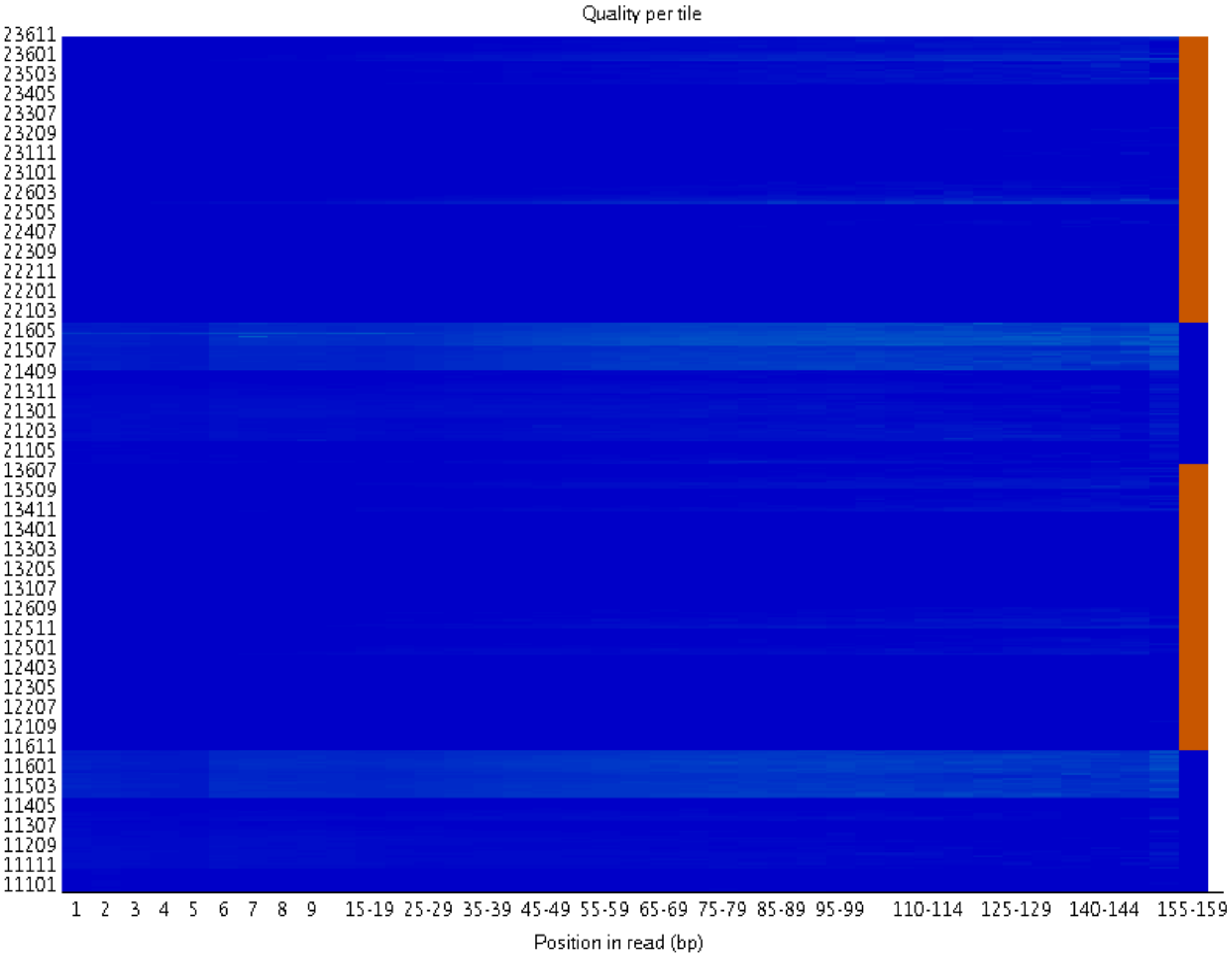

Per sequence quality scores

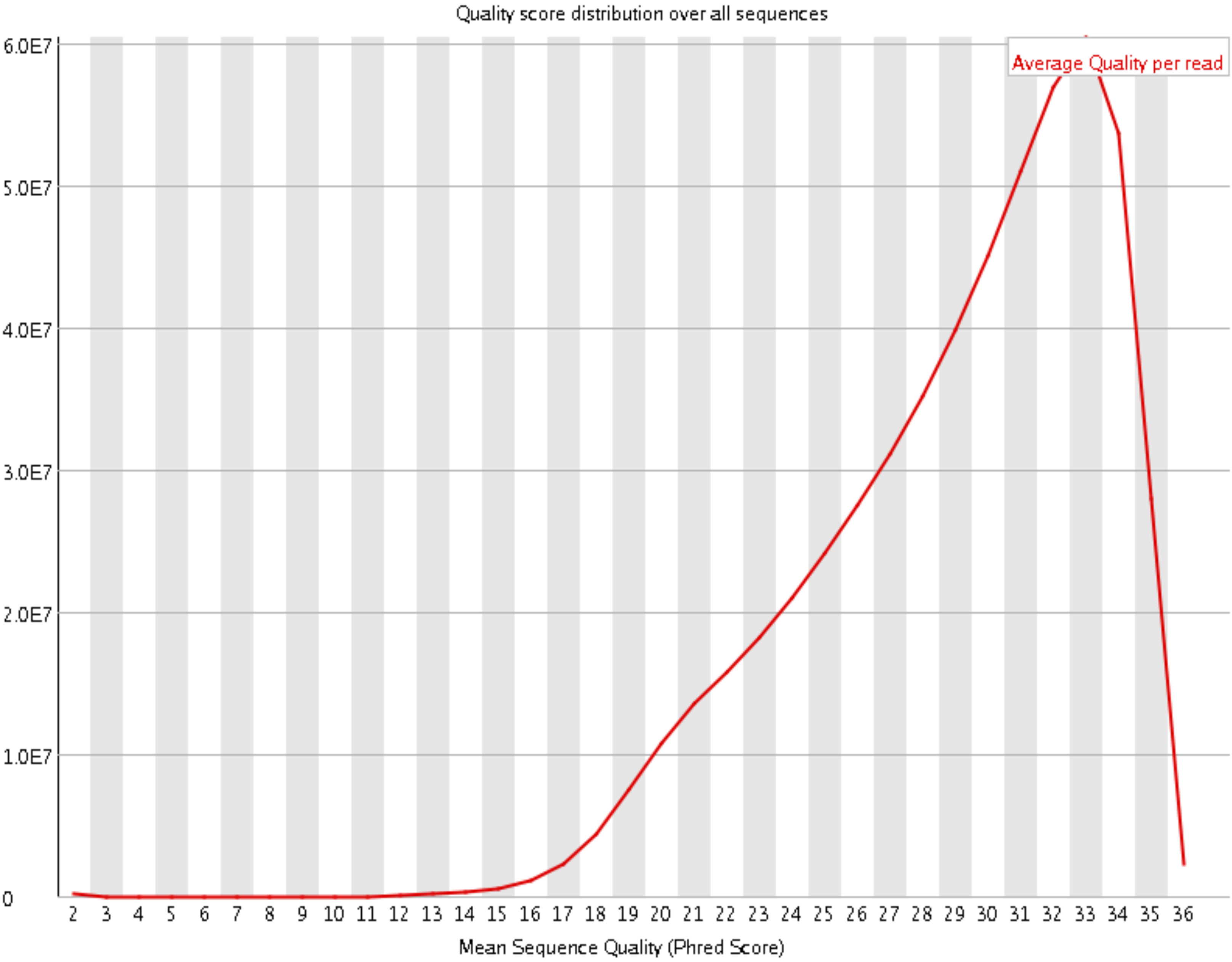

Per base sequence content

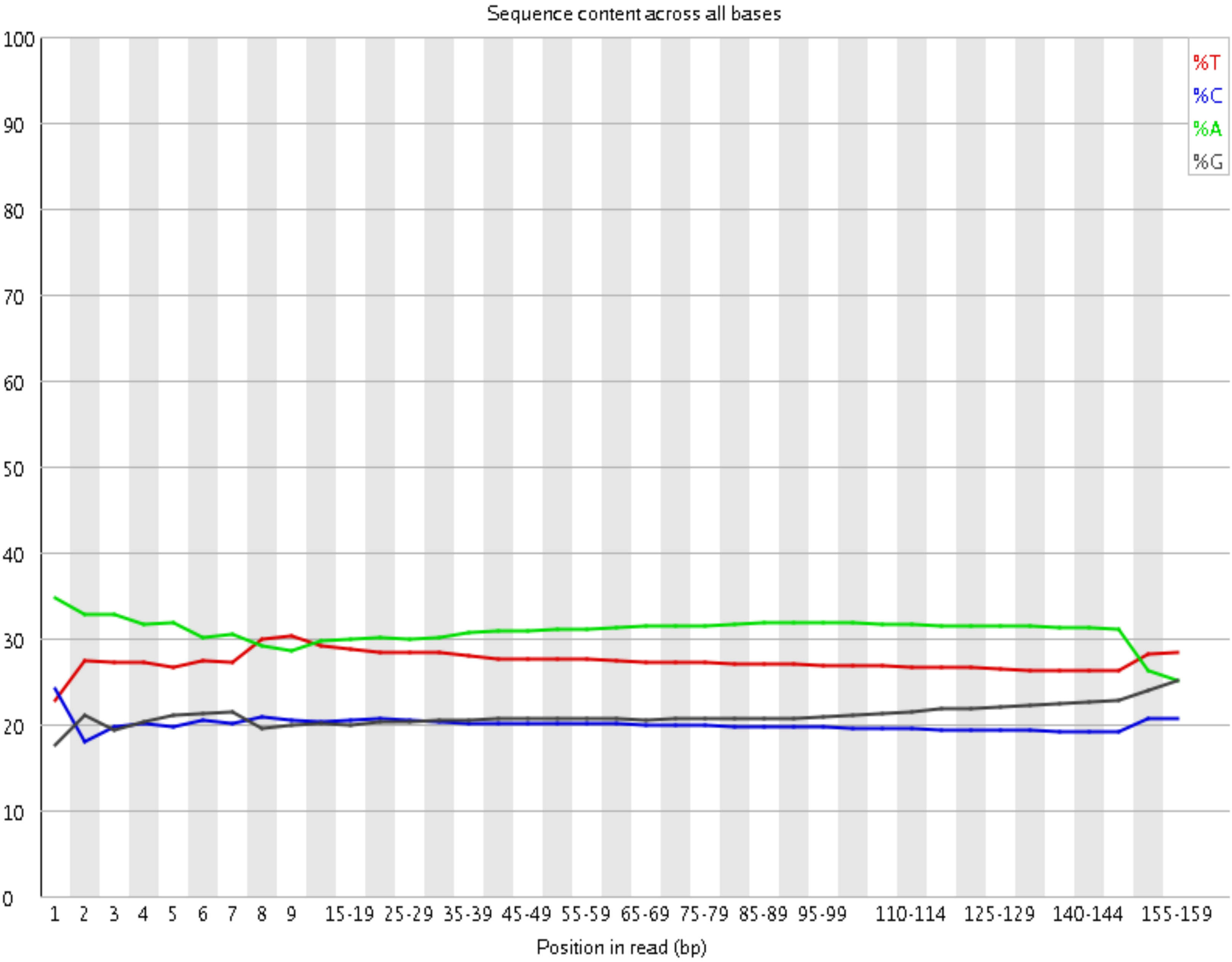

Per sequence GC content

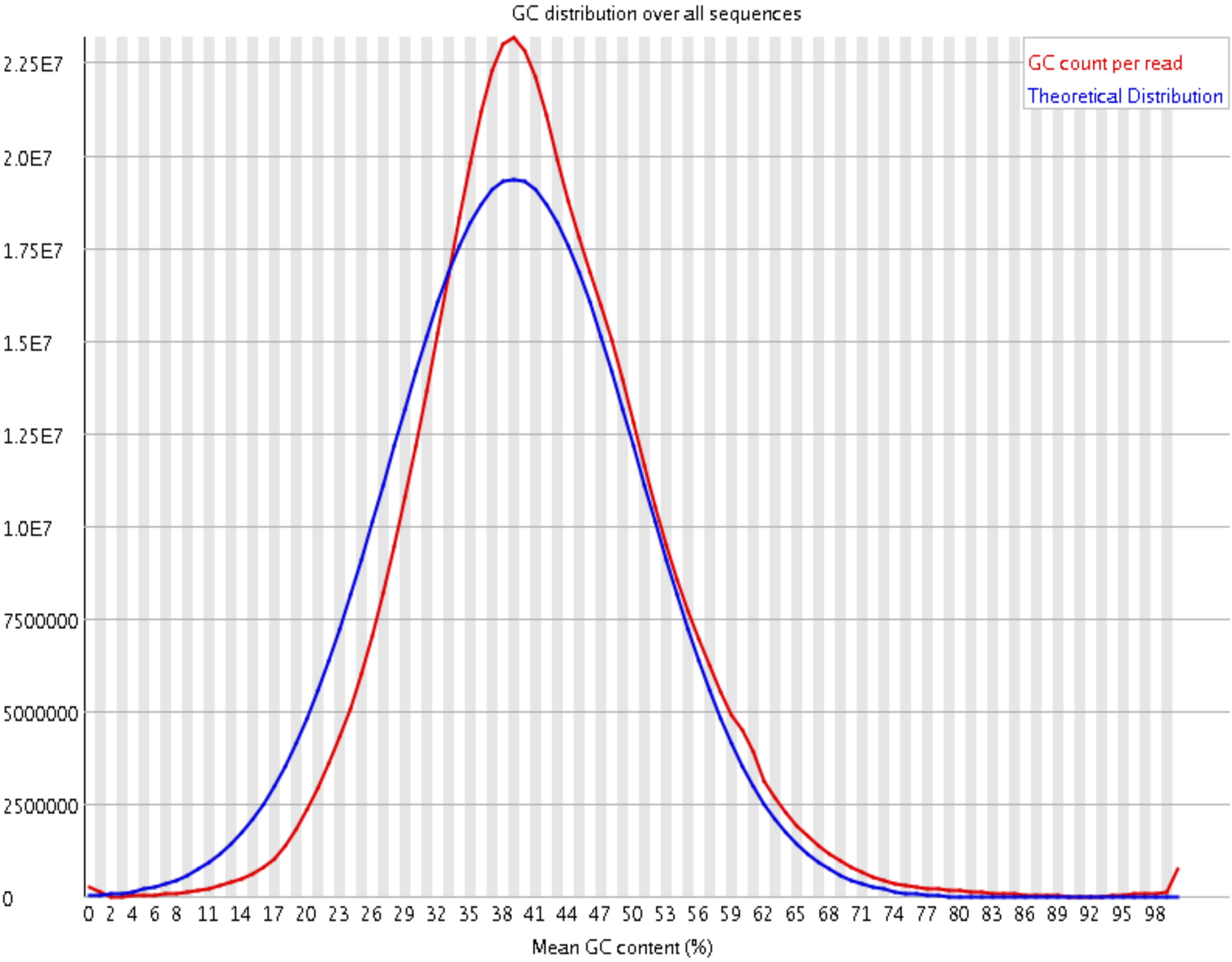

Per base N content

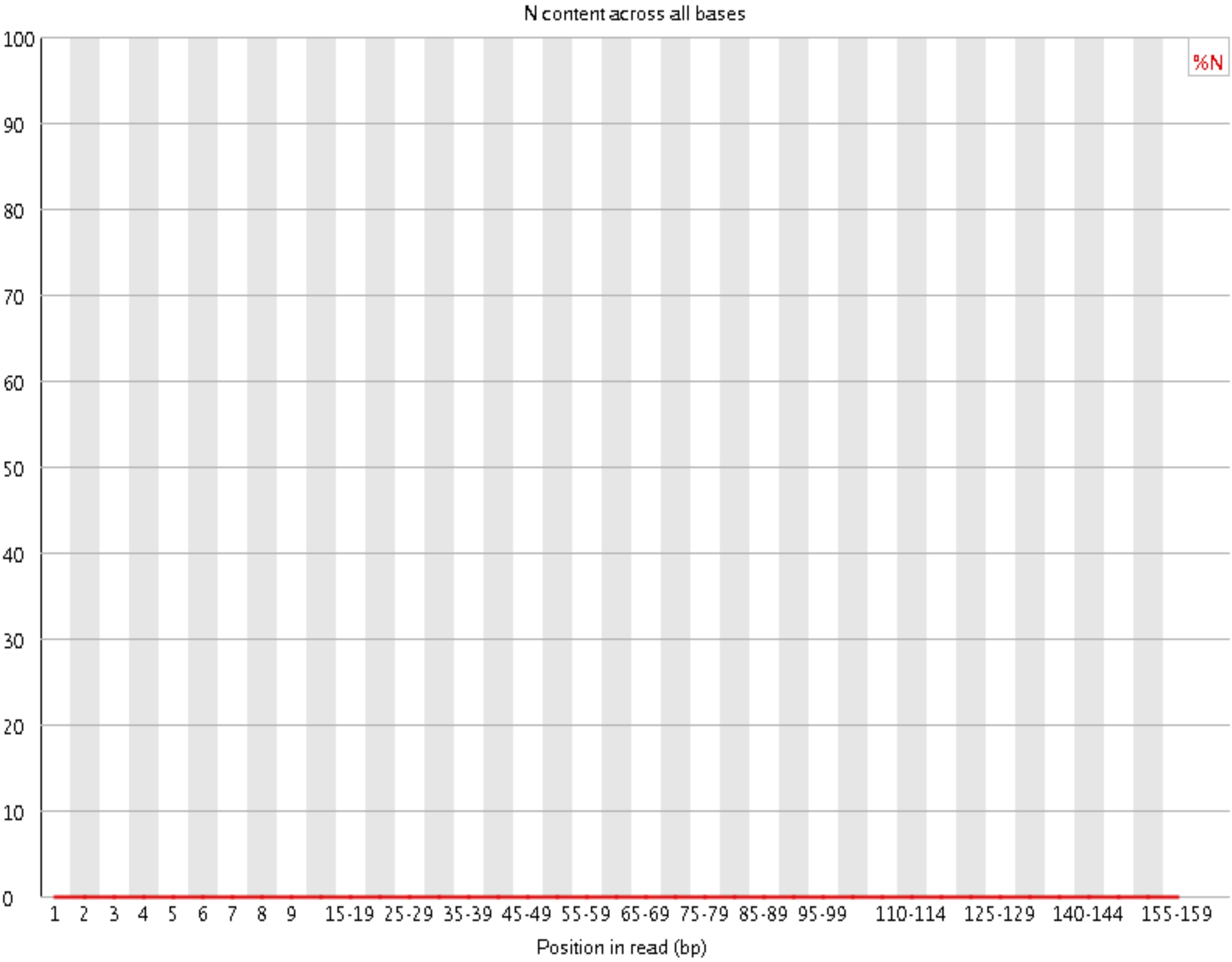

Sequence Length Distribution

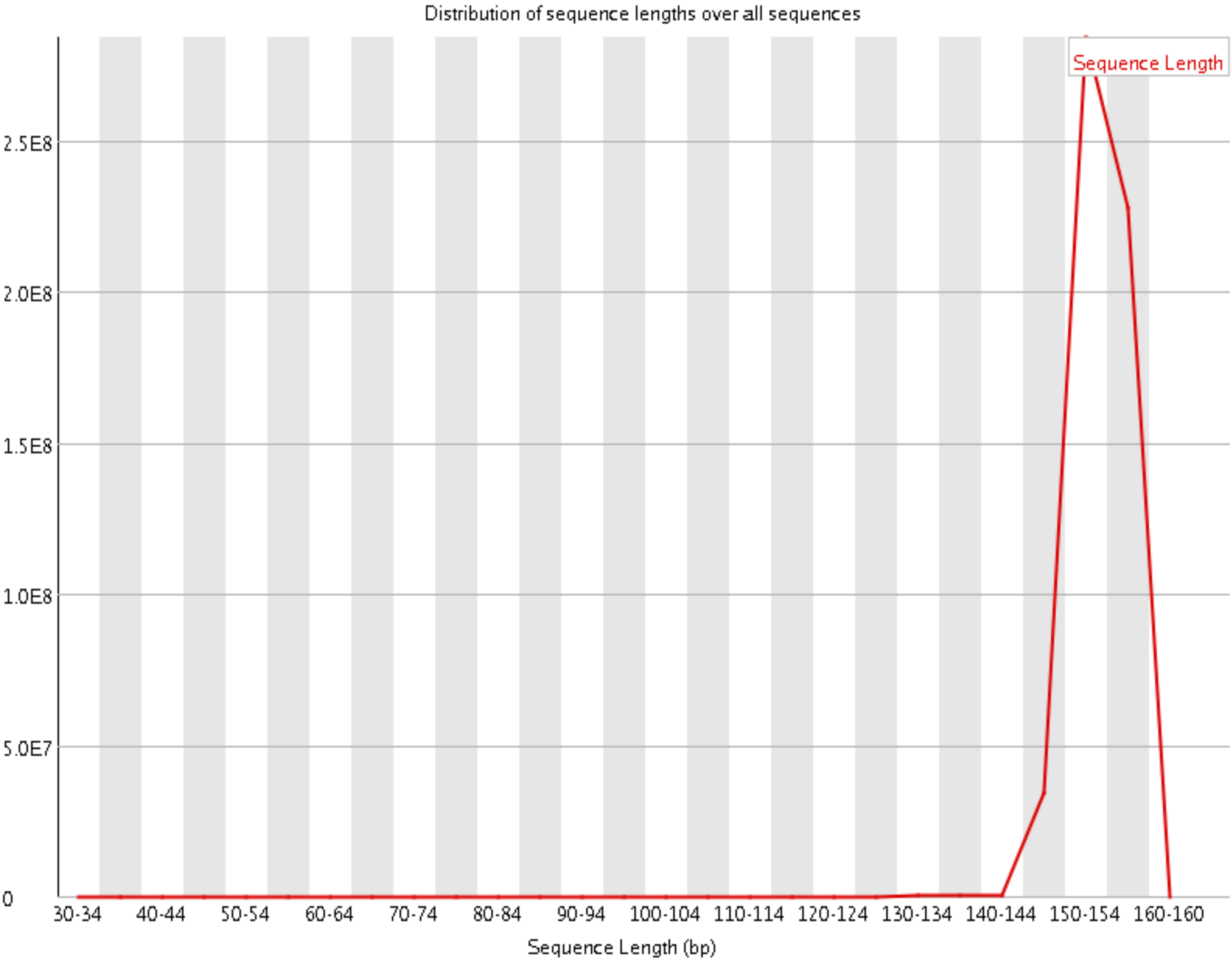

Sequence Duplication Levels

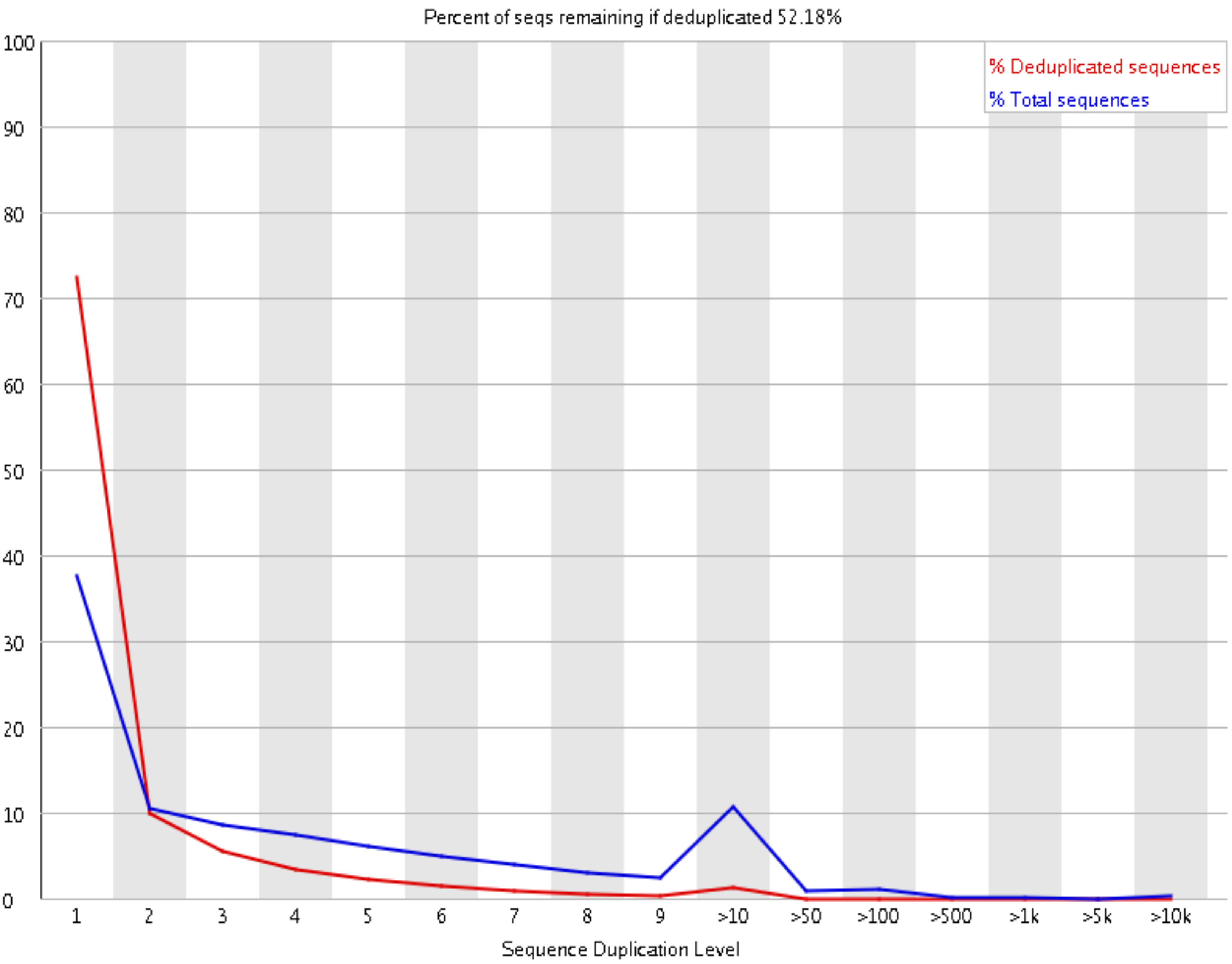

Overrepresented sequences

Sequence	Count	Percentage	Possible Source
GGGGGGGGGGGGGGGGGGGGGGGGGGGGGGGGGGGGGGGGGGGGGGGGGG	1464007	0.2646061010898556	No Hit

Adapter Content

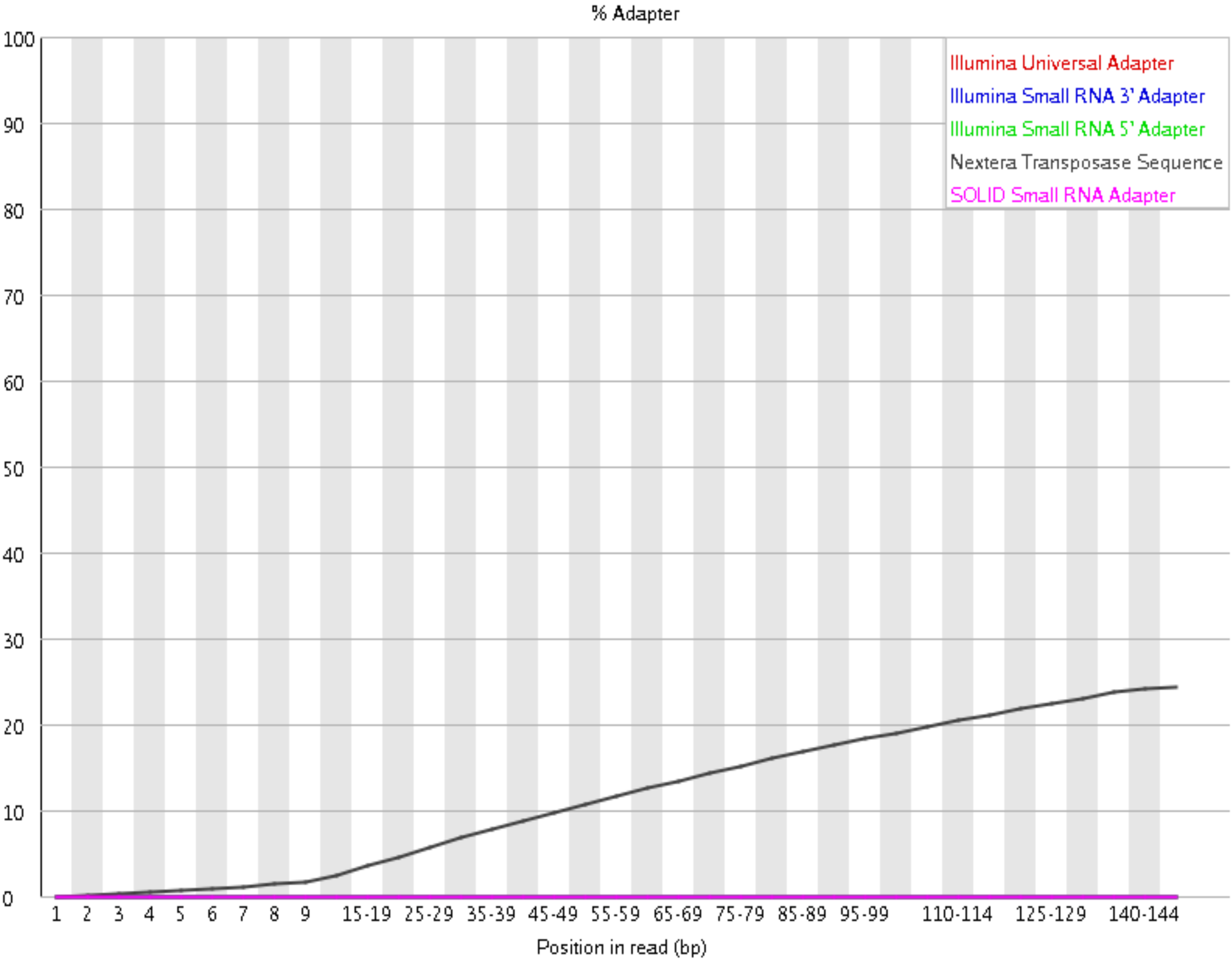

Produced by FastQC (version 0.11.7)

FastQC Report

Sat 31 Mar 2018
stdin

Summary

- Basic Statistics
- Per base sequence quality
- Per tile sequence quality
- Per sequence quality scores
- Per base sequence content
- Per sequence GC content
- Per base N content
- Sequence Length Distribution
- Sequence Duplication Levels
- Overrepresented sequences
- Adapter Content

Basic Statistics

Measure	Value
Filename	stdin
File type	Conventional base calls
Encoding	Sanger / Illumina 1.9
Total Sequences	338626576
Sequences flagged as poor quality	0
Sequence length	35-156
%GC	43

Per base sequence quality

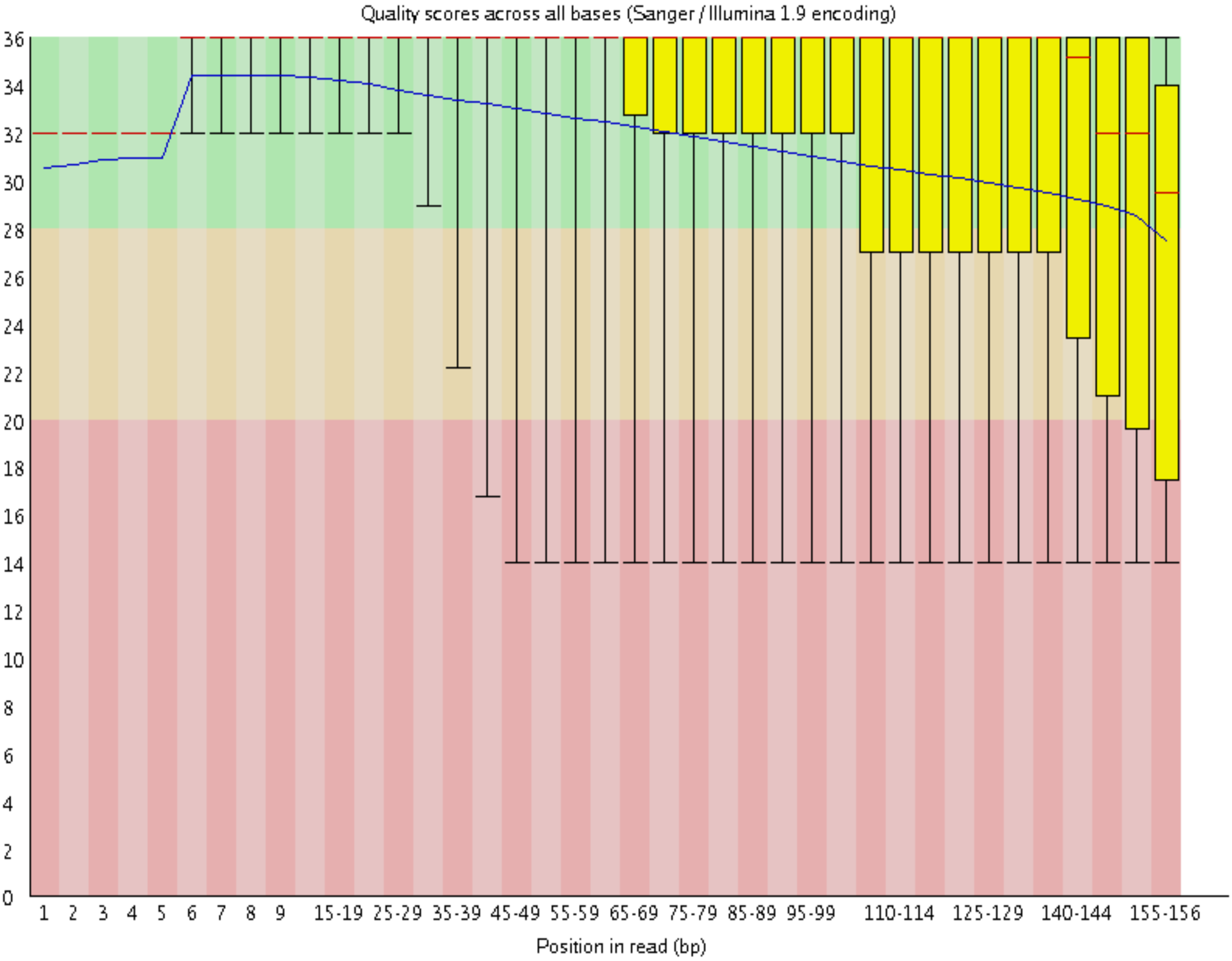

Per tile sequence quality

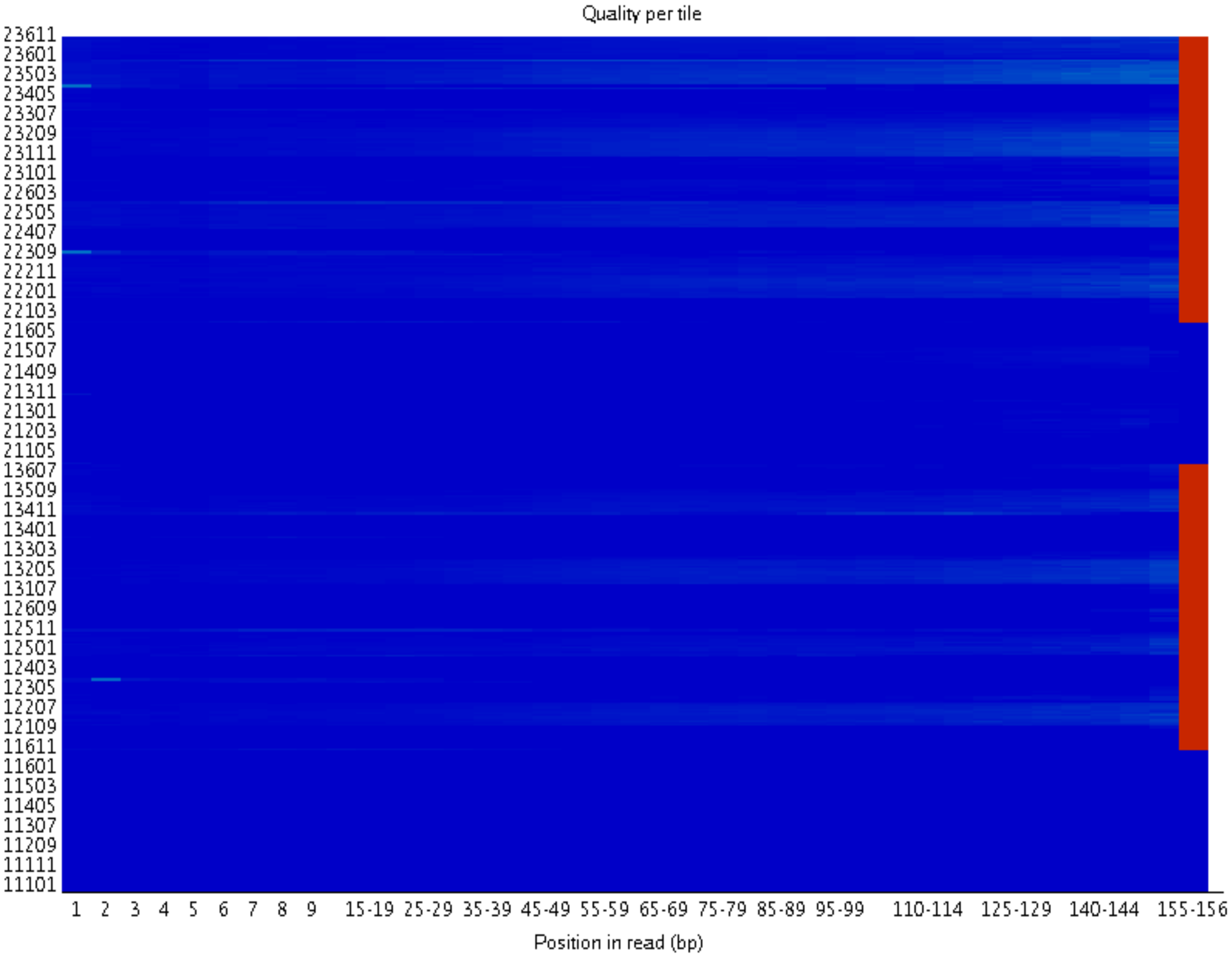

Per sequence quality scores

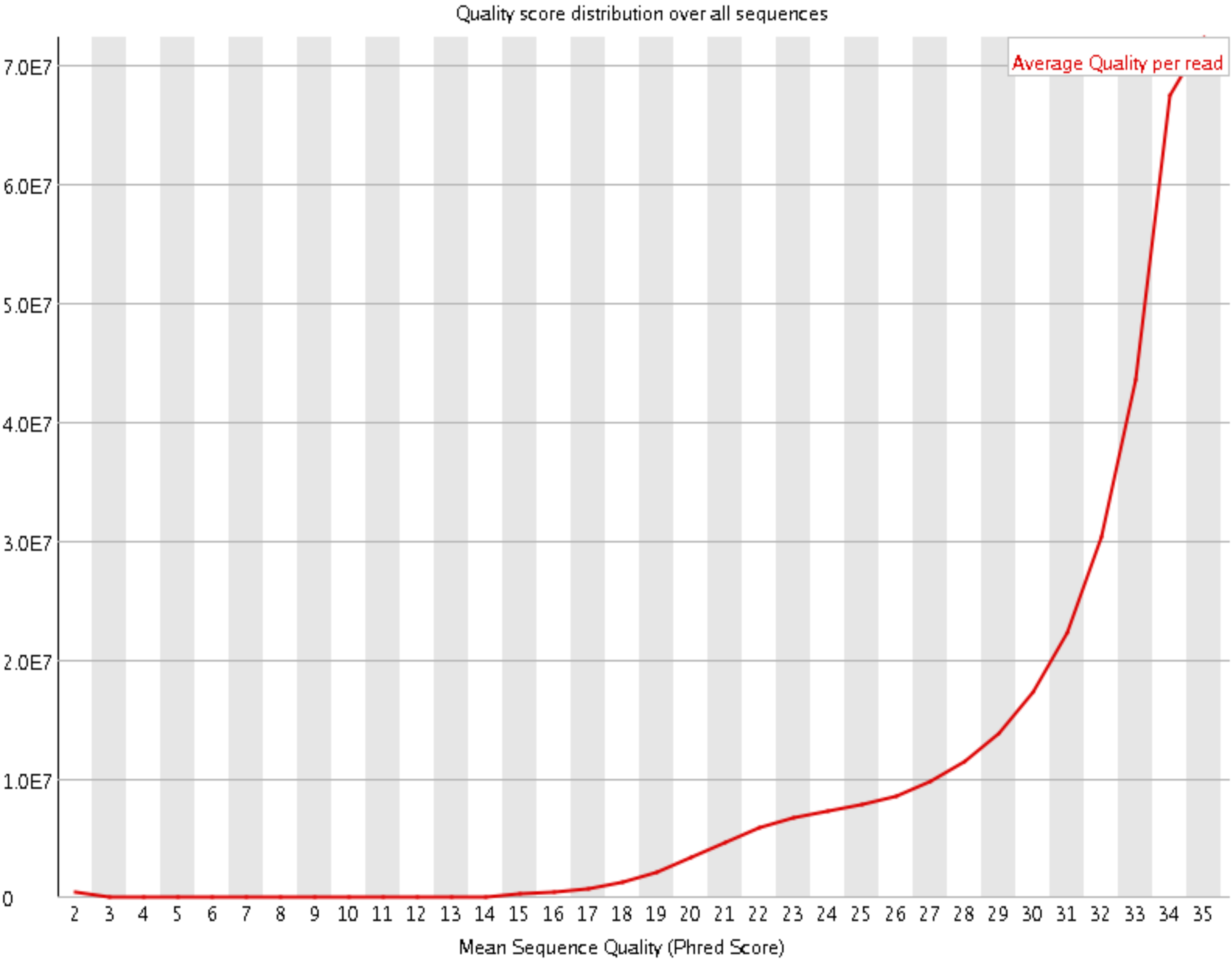

Per base sequence content

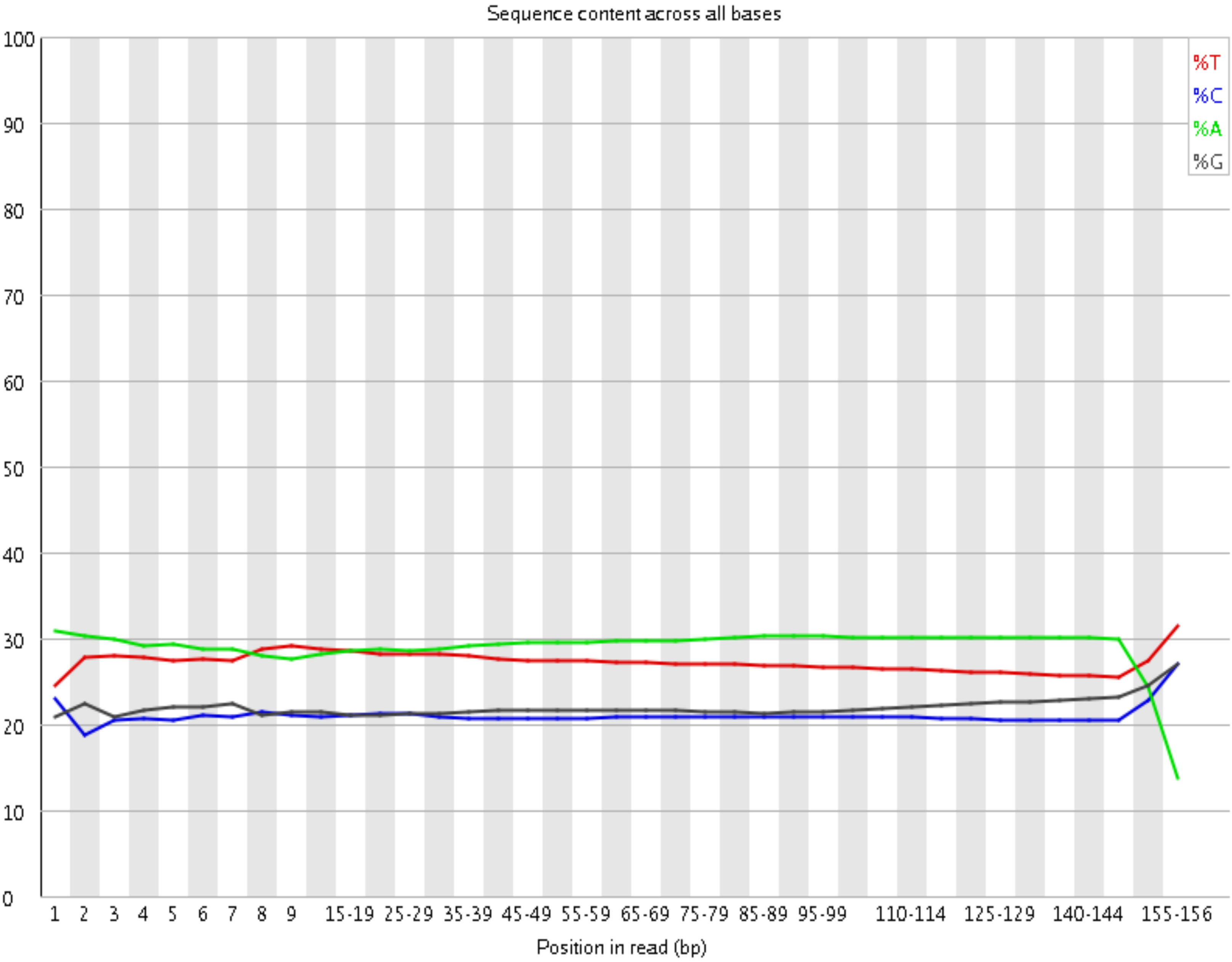

Per sequence GC content

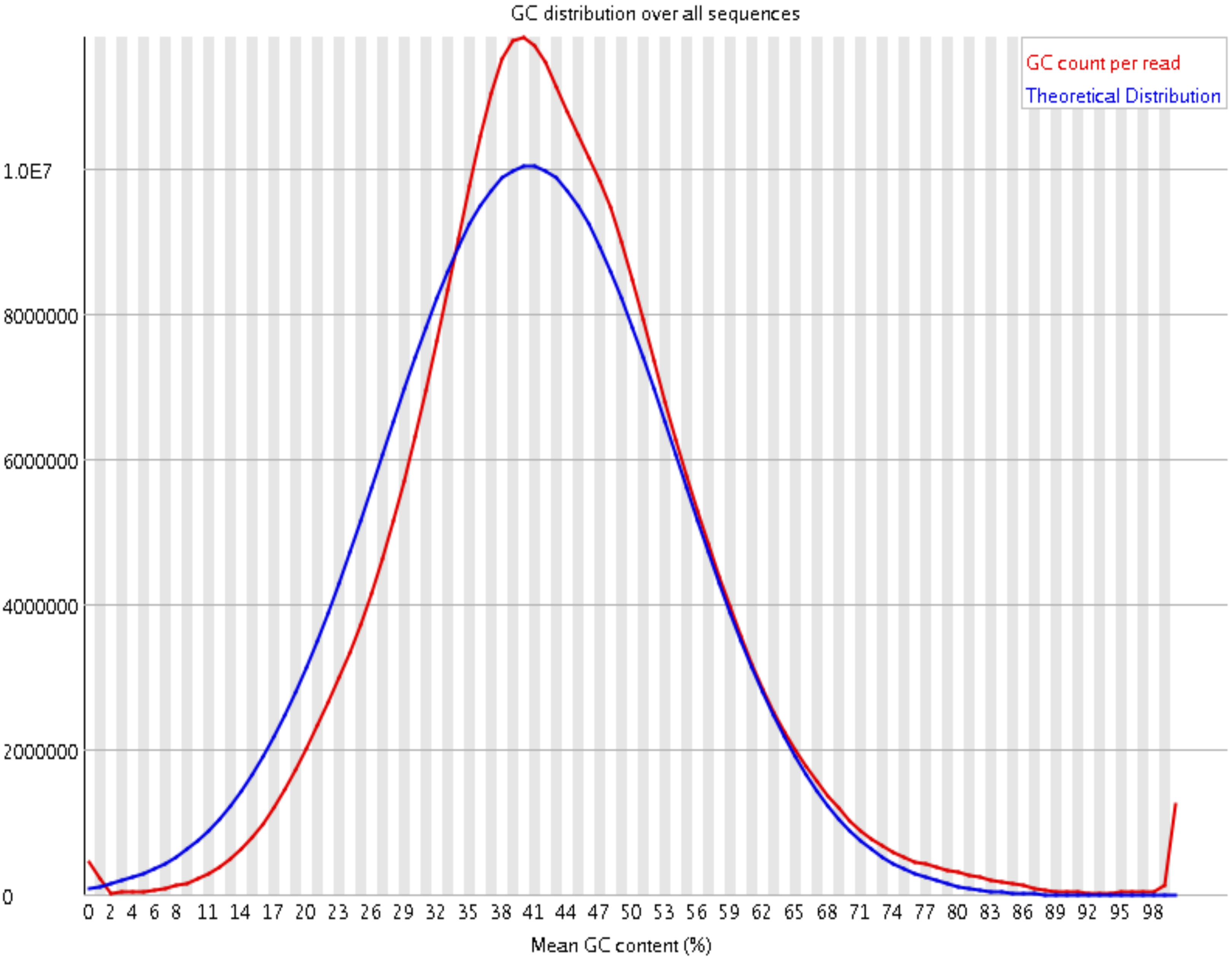

Per base N content

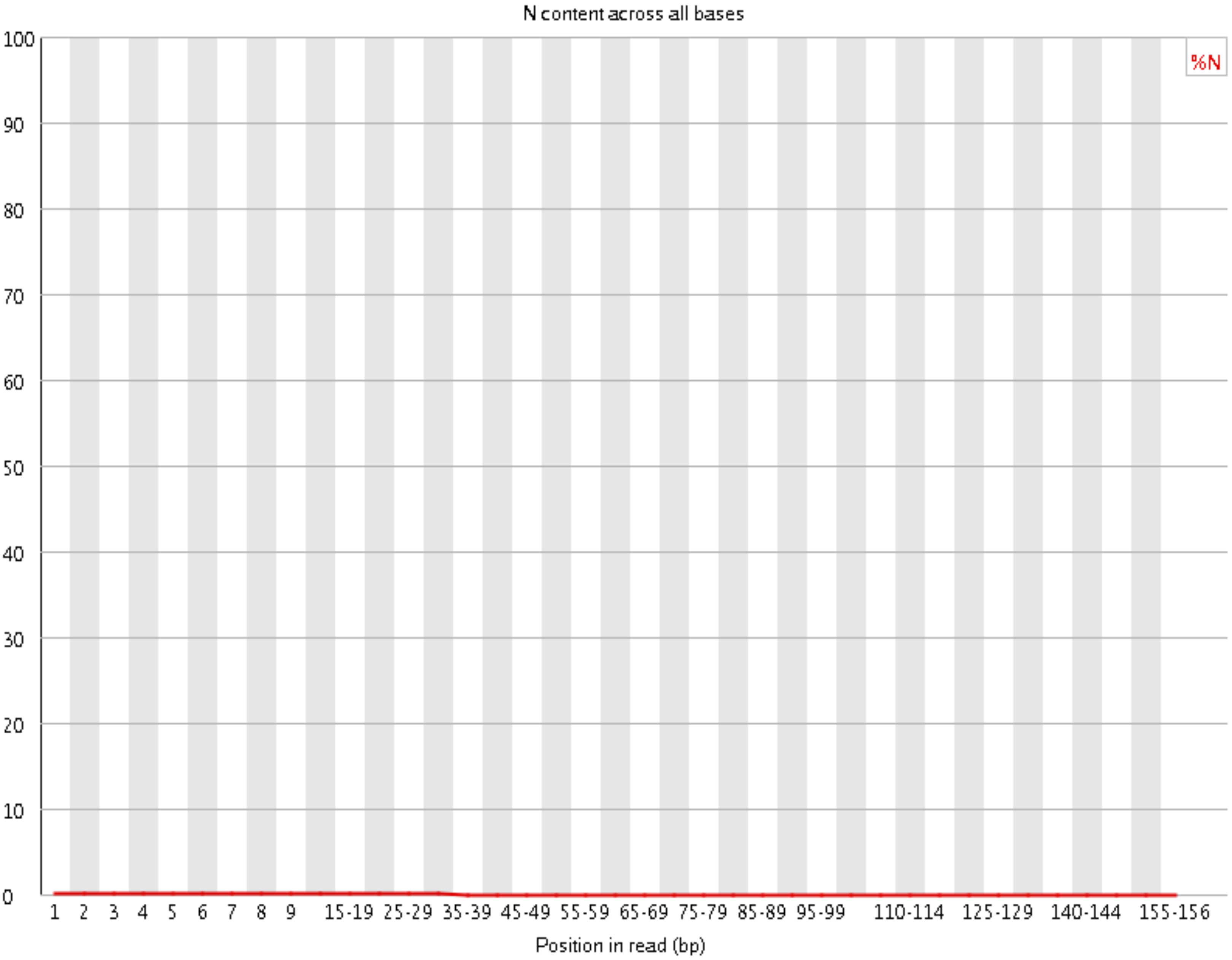

Sequence Length Distribution

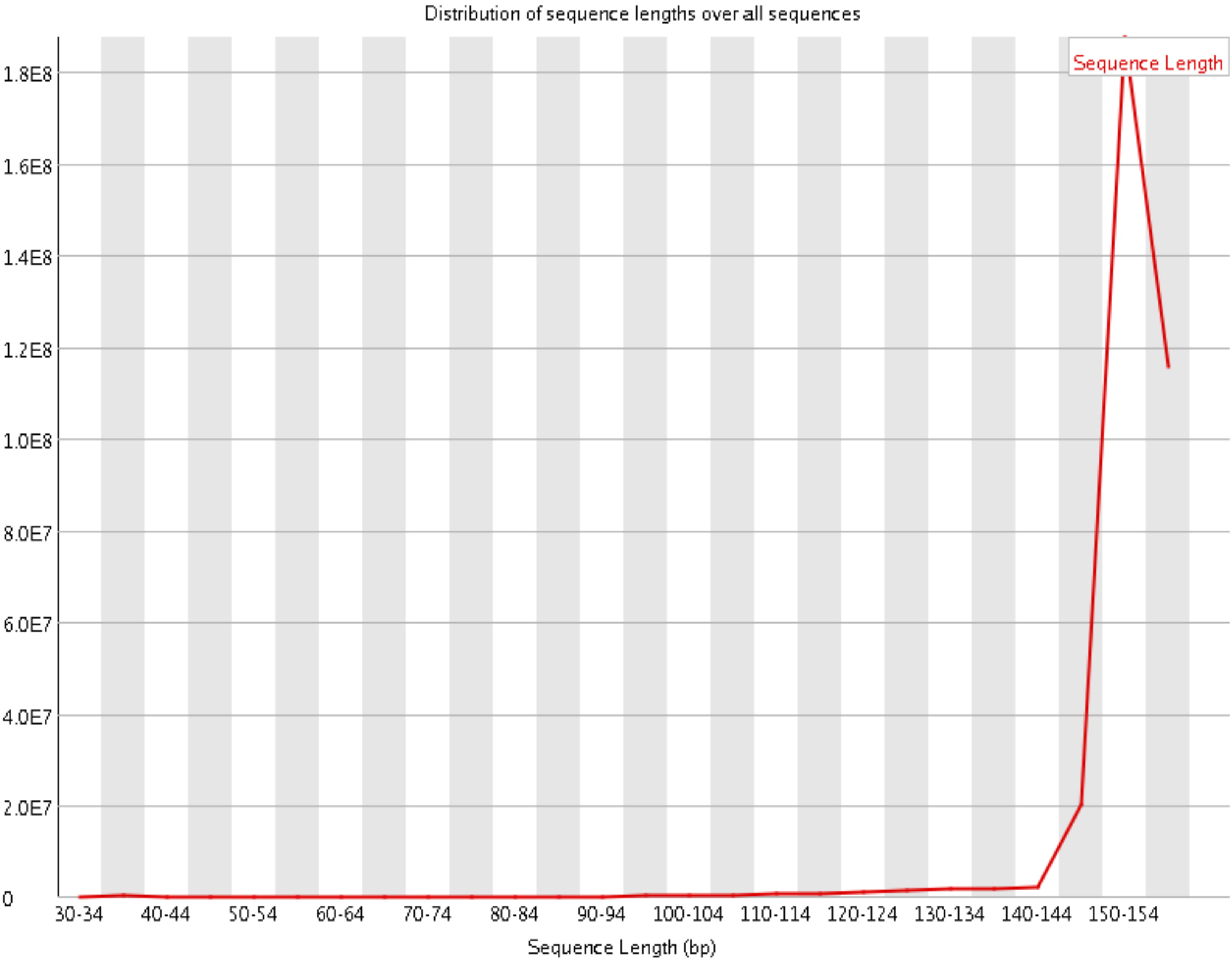

Sequence Duplication Levels

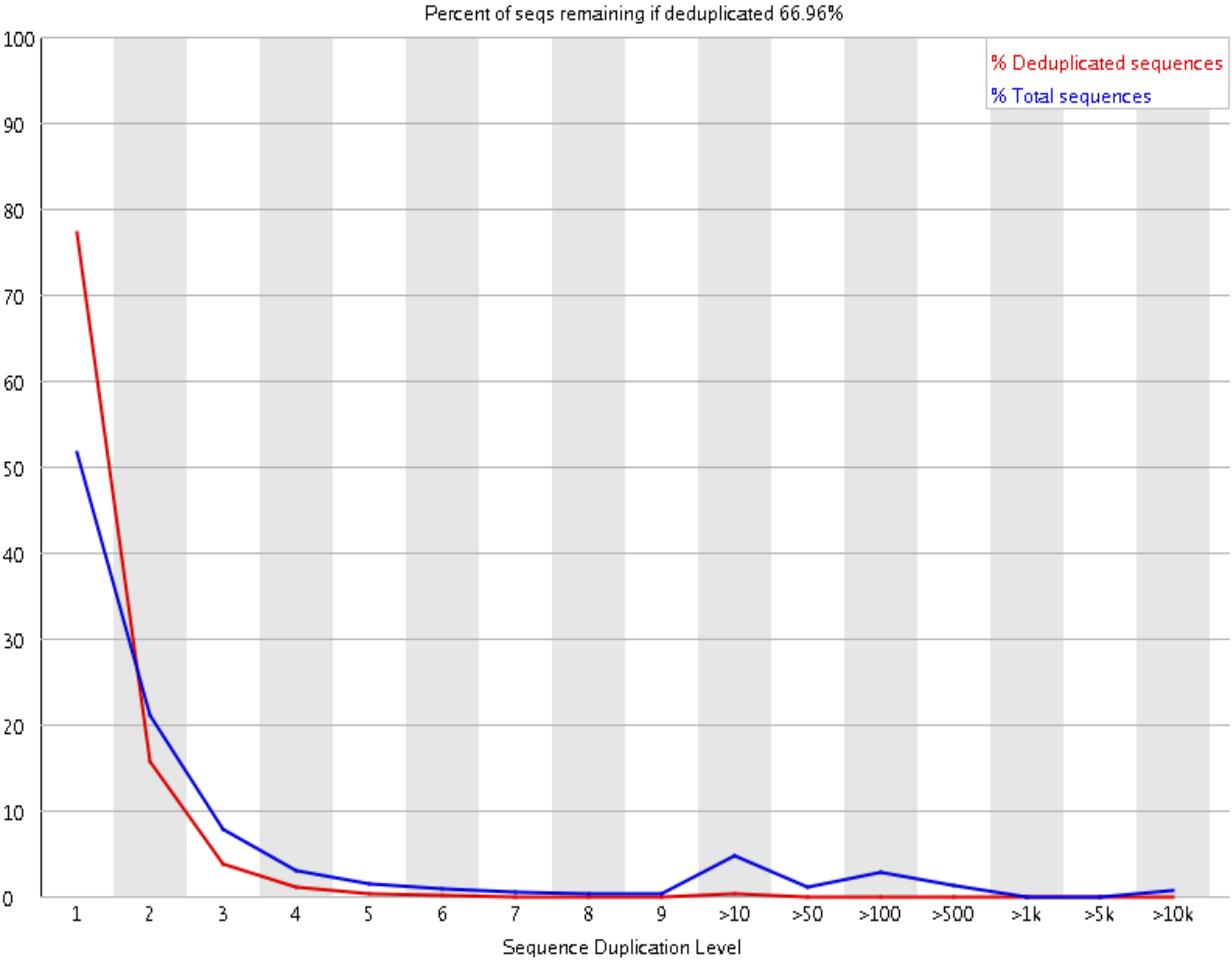

Overrepresented sequences

Sequence	Count	Percentage	Possible Source
GGGGGGGGGGGGGGGGGGGGGGGGGGGGGGGGGGGGGGGGGGGGGGGGGG	2384371	0.7041299085751616	No Hit
NNNNNNNNNNNNNNNNNNNNNNNNNNNNNNNNNNN	414840	0.1225066280680817	No Hit

Adapter Content

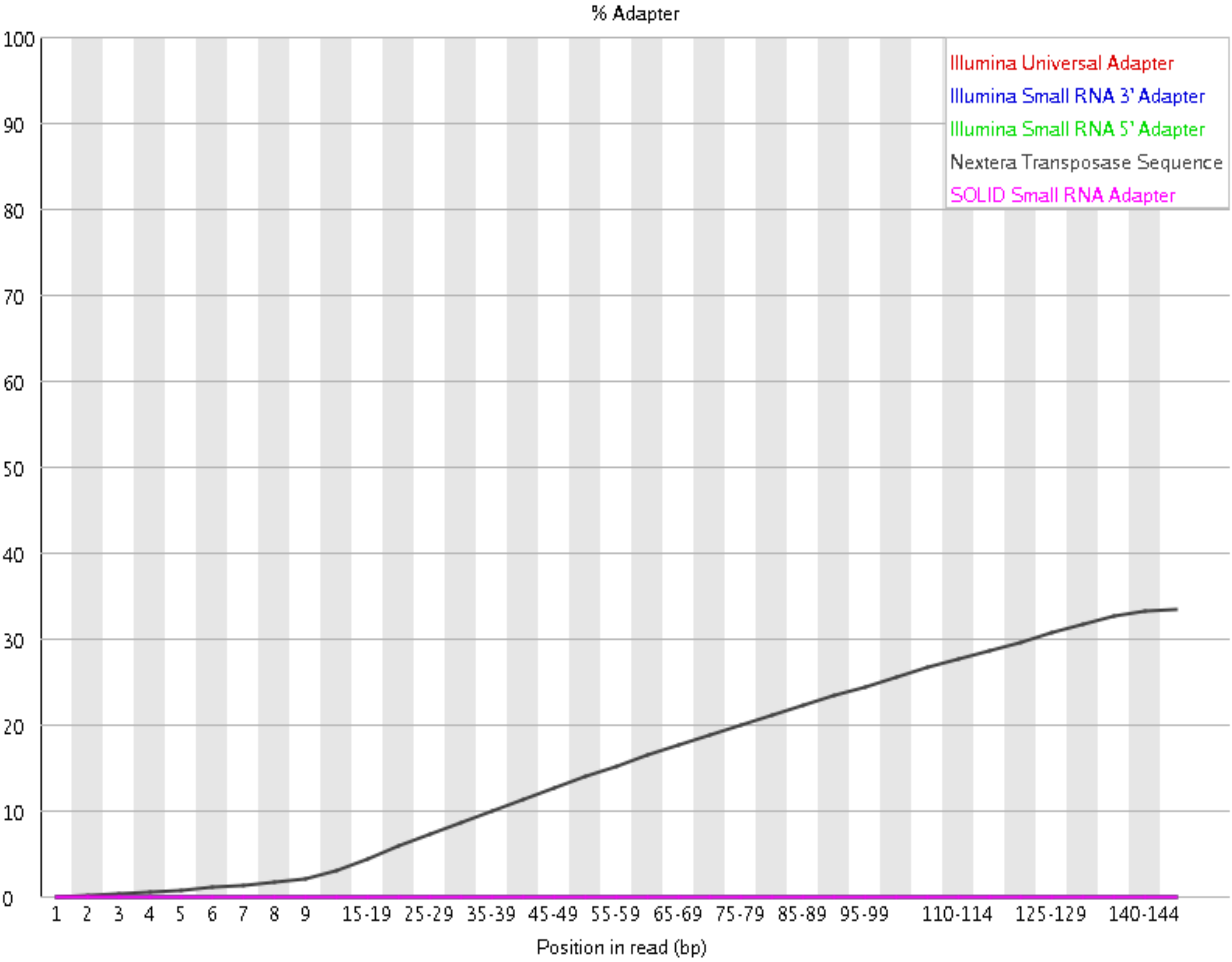

Produced by FastQC (version 0.11.7)

FastQC Report

Sat 31 Mar 2018
stdin

Summary

- Basic Statistics
- Per base sequence quality
- Per tile sequence quality
- Per sequence quality scores
- Per base sequence content
- Per sequence GC content
- Per base N content
- Sequence Length Distribution
- Sequence Duplication Levels
- Overrepresented sequences
- Adapter Content

Basic Statistics

Measure	Value
Filename	stdin
File type	Conventional base calls
Encoding	Sanger / Illumina 1.9
Total Sequences	366199264
Sequences flagged as poor quality	0
Sequence length	35-151
%GC	40

Per base sequence quality

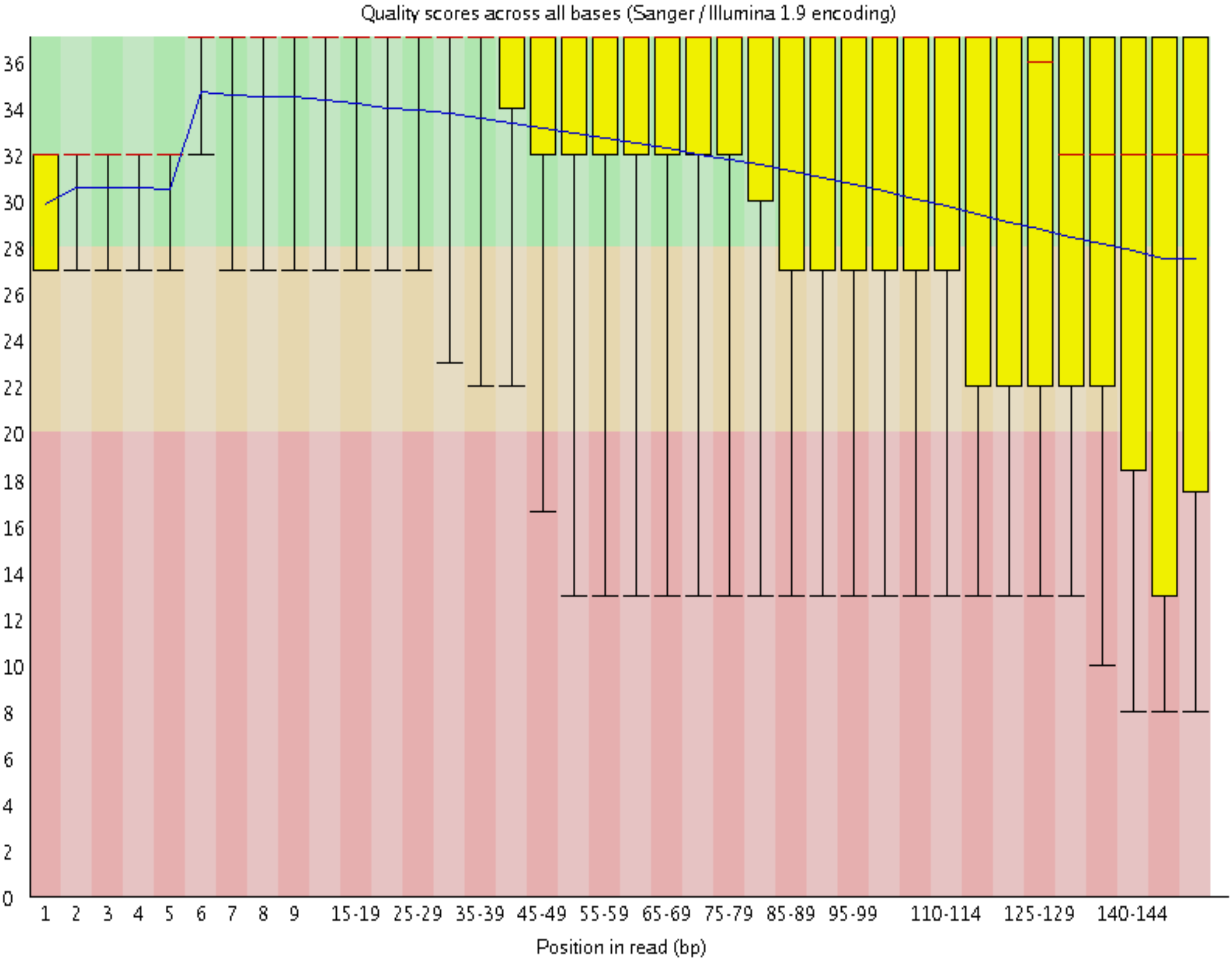

Per tile sequence quality

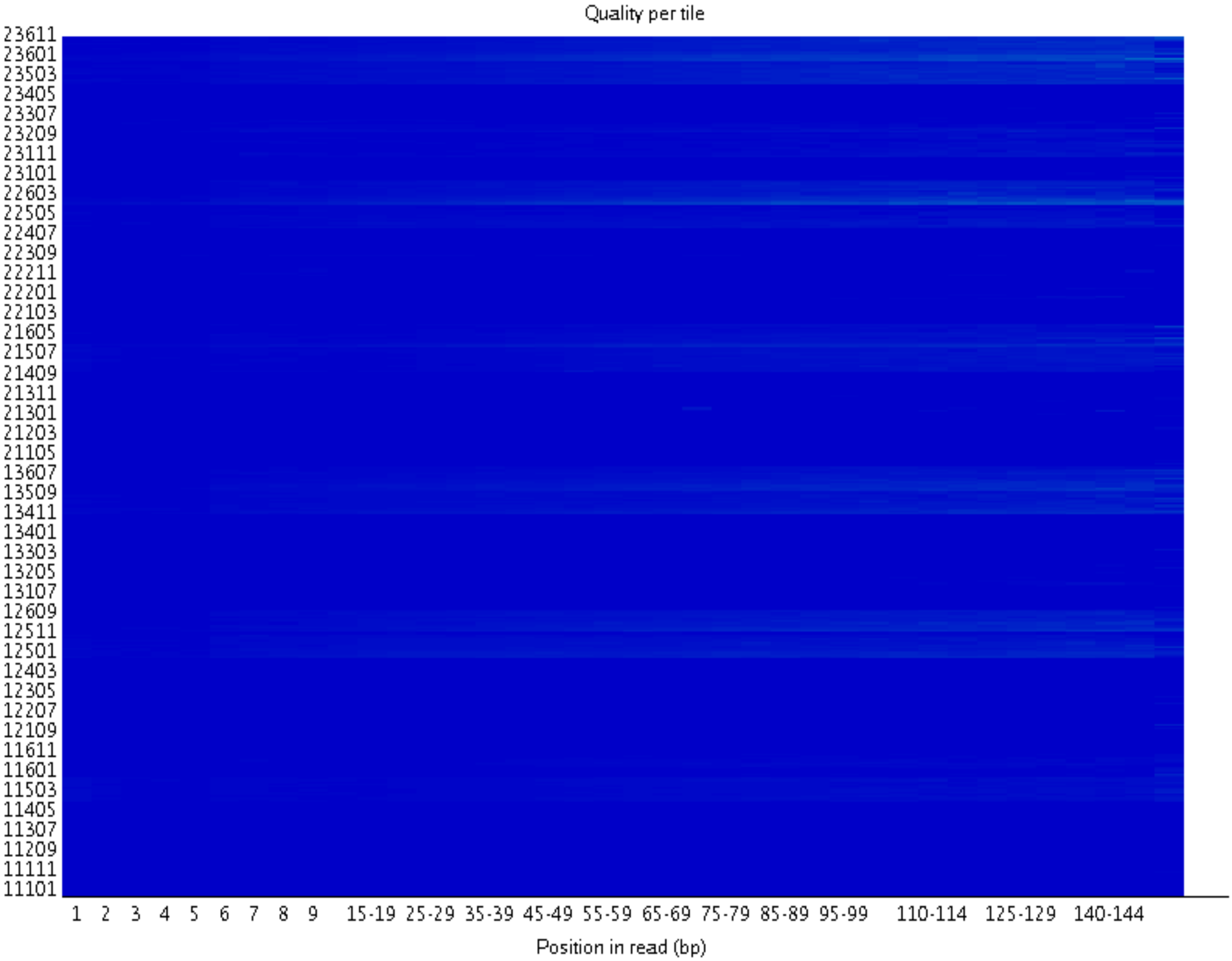

Per sequence quality scores

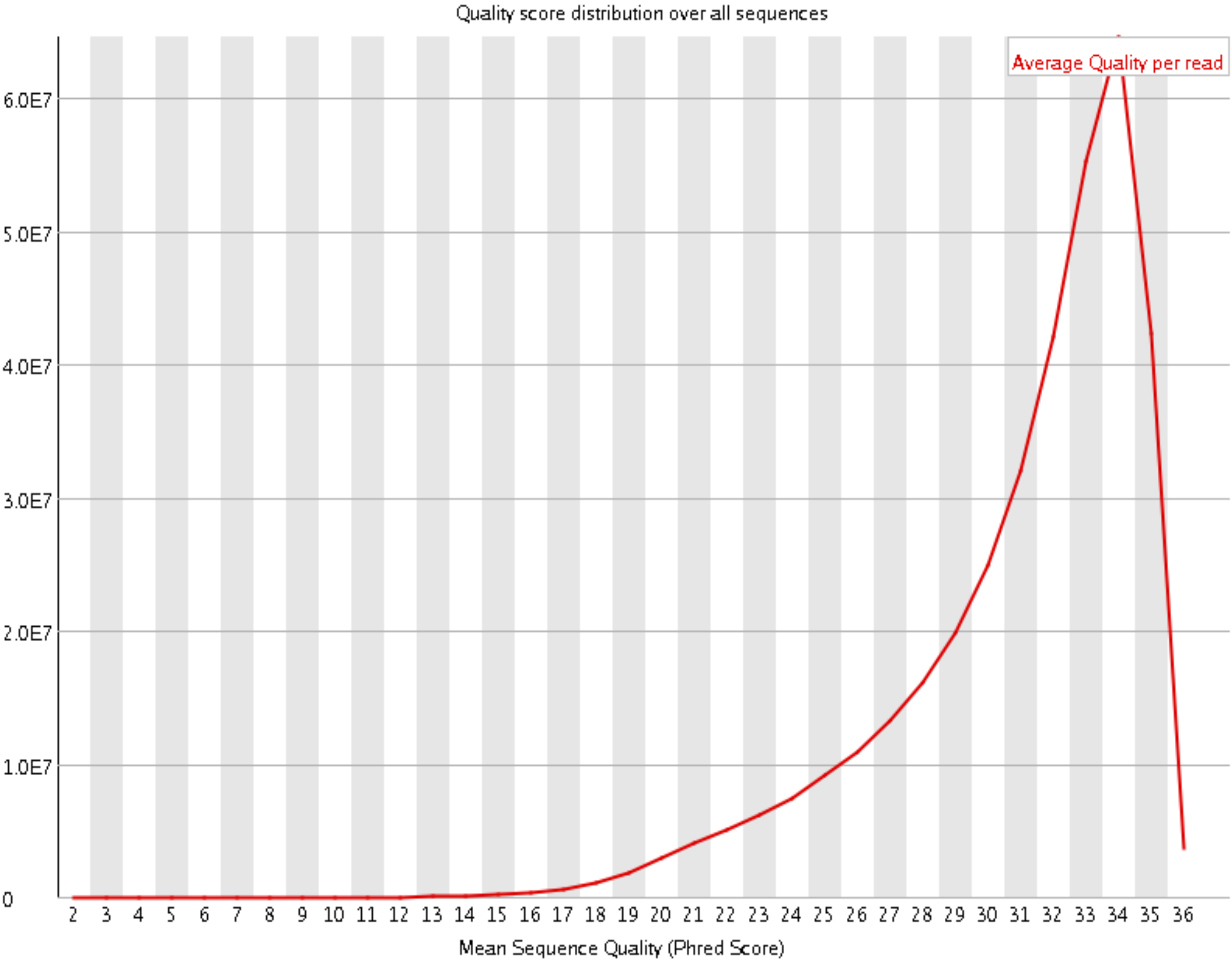

Per base sequence content

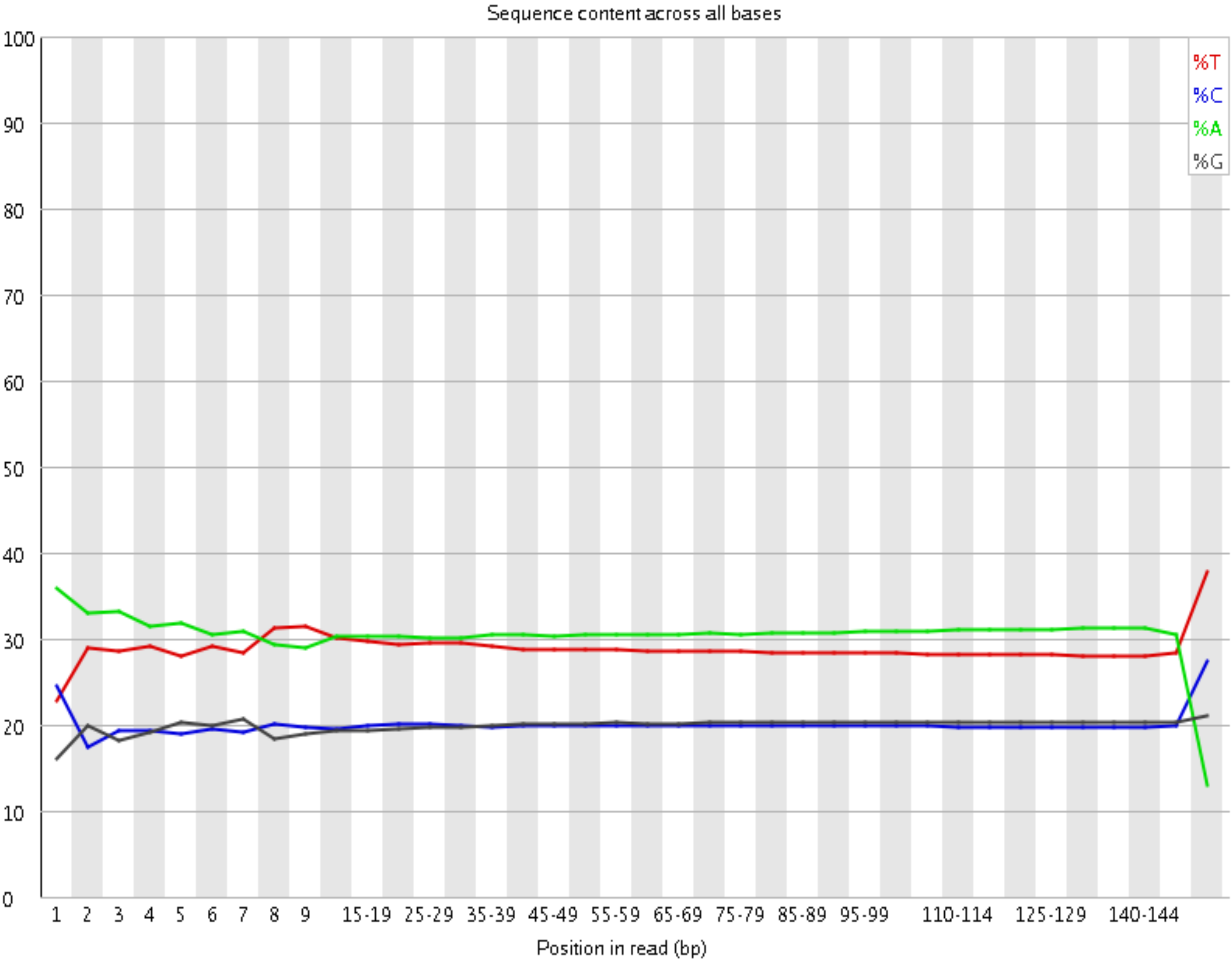

Per sequence GC content

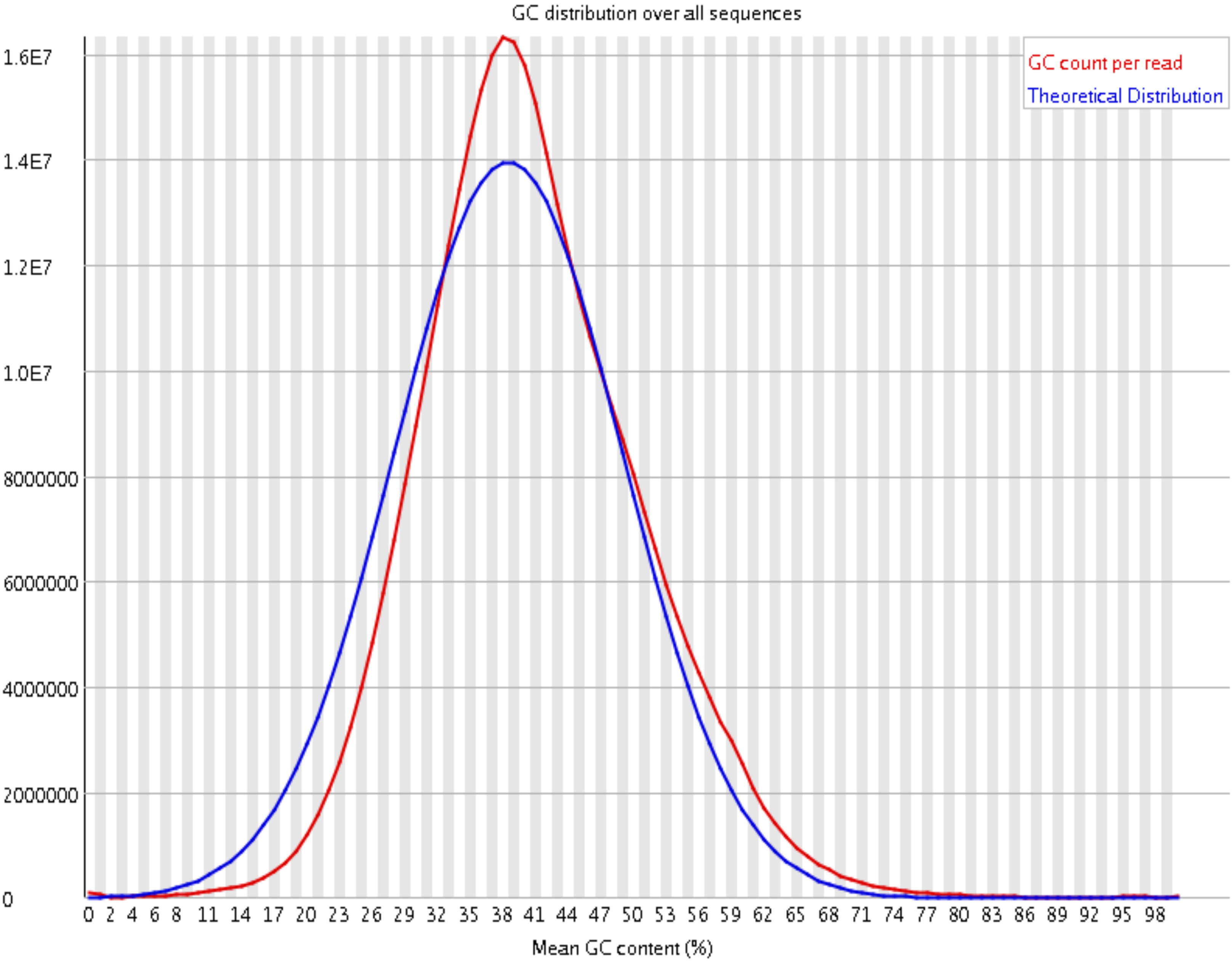

Per base N content

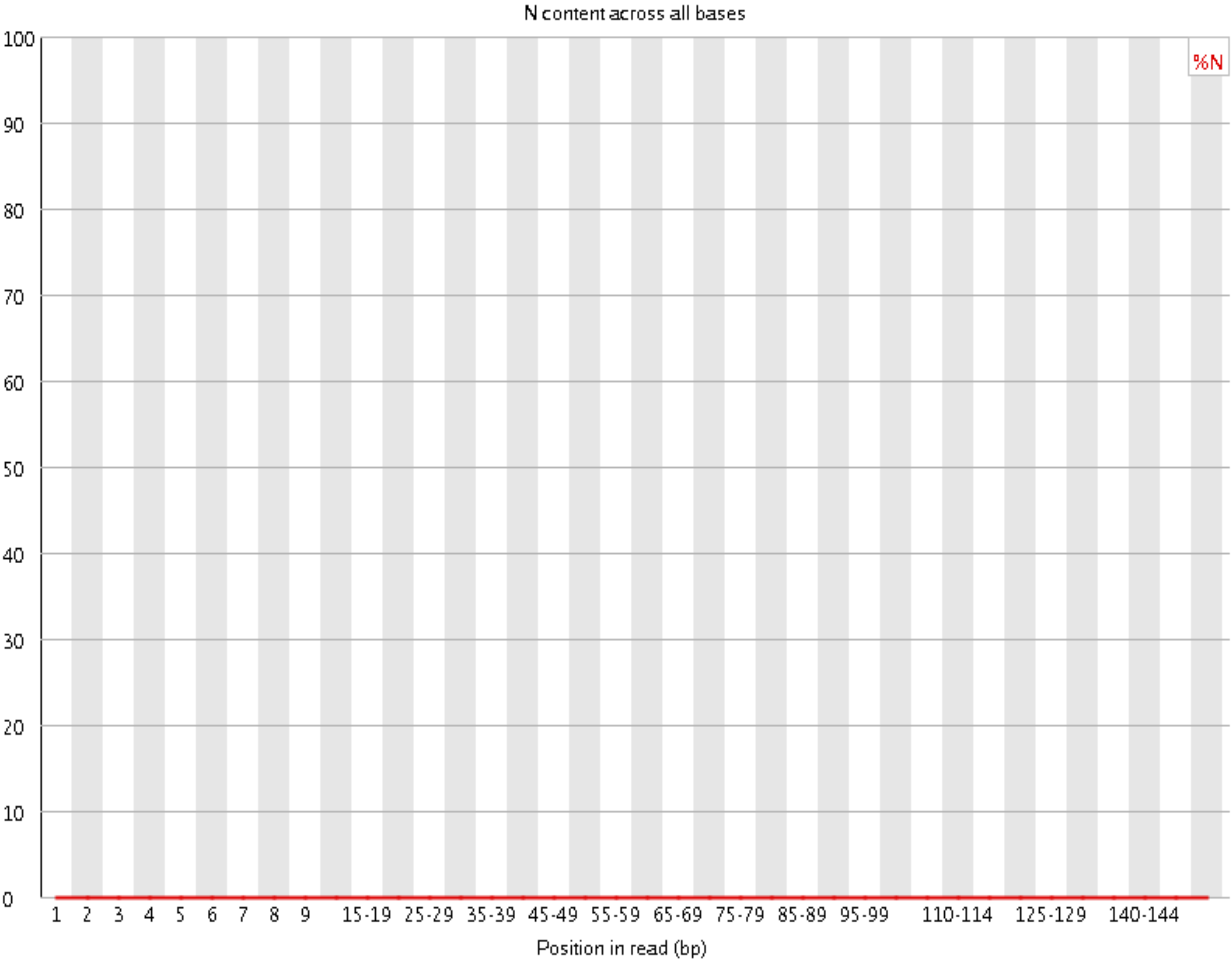

Sequence Length Distribution

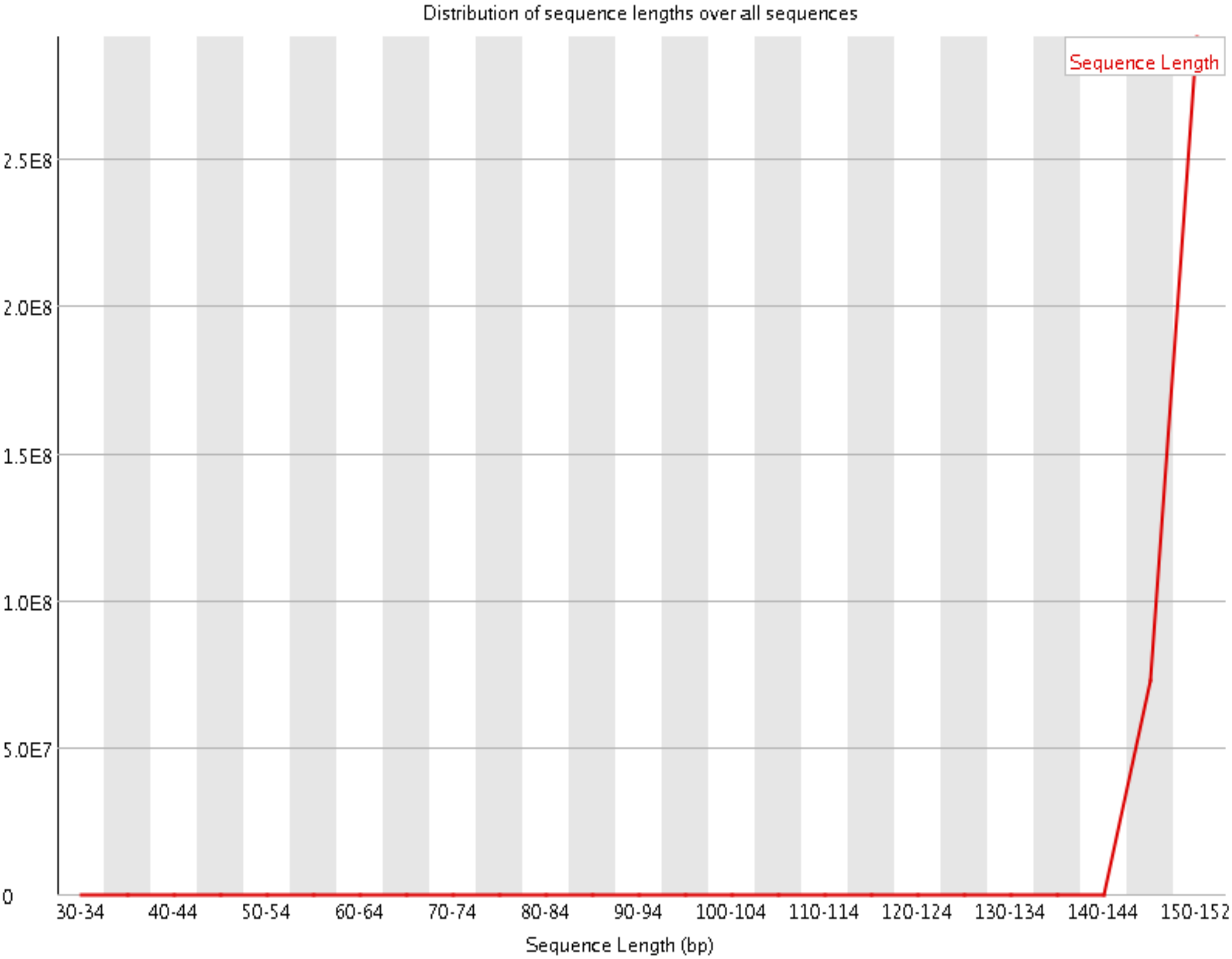

Sequence Duplication Levels

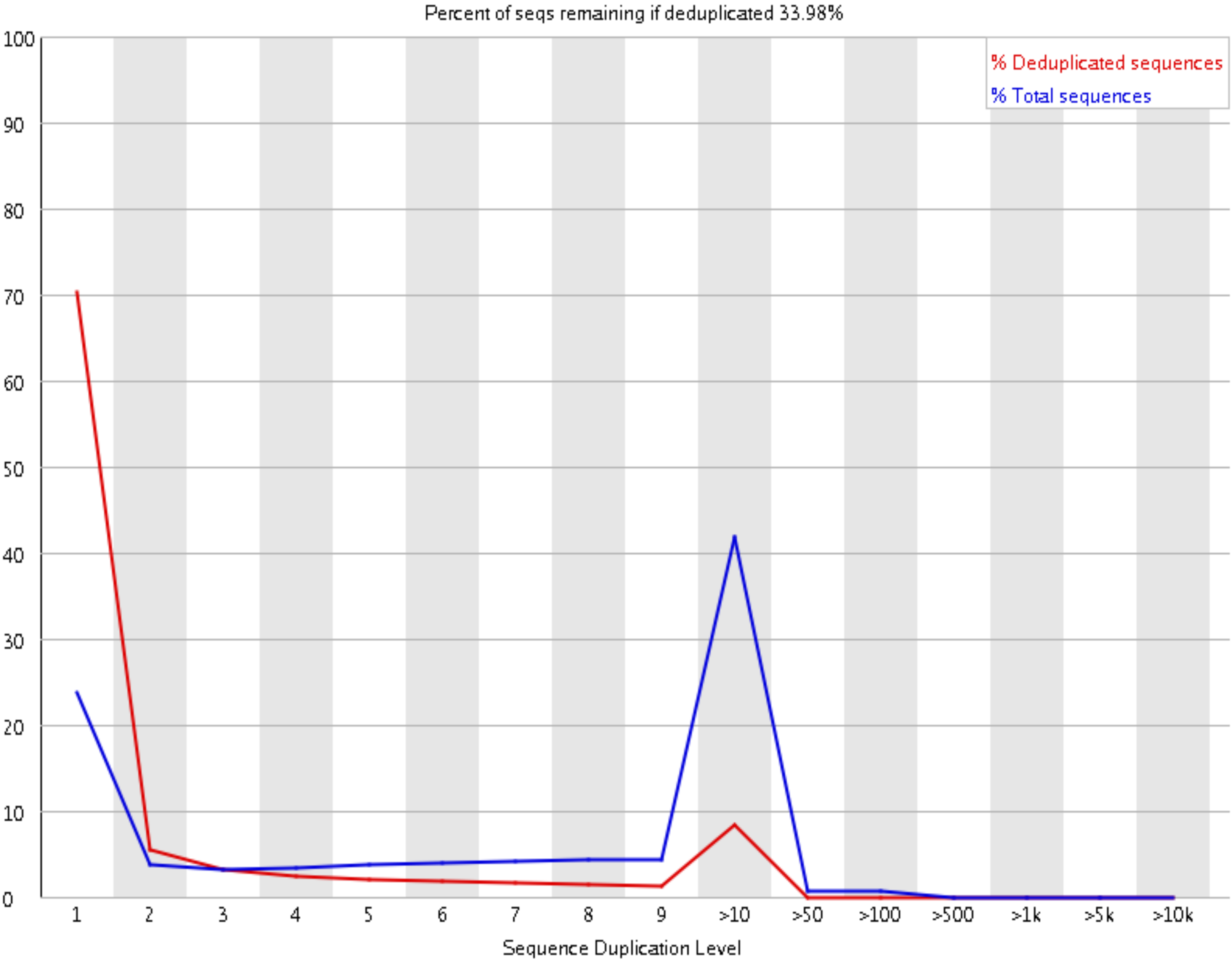

Overrepresented sequences

No overrepresented sequences

Adapter Content

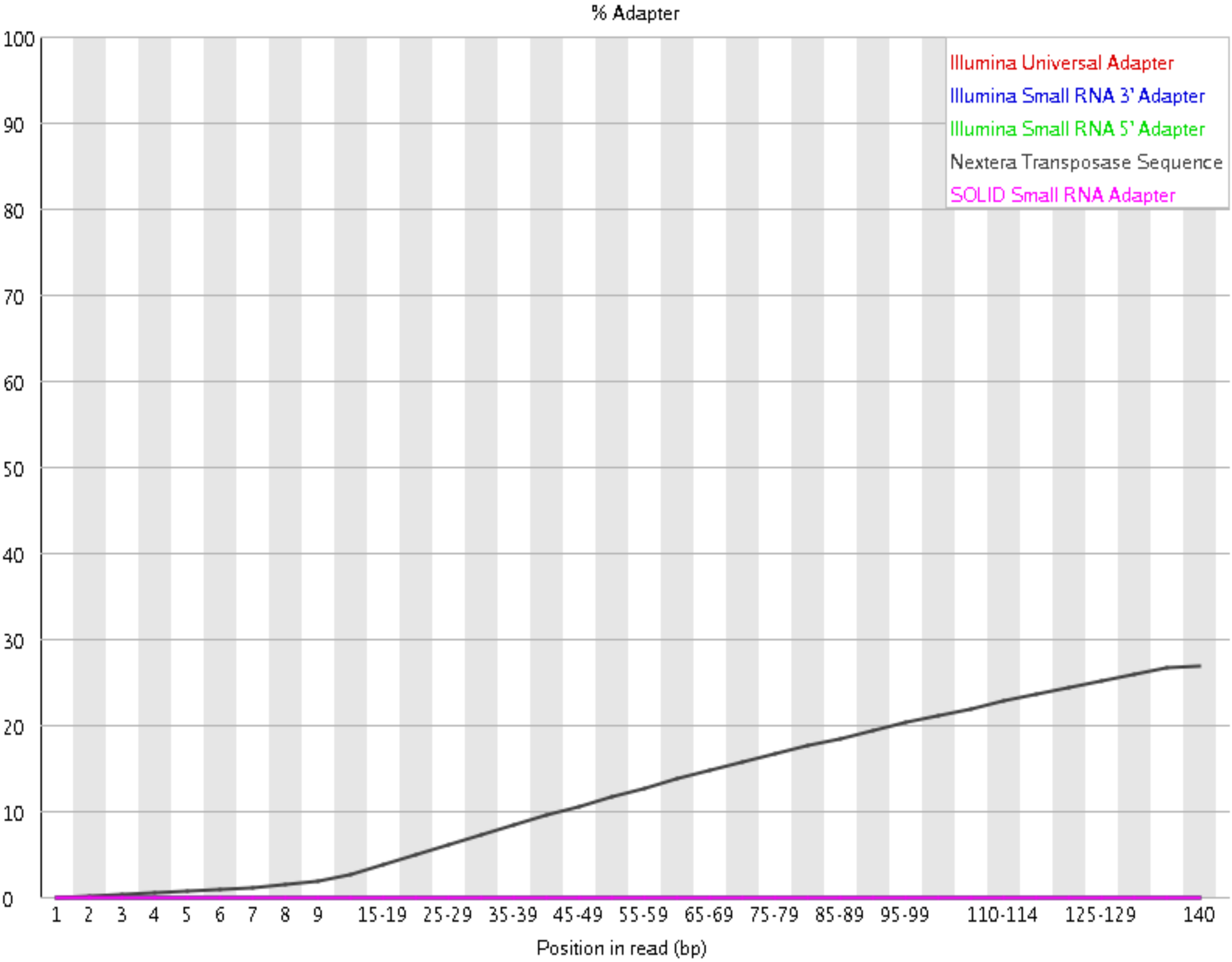

Produced by FastQC (version 0.11.7)

FastQC Report

Wed 28 Mar 2018
Venter_S1_merge_R1.fastq.gz

Summary

- Basic Statistics
- Per base sequence quality
- Per tile sequence quality
- Per sequence quality scores
- Per base sequence content
- Per sequence GC content
- Per base N content
- Sequence Length Distribution
- Sequence Duplication Levels
- Overrepresented sequences
- Adapter Content

Basic Statistics

Measure	Value
Filename	Venter_S1_merge_R1.fastq.gz
File type	Conventional base calls
Encoding	Sanger / Illumina 1.9
Total Sequences	789239544
Sequences flagged as poor quality	0
Sequence length	151
%GC	44

Per base sequence quality

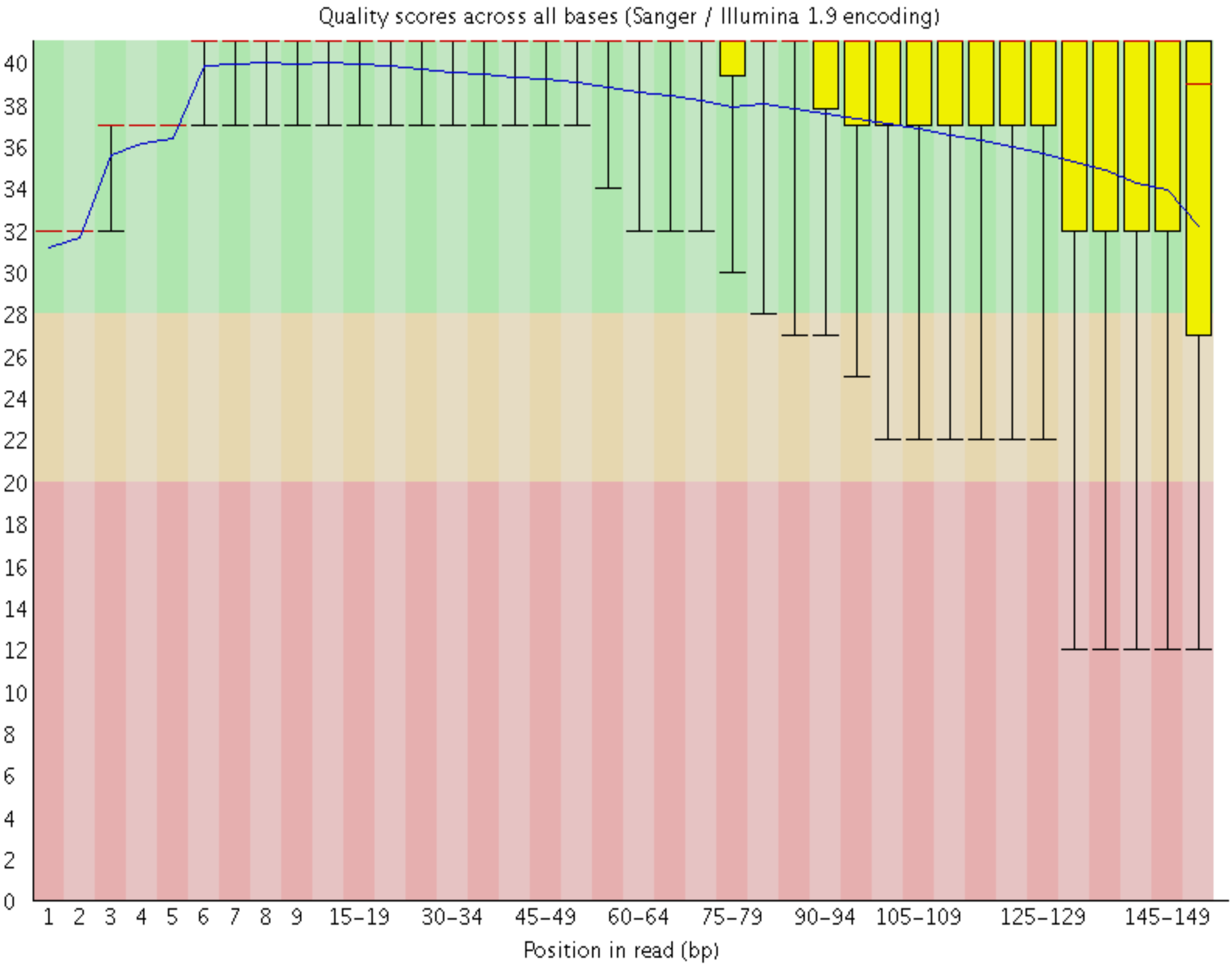

Per tile sequence quality

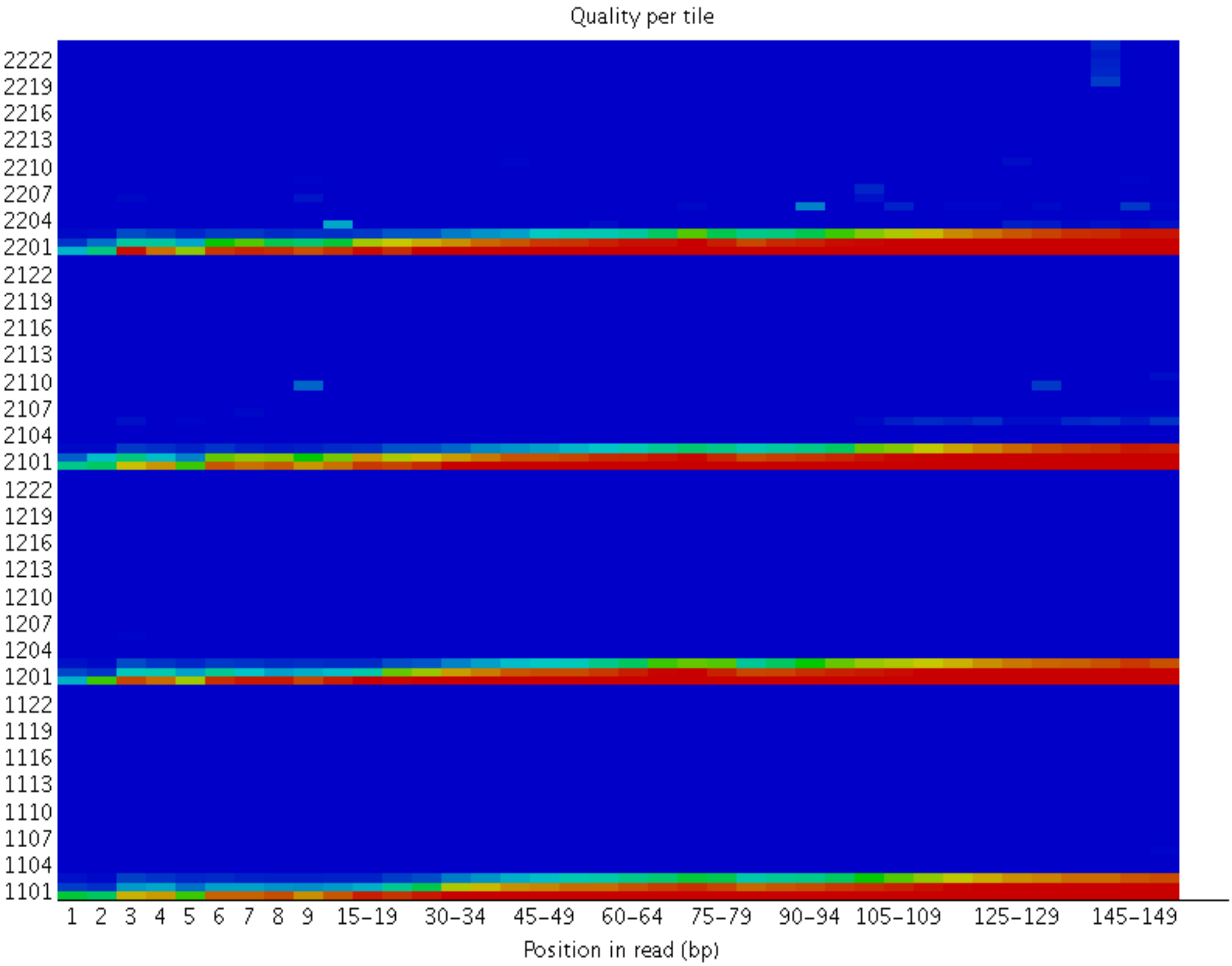

Per sequence quality scores

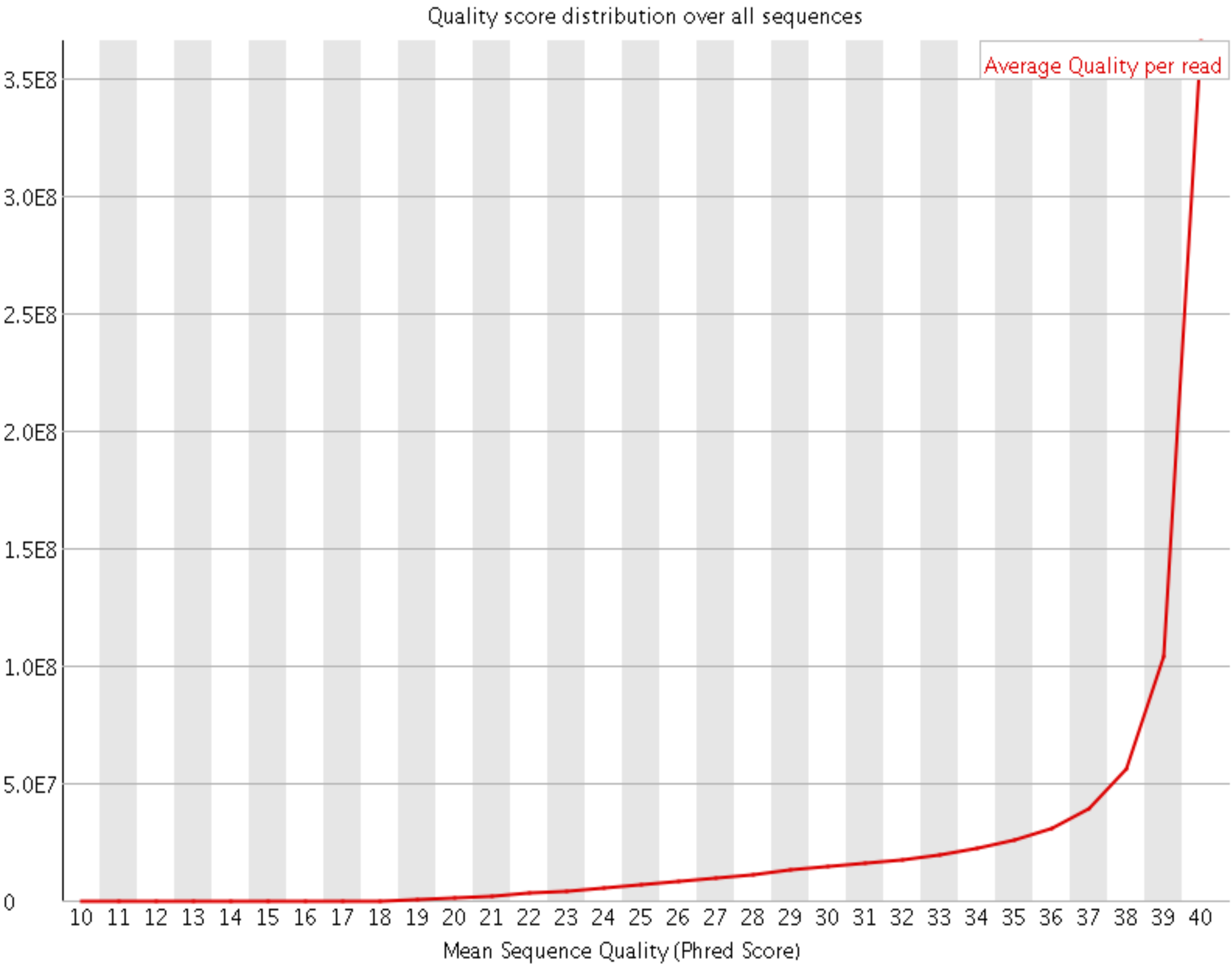

Per base sequence content

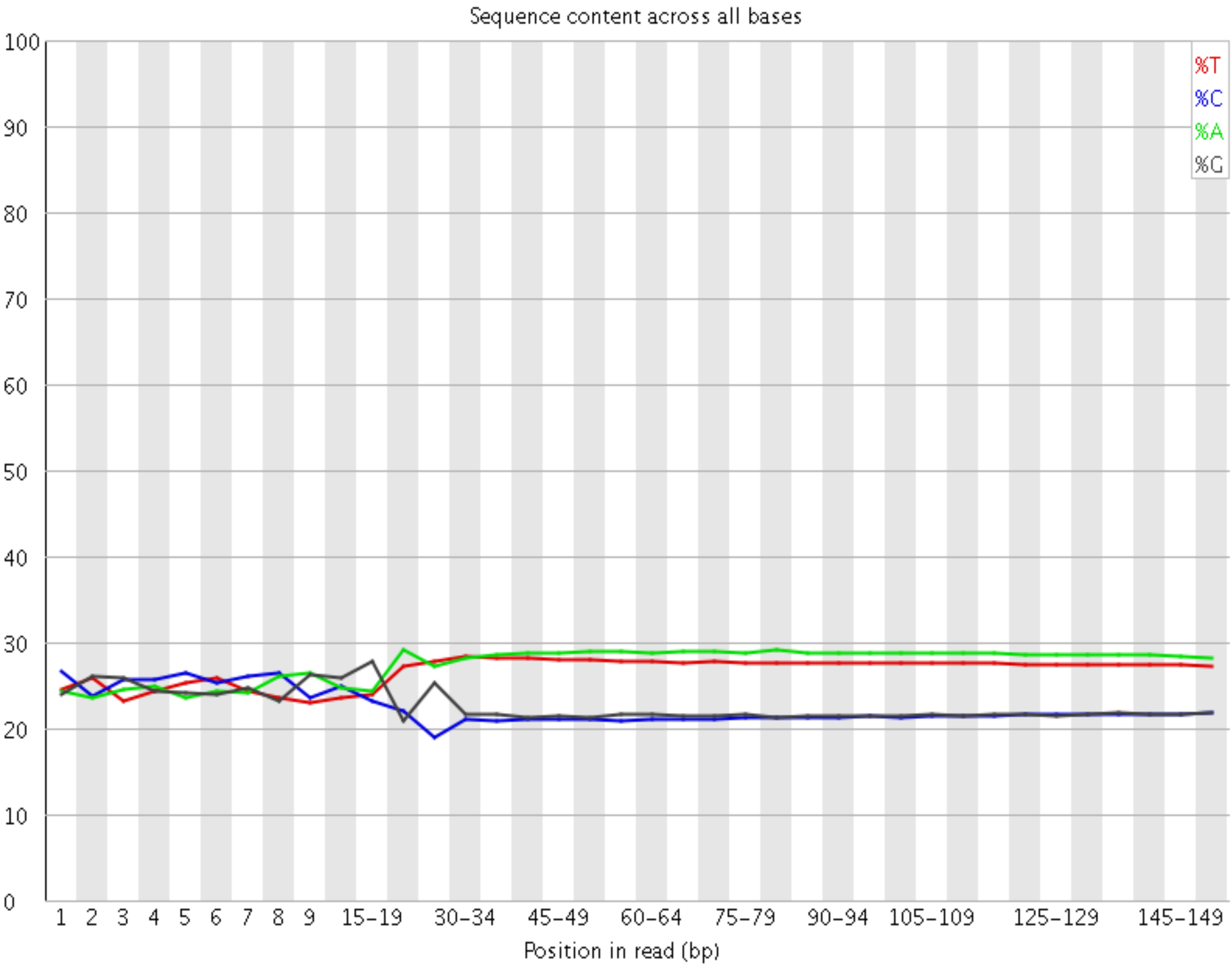

Per sequence GC content

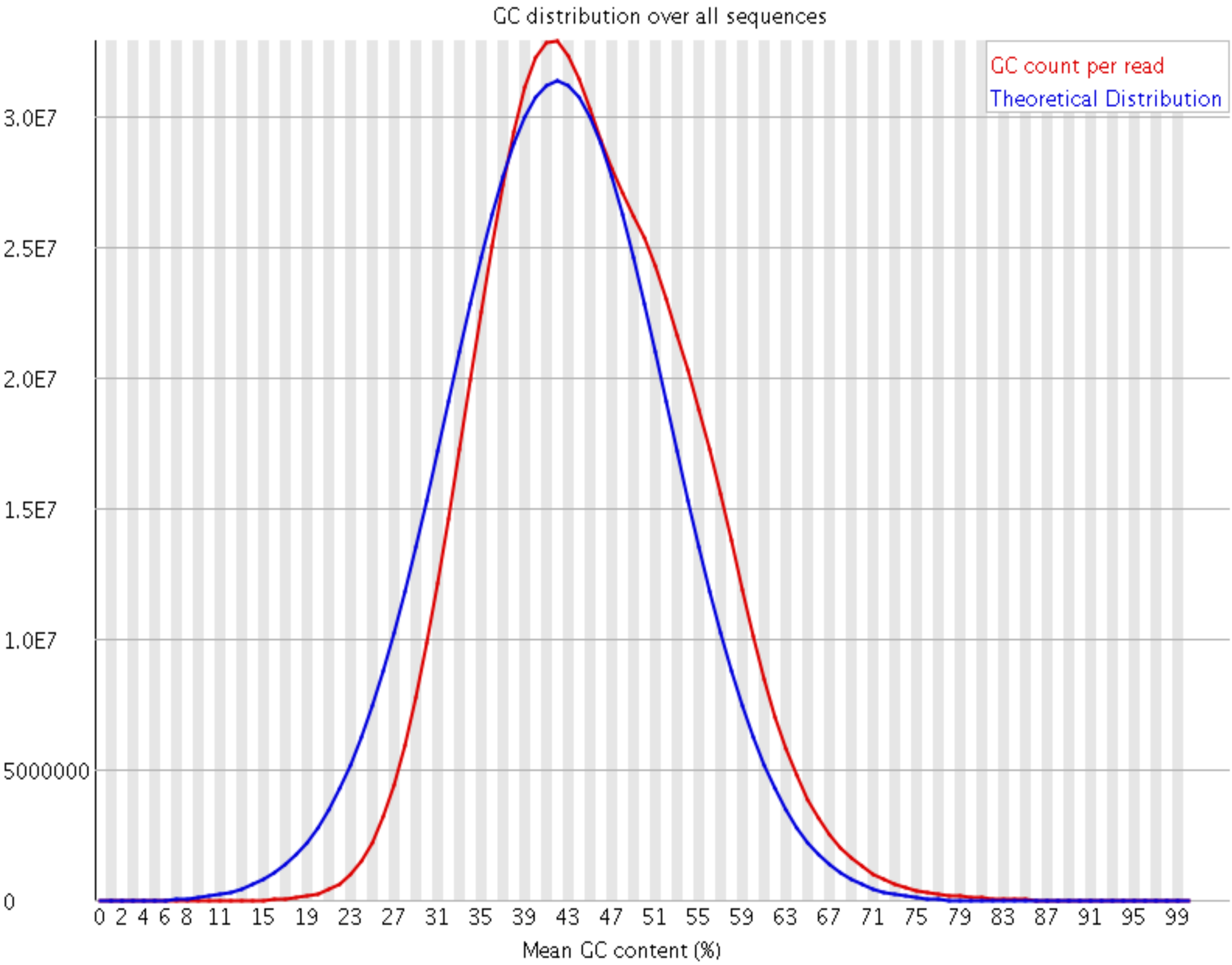

Per base N content

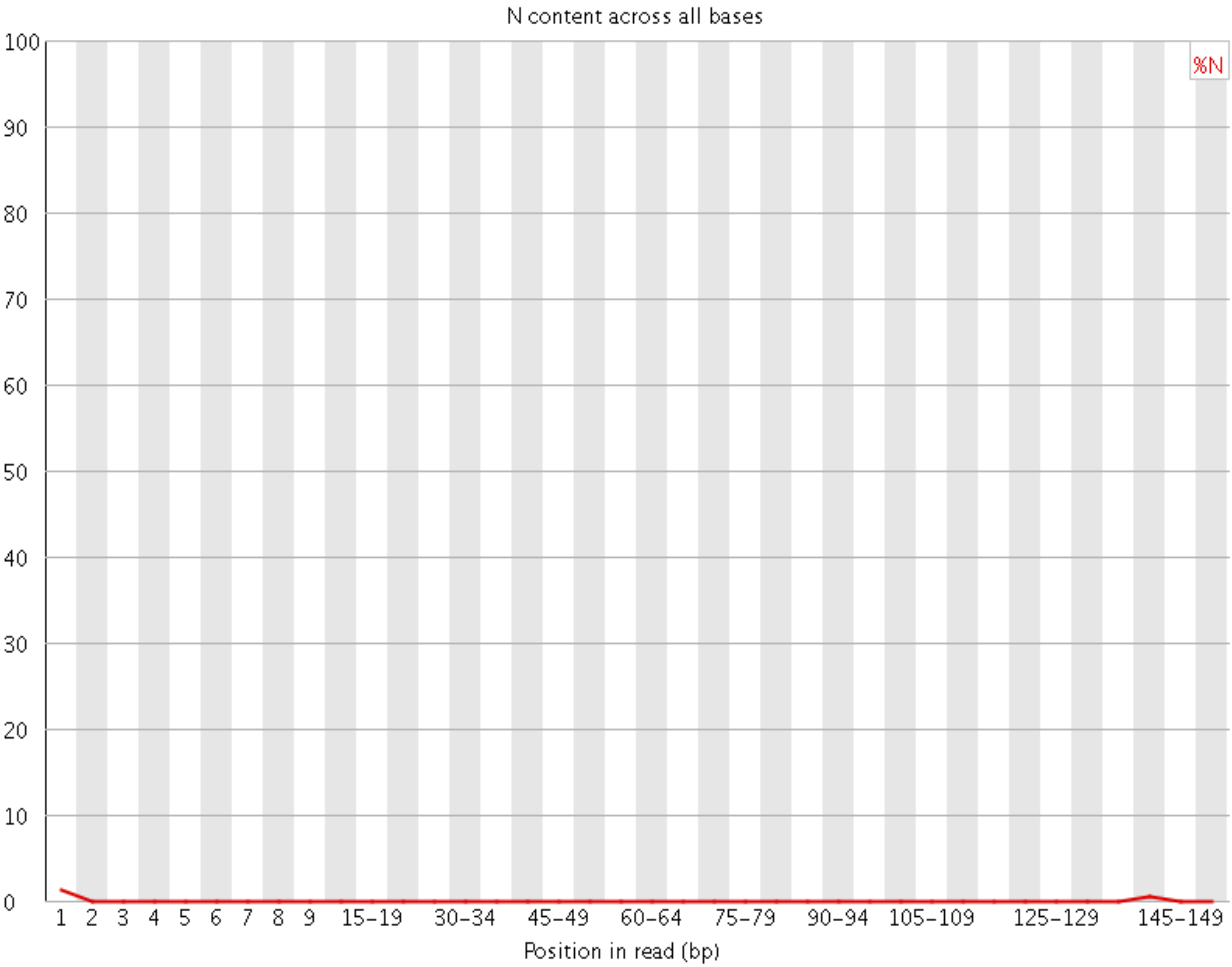

Sequence Length Distribution

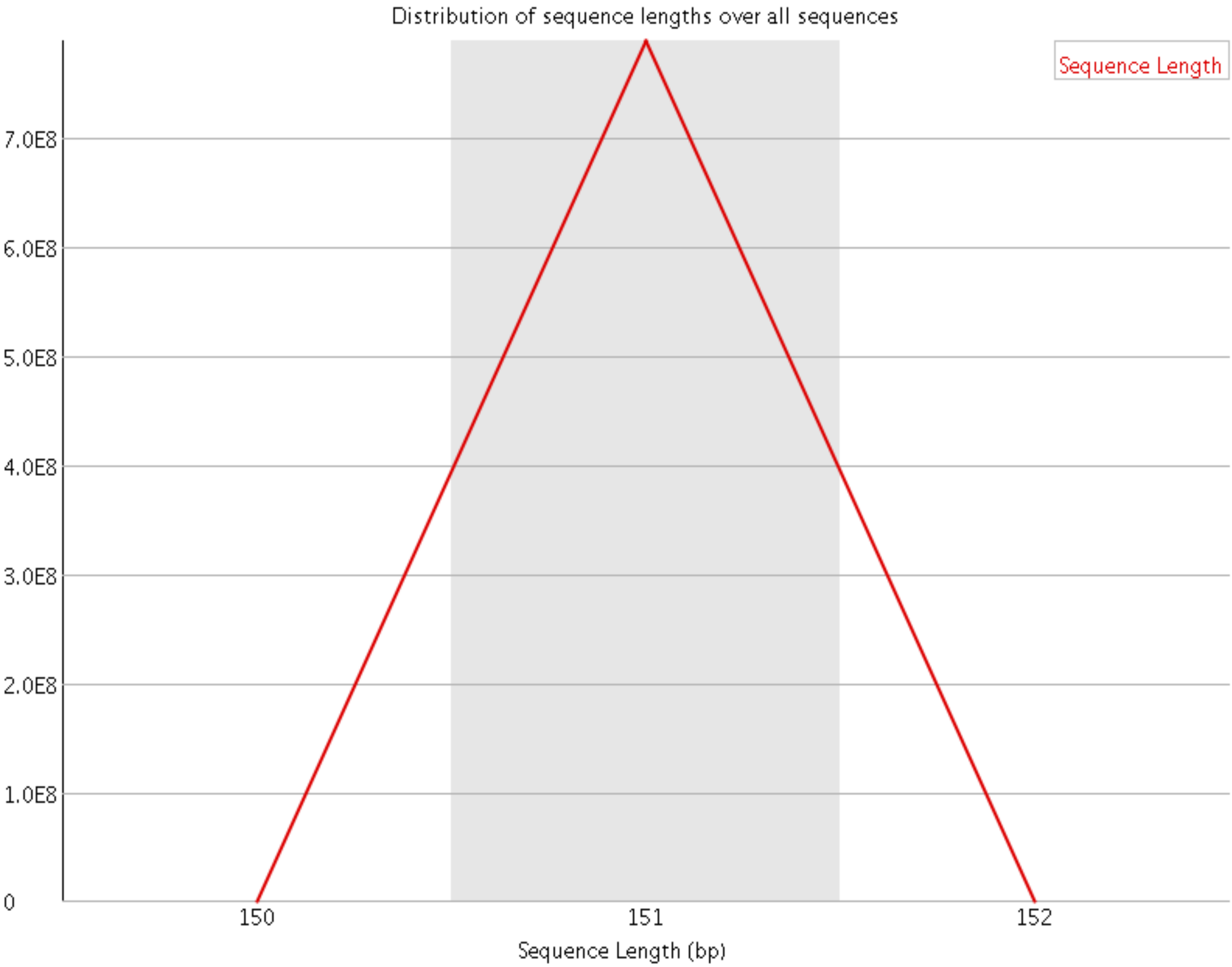

Sequence Duplication Levels

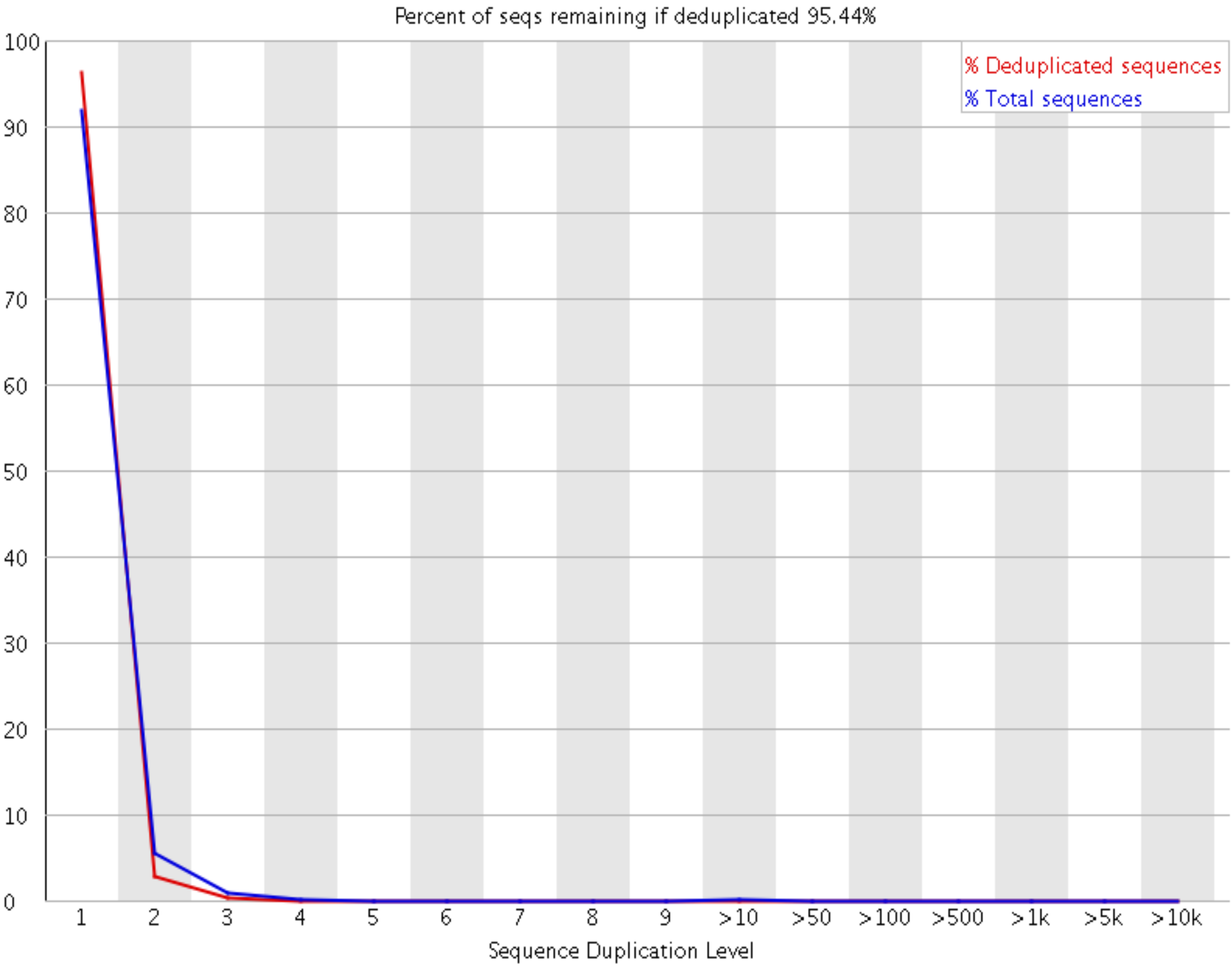

Overrepresented sequences

No overrepresented sequences

Adapter Content

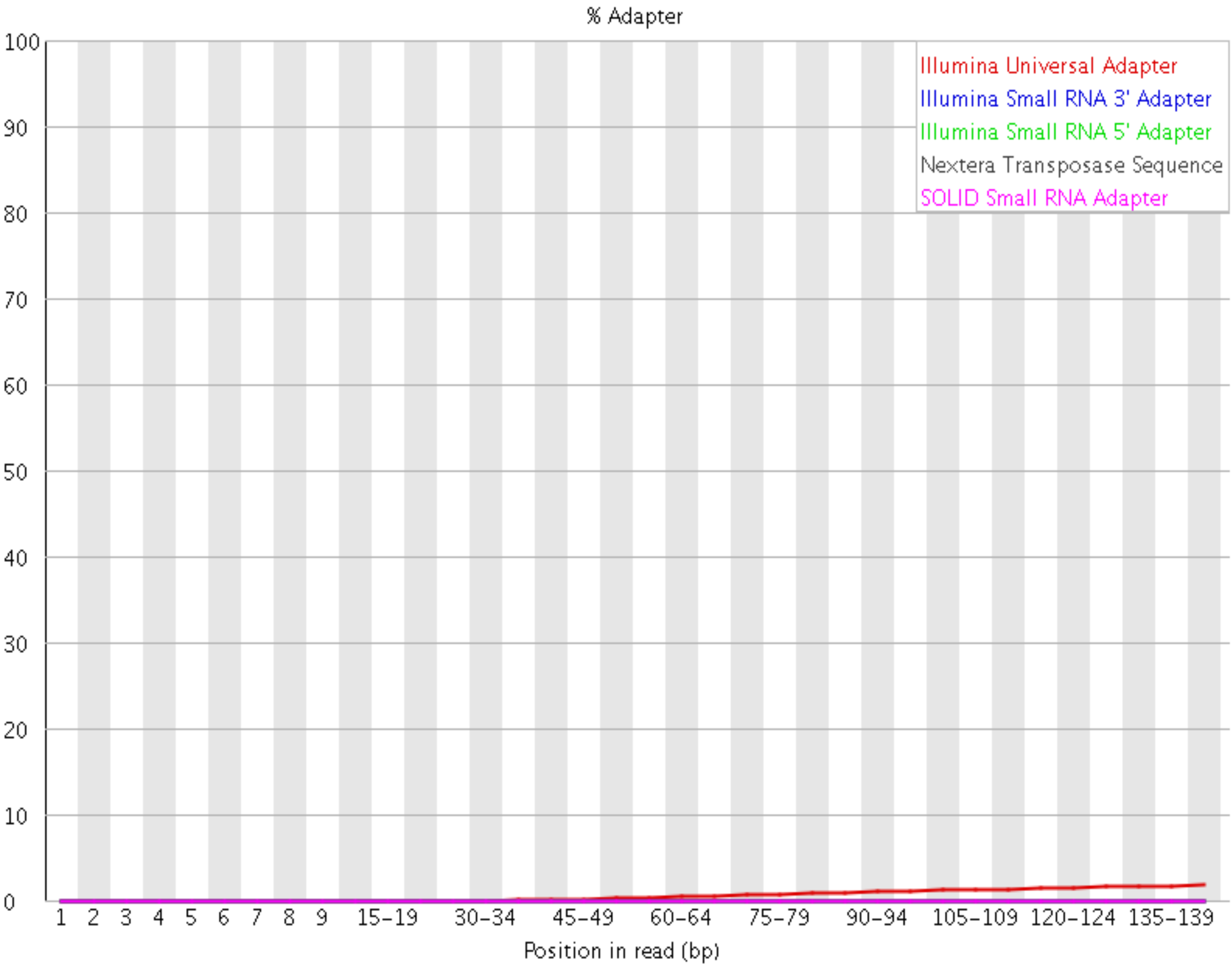

Produced by FastQC (version 0.11.7)

FastQC Report

Thu 29 Mar 2018
Venter_S1_merge_R2.fastq.gz

Summary

- Basic Statistics
- Per base sequence quality
- Per tile sequence quality
- Per sequence quality scores
- Per base sequence content
- Per sequence GC content
- Per base N content
- Sequence Length Distribution
- Sequence Duplication Levels
- Overrepresented sequences
- Adapter Content

Basic Statistics

Measure	Value
Filename	Venter_S1_merge_R2.fastq.gz
File type	Conventional base calls
Encoding	Sanger / Illumina 1.9
Total Sequences	789239544
Sequences flagged as poor quality	0
Sequence length	151
%GC	43

Per base sequence quality

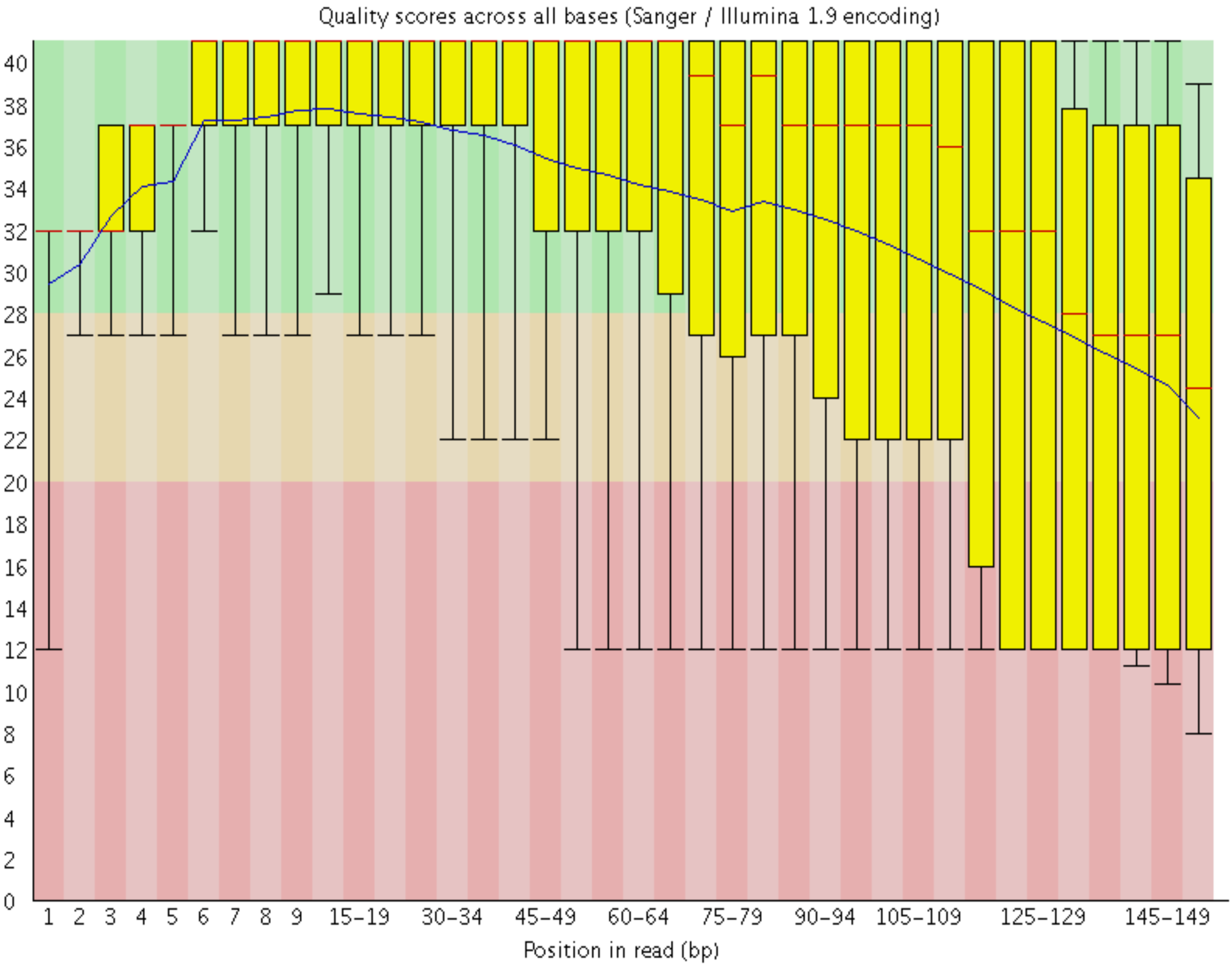

Per tile sequence quality

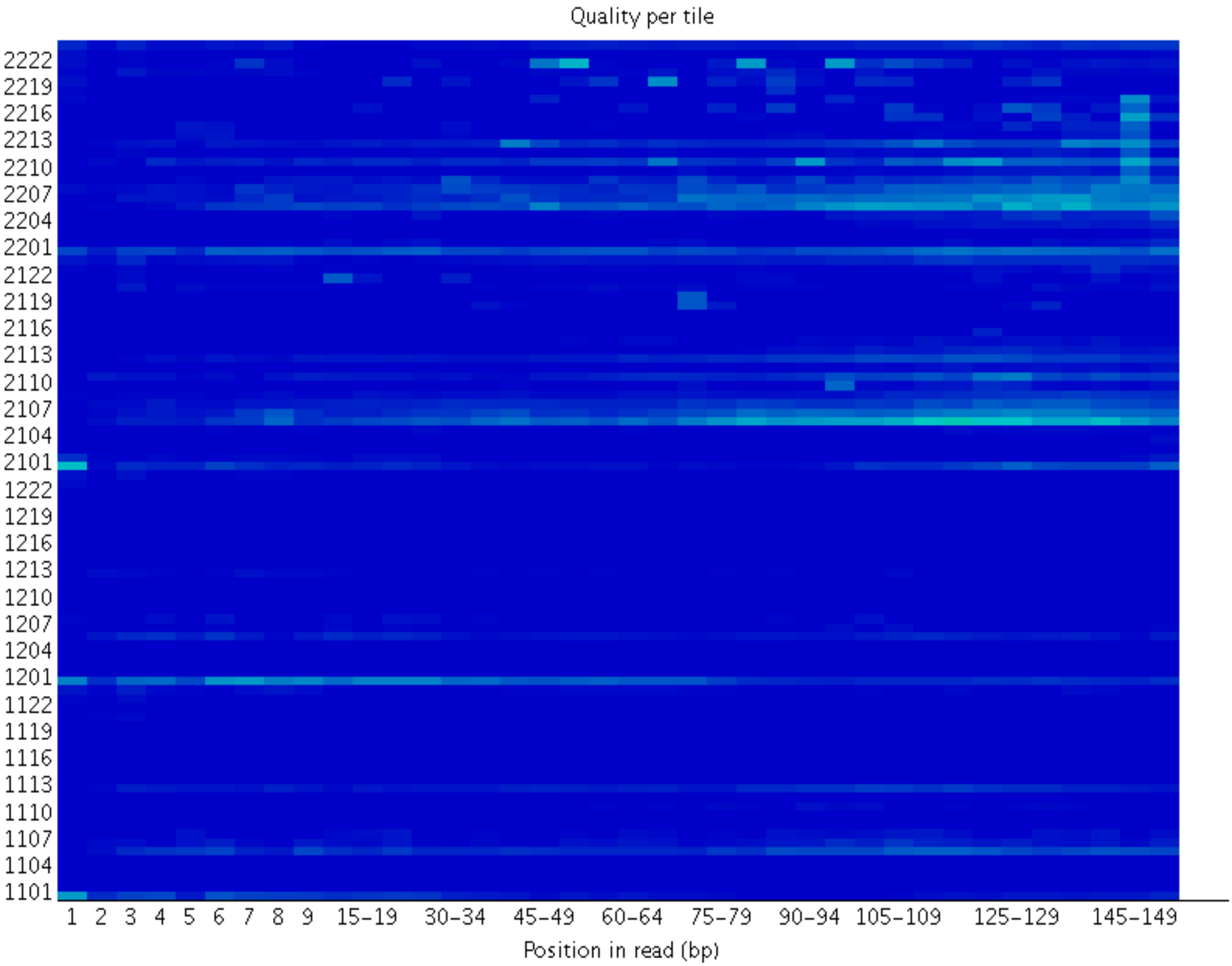

Per sequence quality scores

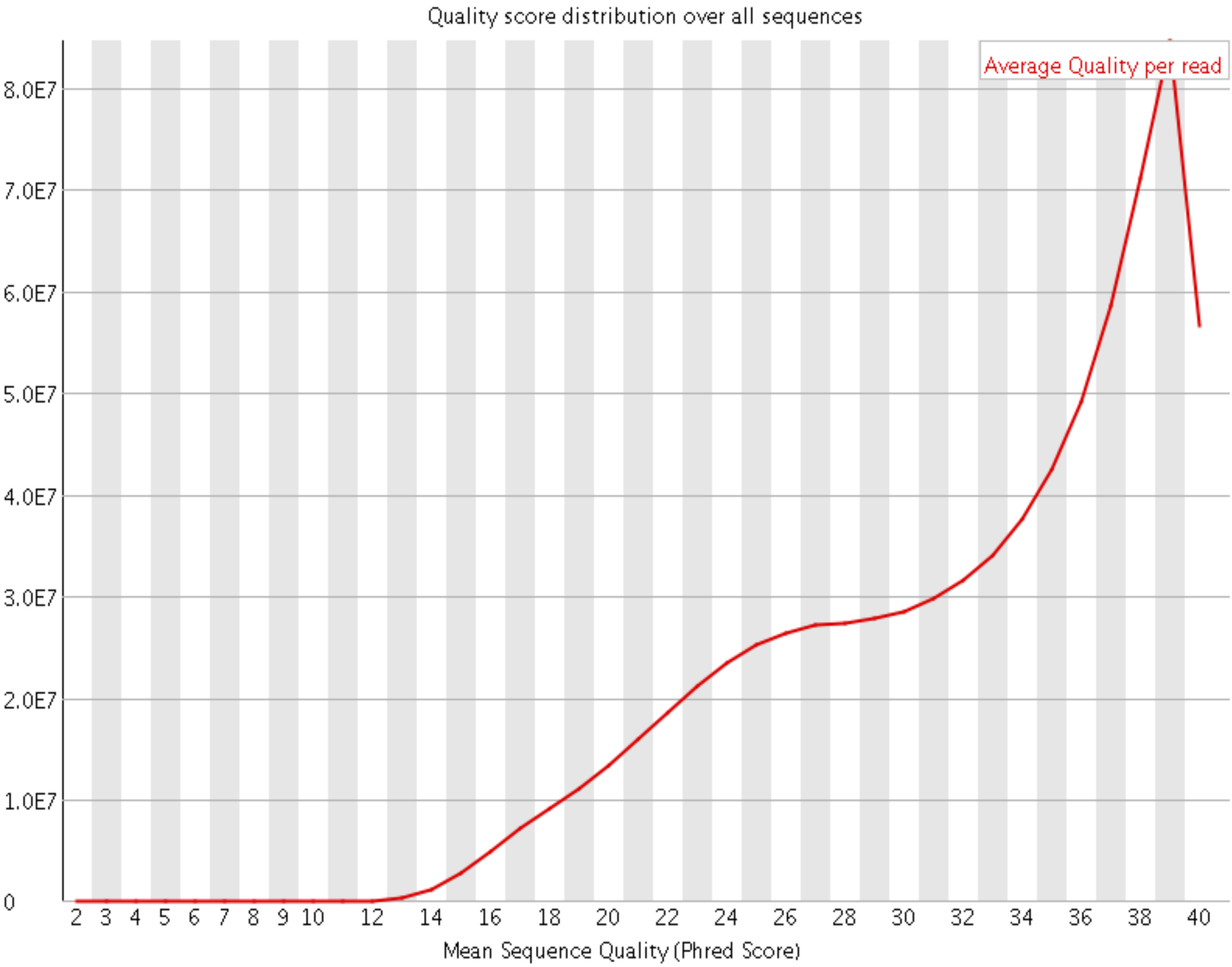

Per base sequence content

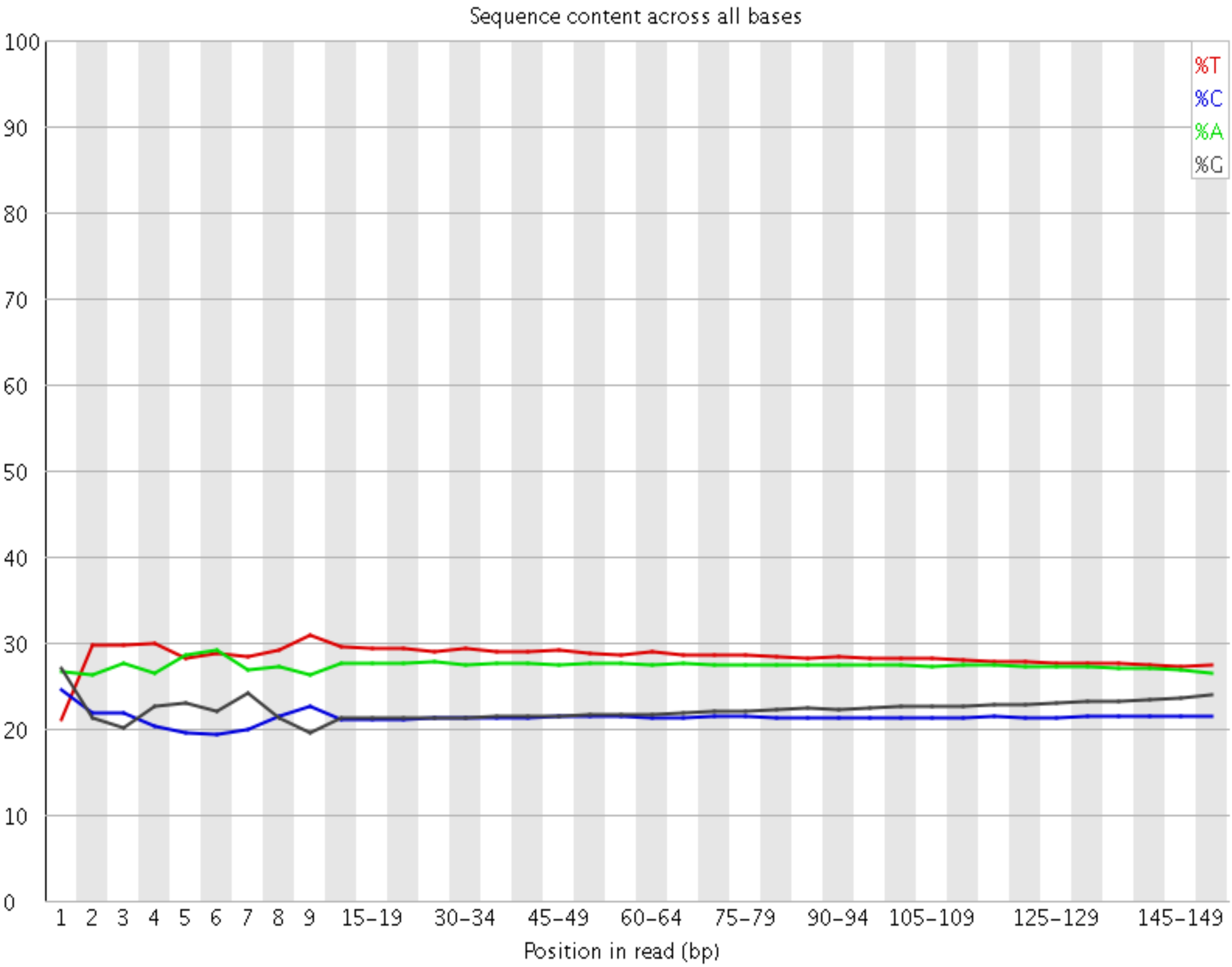

Per sequence GC content

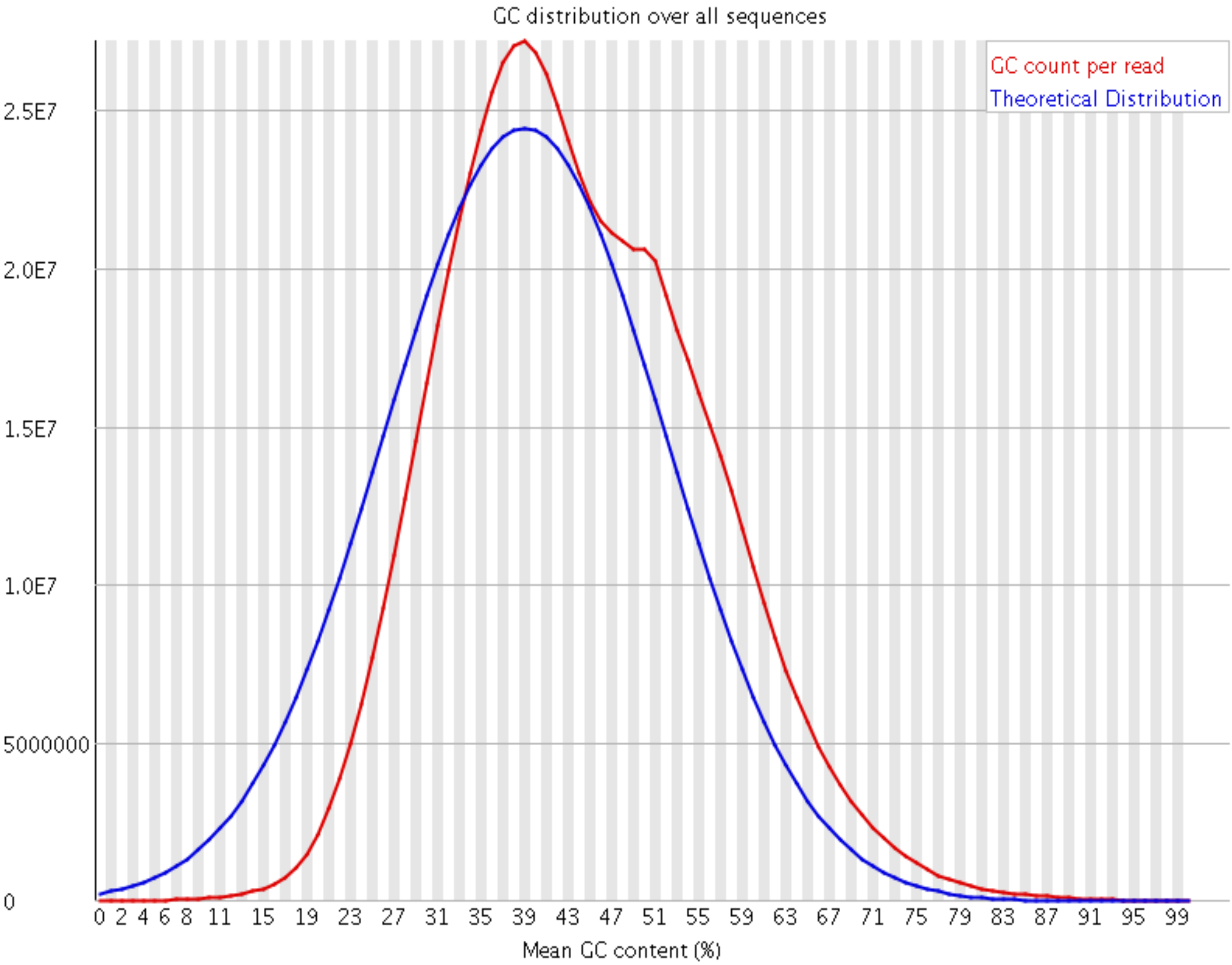

Per base N content

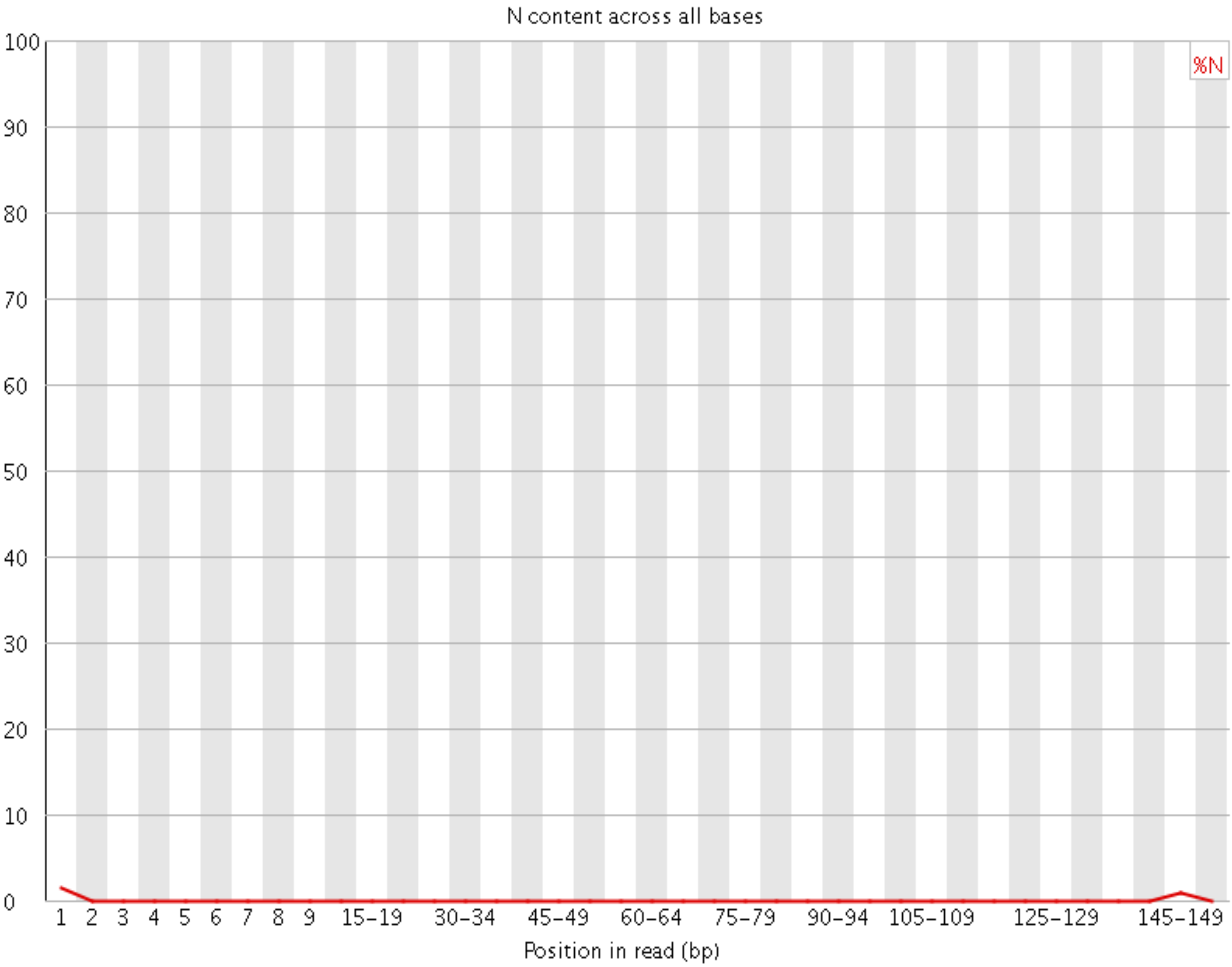

Sequence Length Distribution

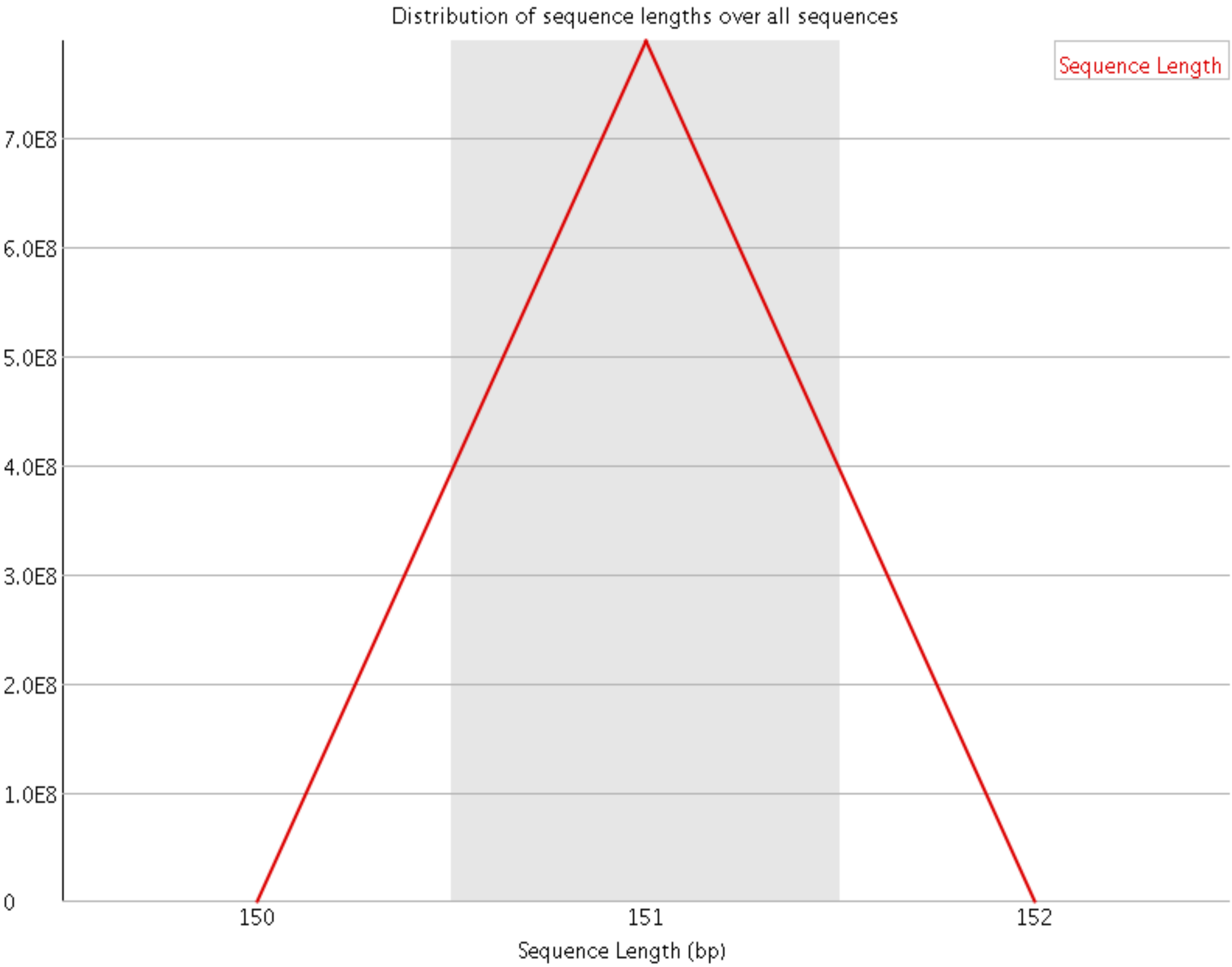

Sequence Duplication Levels

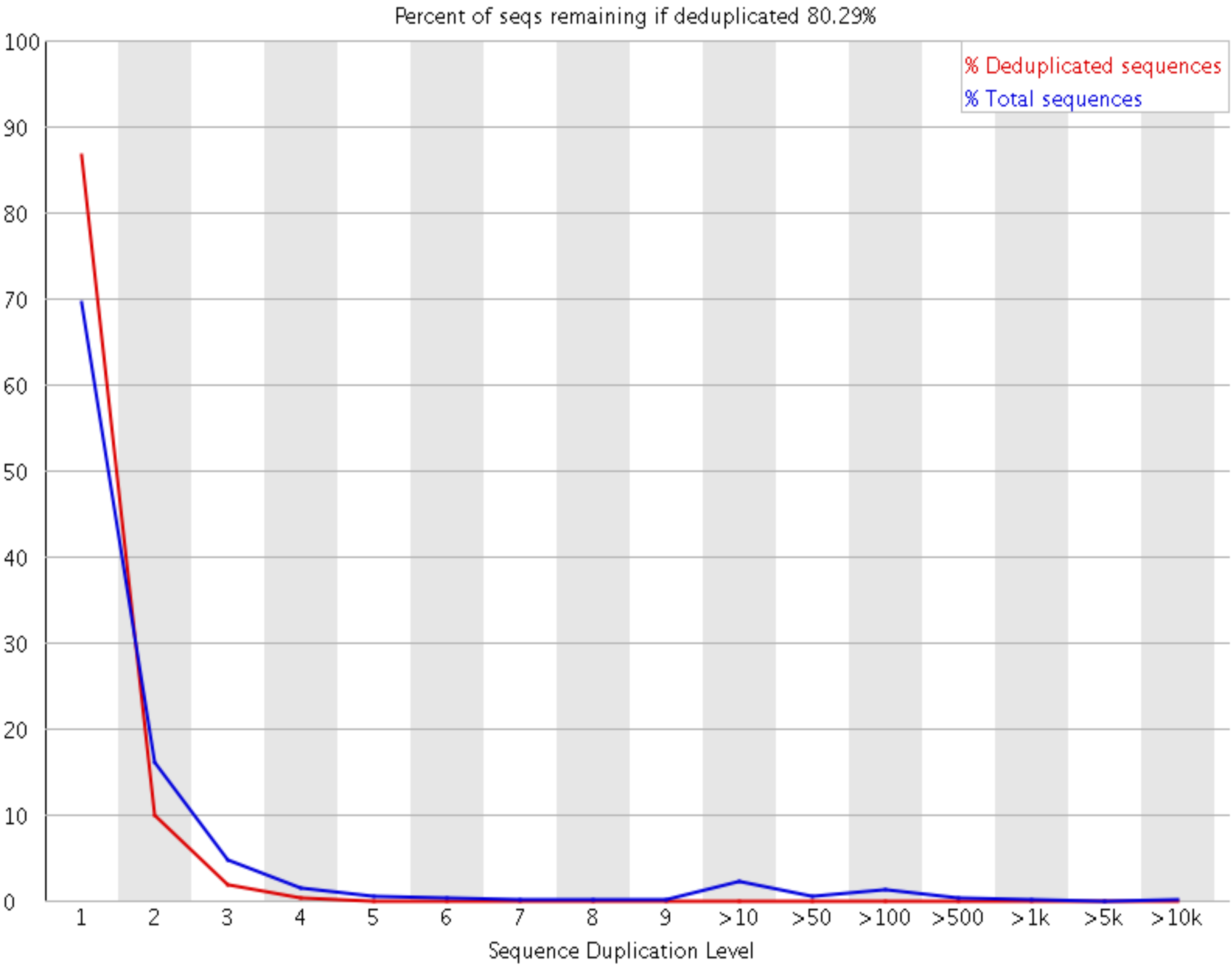

Overrepresented sequences

No overrepresented sequences

Adapter Content

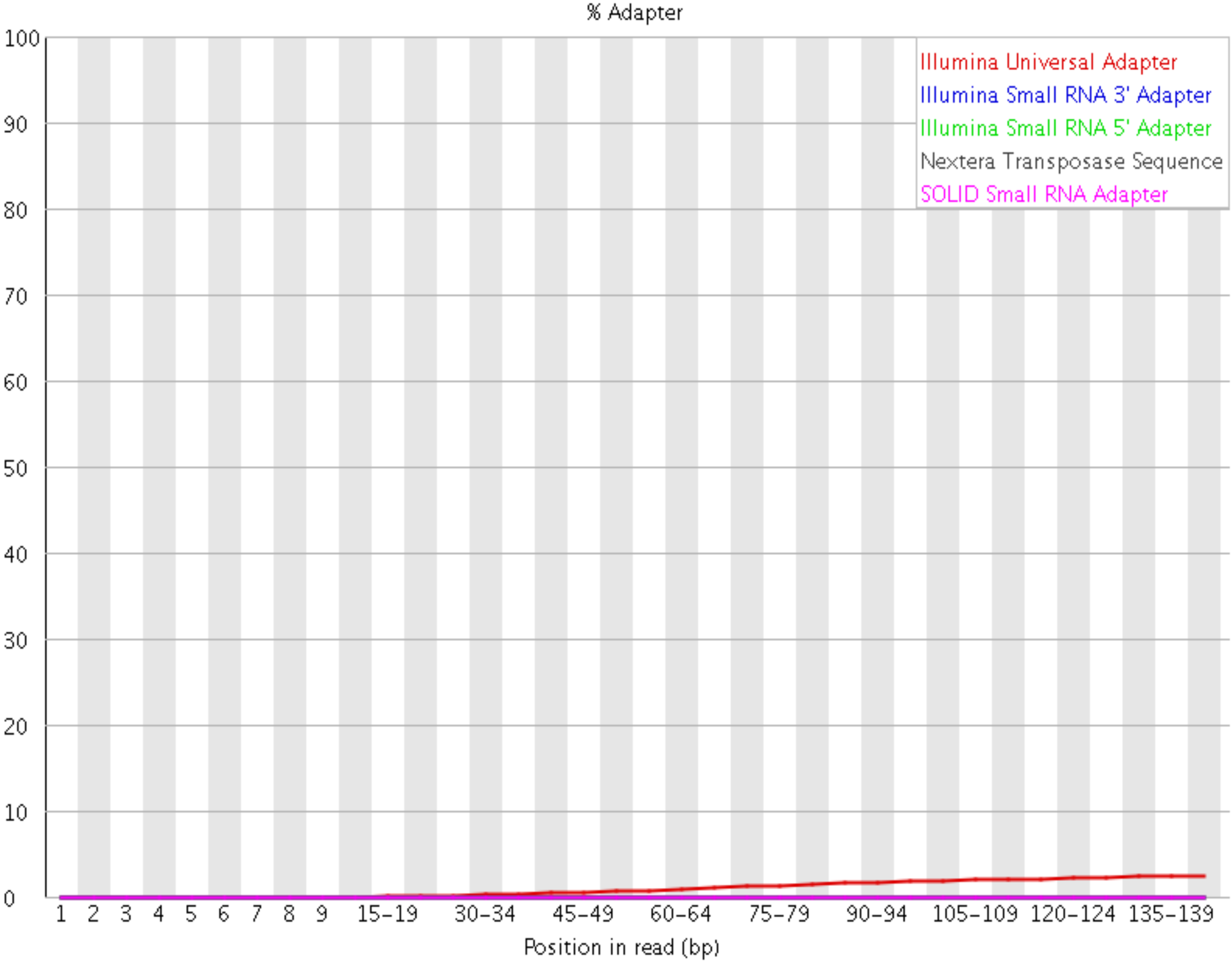

Produced by FastQC (version 0.11.7)
